# Developmental gene regulatory network connections predicted by machine learning from gene expression data alone

**DOI:** 10.1371/journal.pone.0261926

**Published:** 2021-12-28

**Authors:** Jingyi Zhang, Farhan Ibrahim, Emily Najmulski, George Katholos, Doaa Altarawy, Lenwood S. Heath, Sarah L. Tulin

**Affiliations:** 1 Department of Computer Science, Virginia Tech, Blacksburg, VA, United States of America; 2 Department of Biology, Canisius College, Buffalo, NY, United States of America; 3 Computer and Systems Engineering Department, Alexandria University, Alexandria, Egypt; Chang Gung University, TAIWAN

## Abstract

Gene regulatory network (GRN) inference can now take advantage of powerful machine learning algorithms to complement traditional experimental methods in building gene networks. However, the dynamical nature of embryonic development–representing the time-dependent interactions between thousands of transcription factors, signaling molecules, and effector genes–is one of the most challenging arenas for GRN prediction. In this work, we show that successful GRN predictions for a developmental network *from gene expression data alone* can be obtained with the Priors Enriched Absent Knowledge (PEAK) network inference algorithm. PEAK is a noise-robust method that models gene expression dynamics via ordinary differential equations and selects the best network based on information-theoretic criteria coupled with the machine learning algorithm Elastic Net. We test our GRN prediction methodology using two gene expression datasets for the purple sea urchin, *Stronglyocentrotus purpuratus*, and cross-check our results against existing GRN models that have been constructed and validated by over 30 years of experimental results. Our results find a remarkably high degree of sensitivity in identifying known gene interactions in the network (maximum 81.58%). We also generate novel predictions for interactions that have not yet been described, which provide a resource for researchers to use to further complete the sea urchin GRN. Published ChIPseq data and spatial co-expression analysis further support a subset of the top novel predictions. We conclude that GRN predictions that match known gene interactions can be produced using gene expression data alone from developmental time series experiments.

## Introduction

Transcription factors regulate cell-specific gene expression to create phenotypes, respond to disease, drive evolution, and guide embryonic development [1]. Taken together as a system, gene regulation can be organized and modeled as a hierarchical network, a Gene Regulatory Network (GRN), as first proposed by Davidson [2, 3]. GRN models are now routinely used to follow the causal links from regulatory genes to cell fate decisions or cell activities. Using a GRN model to create hypotheses about the function of actors in a regulatory program aids in experimental design.

Animal and plant developmental programs have been described by GRN models, beginning with the construction of the sea urchin endomesoderm GRN [3, 4] and followed soon thereafter by the *Drosophila melanogaster* dorsoventral patterning network [5, 6] and the *Xenopus laevis* mesoderm specification network [7]. Traditionally, GRNs are compiled through extensive experimental perturbations, often involving a combination of knockdown techniques and visualization of changes in gene expression. Accuracy of the GRN is improved when further experiments confirm *cis-*regulatory interactions at the level of transcription factor binding sites. However, there is now a pressing need to facilitate GRN modeling using computational prediction tools to help fill in the gaps in existing GRNs and to help create new GRN models with testable predictions.

GRN prediction algorithms from gene expression data alone have been proposed [8], and several have been compared and tested through the DREAM consortium [9]. The overall accuracy of these previous methods using gene expression data alone is at or below 50% even with a consensus of several computational methods. GRN inference methods assessed by the DREAM consortium were designed to infer network relationships from unicellular organisms and *in silico* data. The goal of the approach described in this manuscript is to improve the sensitivity of GRN prediction while performing GRN inference on multicellular organisms during the dynamic process of embryonic development. Developmental GRNs are inherently challenging to predict due to the temporal and spatial transcriptional complexity inherent in the developmental process they seek to model. In the decade since the initial DREAM network inference challenge, next generation sequencing and machine learning algorithms have become increasingly sophisticated, and new approaches have emerged with solutions to address more challenging GRN inference problems.

To address the significant challenge of GRN inference in multicellular organisms during development, we used the Priors Enriched Absent Knowledge (PEAK) network inference algorithm to reconstruct GRN interactions [10]. PEAK uses differential equations, context likelihood of relatedness (CLR), and the machine learning method Elastic Net to predict the most likely interactions between transcription factors and target genes. The execution of PEAK consists of two phases, a coarse-grained phase and a fine-grained phase, to predict network interactions. In the coarse-grained phase, potential regulators for each gene are extracted using mixed context likelihood of relatedness (mixed CLR). In the fine-grained phase, two modified versions of Elastic Net are employed to refine the predictions and to integrate curated or noisy prior knowledge, when available. Prior knowledge about the network can also be added if available; however, to be most broadly applicable across established and emerging model systems, we used PEAK without prior knowledge in this study.

To test GRN inference in a multicellular developmental context, we chose two sea urchin embryonic GRNs governing endomesoderm and ectoderm specification as test networks because they are widely regarded as some of the most well-supported developmental GRN models with many *cis*-regulatory interactions verified at the base-pair level. We also obtained two sea urchin gene expression datasets to use as input. The California purple sea urchin, *Strongylocentrotus purpuratus*, is a marine invertebrate. The sea urchin is a member of the phylum Echinodermata, which, along with the Hemichordata, are the closest known sister groups to the Chordates, the phylum to which humans belong. The genome of *S*. *purpuratus* was sequenced in 2006, which produced an estimated gene count of ~23,000 [11]. Later transcriptome sequencing found evidence for ~21,000 gene models [12]. *S*. *purpuratus* develops rapidly from a single cell (the fertilized egg) to a late gastrula embryo in 48 hours at 14°C and then into a prism-shaped larva by 72 hours. Over the last 20 years, GRN models describing the regulation of embryonic development of *S*. *purpuratus* have been built by experimentation and collaboration of sea urchin researchers around the world. The GRN models describing development are divided into the network describing the ectodermal tissue layer [13, 14], which will give rise to the nervous system and outer tissues of the larvae, and the network controlling the endomesodermal tissue layer, which will give rise to the gut and the larval skeleton [3, 4]. The two GRN models are hosted on the BioTapestry website, which is also the home of the open source platform used to construct and visualize these models [15].

The goal of our approach is to successfully employ PEAK to predict gene regulatory interactions using only whole embryo temporal gene expression data. The specific motivation for testing PEAK using gene expression data only is to develop a pipeline that is most broadly applicable to researchers investigating all kinds of metazoan species. For many emerging model systems, traditional transcriptomic gene expression data from developmental time series are readily available but extensive omics data sets describing spatial expression, transcription factor binding sites, ChIPseq, functional gene annotation, and proteomics are often lacking. Therefore, there is a demonstrated need for a GRN inference approach based on temporal gene expression data alone to make use of existing transcriptomic sequencing data and to guide future experiments on regulatory interactions during embryonic development [16, 17]. The PEAK method is also applicable for researchers in possession of additional prior information, including single-cell RNAseq experiments and other types of omics datasets.

Our method using PEAK on time series gene expression data describing sea urchin development was able to achieve a maximum of 81.58% sensitivity using 32 experiments. In comparison, previous large-scale assessment of network inference methods aimed at predicting gene networks using gene expression data alone in unicellular organisms found a maximum of 50% precision using 800 microarray experiments and much less accuracy using 300 experiments [9].

## Results

GRN models are concerned with genes whose expression is regulated and the regulators themselves. To identify the set of regulated genes to input into the PEAK machine learning algorithm, we started with the sea urchin RNAseq transcriptome dataset covering 0-72hpf, which represents genes expressed during embryogenesis [18]. The RNAseq dataset was filtered to identify the set of transcripts that are differentially expressed during embryonic development and are regulative in nature.

### Differential gene expression analysis

**Fig 1:**
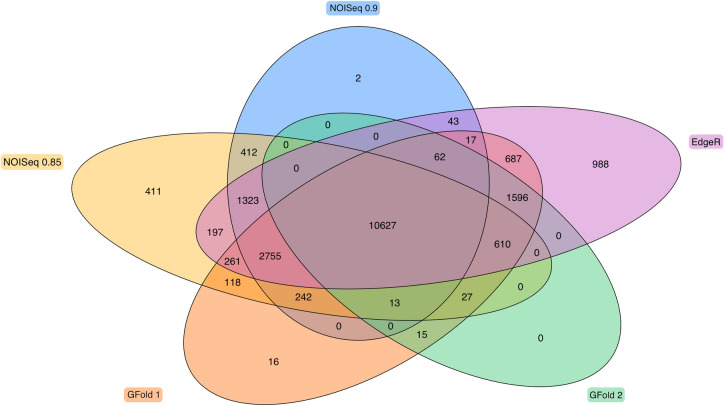
Differentially expressed genes. Intersection of unique differentially expressed genes determined by NOISeq, EdgeR and GFold. The intersection of all 5 methods contains 10,627 unique genes specified as differentially expressed as seen where all the ovals overlap in the center. The number of genes uniquely described as DE by each program is the outermost number closest to the label, 2 for NOISeq (*λ*_1_ = 0.9), 411 for NOISeq (*λ*_1_ = 0.85), 16 for GFold1 (λ_3_ = ∓ 1), 0 for GFold2 (*λ*_4_ = ∓ 1.5), and 988 for EdgeR.

We employed three programs (NOISeq, EdgeR, and GFold) to determine the set of differentially expressed genes (DEGs) with the parameters described in the methods section [19–21]. There was variation in the number of DEGs determined by NOISeq, EdgeR, and GFold in the RNAseq dataset. We compared the overlap of genes above the threshold identified by each method to obtain a core set of DEGs (Fig 1). The core set contained 10,627 genes that were consistently specified as differentially expressed no matter which method was used. This figure is in line with a previous analysis that found that 10,800 of ~16,700 genes expressed during sea urchin embryogenesis showed changes in relative abundance [18]. We found that the result from NOISeq (λ_1_ = 0.9) contained the most overlap and the least difference with results from the other methods, while maintaining a more selective number of genes determined to be differentially expressed (see Table 1 where a total of 5 methods are detailed). Only .01% of the genes determined by NOISeq (λ_1_ = 0.9) are unique to that method; in contrast, more than 5% of the genes from the result of EdgeR are unique to EdgeR. All the genes determined by GFold (λ_4_ = ∓ 1.5) overlapped with the results from other methods, but the GFold DEG set was missing 2,755 genes that were identified as differentially expressed by the other 4 methods.

**Table 1:** Differential gene expression analysis.

Program	Total DEGs	# Unique Genes	% Unique Genes	# Overlap Genes	% Overlap Genes
NOISeq (λ_1_ = 0.9)	15496	2	0.01%	15494	99.99%
NOISeq (λ_2_ = 0.85)	16996	411	2.42%	16585	97.58%
GFold 1 (λ_3_ = ∓ 1)	17046	16	0.09%	17030	99.91%
GFold 2 (λ_4_ = ∓ 1.5)	12950	0	0.00%	12950	100.00%
EdgeR	19166	988	5.15%	18178	94.85%

Differential gene expression analysis results summary from three programs (NOISeq, GFold, and EdgeR) using two different thresholds for NOISeq and GFold.

### Gene ontology filter

NOISeq reduced the 21,092 total transcripts to a set of 15,496 differentially expressed transcripts. However, this set of DEGs is still too large to be effectively used as input into the PEAK prediction algorithm. Therefore, we applied a second filter to the gene set to achieve a more appropriate number of regulatory genes. The second filter was a Gene Ontology (GO) filter for genes related to transcription and signaling. We used the custom GO annotation generated by the authors of the transcriptome [12]. The GO filter identified 1,038 transcripts that are regulatory in nature, of which 544 were also identified as differentially expressed (S1 File). The 544 transcripts represent 504 unique gene models annotated by a single gene identifier (SPU_ID).

### PEAK analysis

**Fig 2:**
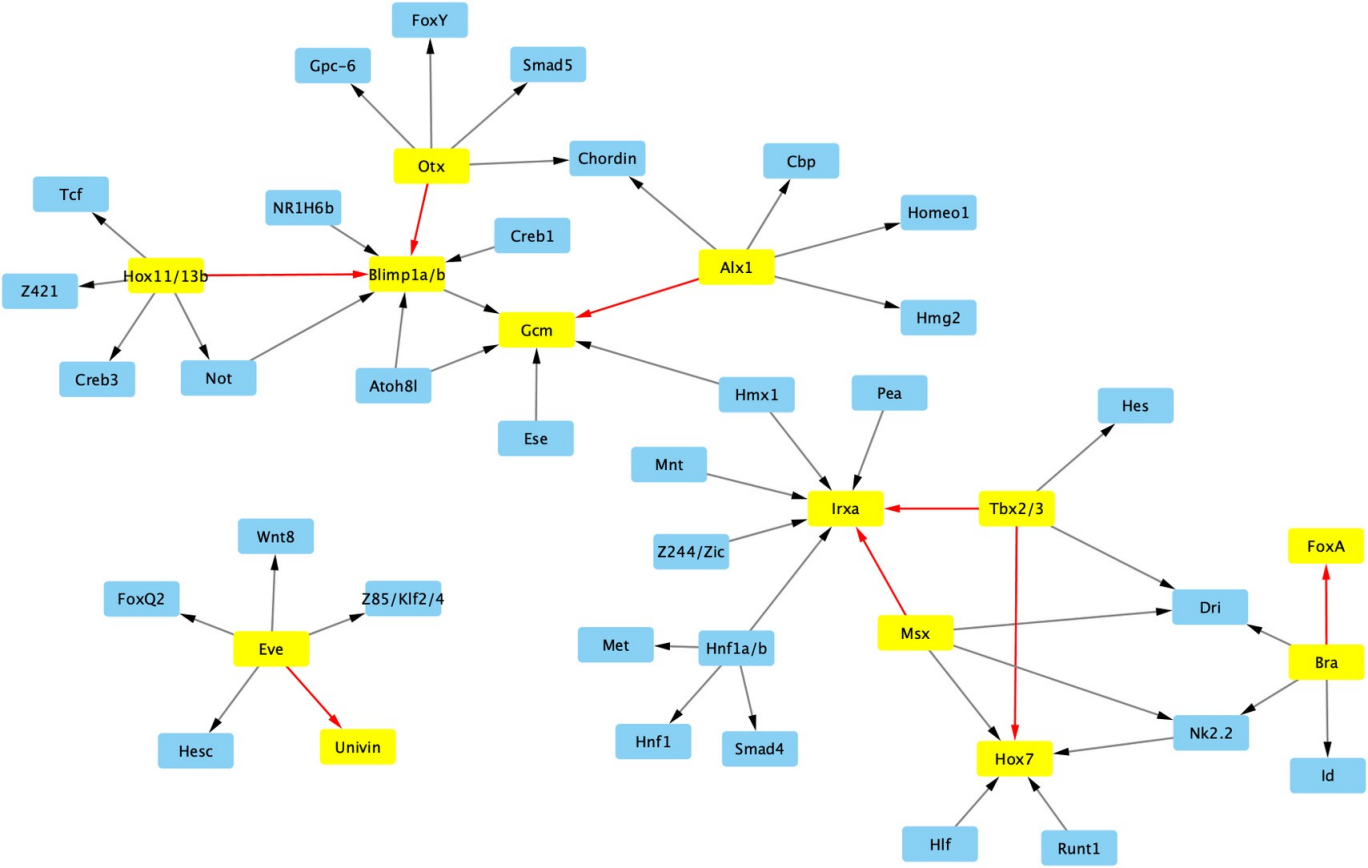
Example PEAK predicted interactions. An interactome visualization of a subset of PEAK-predicted interactions within the top-5 predictions (highest confidence scores) for each gene. Both known interactions (yellow gene boxes and red arrows) and new predicted interactions (blue gene boxes and black arrows) are included among these predictions.

We applied the PEAK GRN prediction algorithm on the filtered gene set of 504 DEGs as determined by NOISeq that are also identified by the GO filter. We specified to PEAK the set of 254 transcription factors (TFs) used during embryonic development, according to Materna et al. [22] and Tu et al. [12] (S2 File). We used the filtered gene set and TF specification file as inputs into PEAK and set all the other parameters as default except for “Repeat,” which was set to 1 (S3 File). The output from PEAK contained 14,802 predicted interactions in total (S4 File). Among the set of predictions from PEAK representing the top 5 hits for each gene, there are both known interactions and new predictions (Fig 2).

To evaluate the performance of PEAK, we repeated the PEAK analysis with data relating only to genes present in the publicly available sea urchin GRNs, using the same procedure and parameters as with the set of all DGE genes that fit the GO filter. The set of known GRN interactions is defined as the ground truth. For our analysis, we used three measures to perform the evaluation. *Sensitivity* (or true positive rate) is a common assessment measure for classification problems [23]. Sensitivity represents the proportion of our predicted gene interactions that hit the corresponding ground truth GRN interaction list, indicating the percentage of gene interactions that are correctly identified by the PEAK algorithm among the total known ones. Another common metric used alongside sensitivity is specificity. However, specificity is only applicable when the ground truth is fully known. We have not used the specificity measure here because the GRN models are missing an unknown number of undiscovered gene interactions, and the solidity of the data underlying the connections the GRN model is variable. Additionally, there are other complexities inherent in developmental gene regulation that present limitations, including transient TF binding, multiple upstream regulators, and indirect interactions dependent on signaling [24]. The second measure we used was the set of *new predictions*, which designated new gene interactions predicted by the algorithm that were not yet known in the ground truth GRN. Finally, the *miss rate* (or false negative rate), another common metric, represented the proportion of known gene interactions not discovered by the algorithm.

We evaluated the ground truth from the ectoderm GRN and the endomesoderm GRN separately, and compared the results, as summarized in Table 2. For the known ectoderm GRN, there are 37 genes out of 39 that had a clear match in the transcriptome RNAseq data and were designated as a DEGs. When gene expression data from the transcriptome RNAseq dataset were used as input for PEAK, the algorithm successfully predicted 54 of 76 gene-to-gene interactions present in the ground truth GRN, yielding 71.05% sensitivity (Table 2). PEAK failed to predict 22 known connections but provided 530 possible new ones. The sea urchin endomesoderm GRN is currently a larger network model, with 55 genes and 121 edges. We found 53 of those 55 genes had a clear match in the transcriptome RNAseq data and were designated as a DEGs. When TFs were specified, 74 of the 115 connections were correctly predicted for a sensitivity of 64.53% (Table 2).

**Table 2:** Statistic PEAK result.

Dataset	Transcriptome RNAseq data		Nanostring data	
Ground Truth GRN (GT)	Ectoderm GRN	Endomesoderm GRN	Ectoderm GRN	Endomesoderm GRN
True predictions (TP)	54	74	44	62
Sensitivity	71.05%	64.53%	72.13%	81.58%
Miss rate	28.95%	35.65%	27.87%	18.42%
New predicted edges	530	1103	508	788
Total predictions	584	1177	552	850

Statistic result for transcriptome RNAseq data and the Nanostring data compared with each ground truth GRN. Sensitivity represents the proportion of our predicted gene interactions that hit the corresponding ground truth GRNs. Miss rate represents the proportion of known gene interactions not discovered by the algorithm. New predicted edges are predicted interactions that are not part of the ground truth GRN. Total predictions include both new predicted edges and predictions that are known.

To achieve a higher sensitivity, we considered what limitations might be present in the initial approach. A limitation of the transcriptome RNAseq data when applied to the ground truth GRN is that only 10 timepoints were sampled in total and, of those, only 5 timepoints covered the period of time during early development (0-30hrs) which the ground truth GRN models describe. Therefore, we sought a second data set with more timepoints at closer sampling intervals. For the second data set, we chose a high-density embryonic data set where 335 genes critical to early development were sampled every 2 hours for 72 hours in duplicate, and gene expression was quantified using Nanostring technology, an alternative to RNAseq that requires a probe set [25]. Because there were genes in the ground truth GRNs that did not have a match in the Nanostring probe set, we only retained the genes that were sampled in the Nanostring data set. For the ectoderm data, there was a clear match for 30 of 39 genes, and the corresponding number of gene interactions in the ground truth ectoderm GRN that are therefore possible to predict was 61. Comparing PEAK predictions to the corresponding ground truth table resulted in 44 successful gene-to-gene interactions predicted out of 61, yielding a sensitivity of 72.13% (Table 2). Thus, for the ectoderm GRN, we found that PEAK gave similar prediction results for the Nanostring data and the RNAseq data. For the endomesoderm data, 38 genes were present in both the ground truth GRN and the Nanostring data set, which corresponded to 76 known GRN interactions. Comparing PEAK predictions to the corresponding ground truth table resulted in a total of 62 successful gene-to-gene interactions predicted out of 76, for a sensitivity of 81.58% (Table 2). The improved performance of PEAK on the endomesoderm GRN using the Nanostring data set as compared to the RNAseq data was likely due to the additional timepoints in the Nanostring data set.

**Fig 3:**
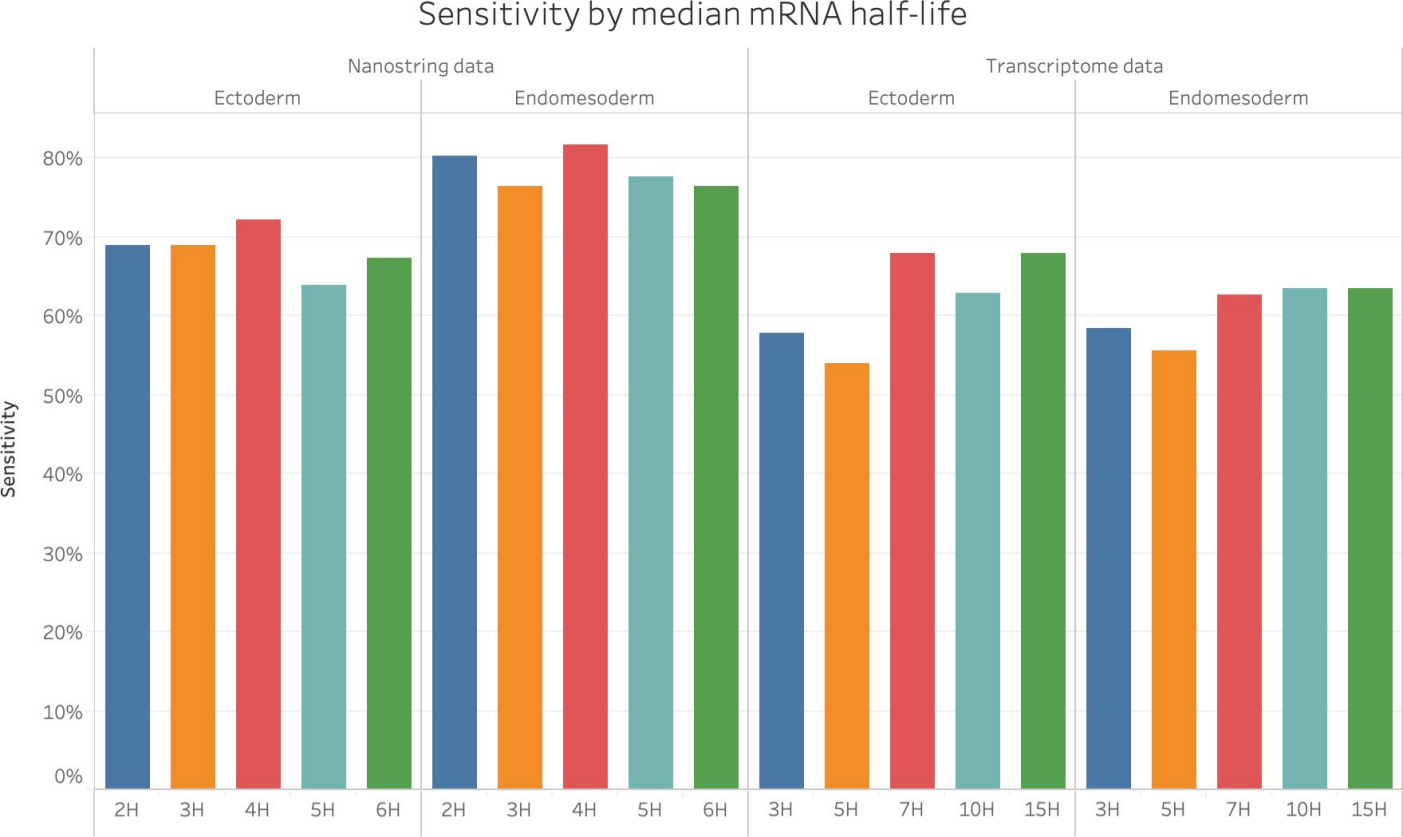
Half-life value evaluation. Sensitivity as a measure of accuracy for the prediction of ectoderm and endomesoderm gene regulatory relations calculated with 5 different median mRNA half-life settings. For the Transcriptomic RNAseq data, 3hrs, 5hrs, 7hrs, 10hrs, 15hrs were tested. For the Nanostring data, 2hrs, 3hrs, 4hrs, 5hrs, 6hrs was tested.

We further tested the impact of adjusting the parameter describing transcript turnover due to maternal and zygotic degradation mechanisms in terms of transcript half-life on the prediction results. Recent estimates of transcript turnover in sea urchin are in the range of 6 to 9 hours [26]. We explored the performance of PEAK for a range of median half-life times from 3 hours to 15 hours (Fig 3). In general, the sensitivity of the predicted results did not have any obvious increase or decrease trend with the increase of half-life. We obtained the highest sensitivity on the transcriptome RNAseq data when the half-life was set to 7 hours. For the Nanostring data set, which was sampled every 2 hours, we used even-numbered half-life values. We obtained the highest sensitivity on the Nanostring data when the half-life was set to 4 hours.

### Further investigation of predictions

To assess the quality of our predicted network connections, we looked for corroborating data based on existing genome-wide binding assays. Among our top 50 predictions from PEAK, there were 5 predicted targets for aristaless-like homeobox (*Alx1*), a well-known transcription factor involved in *S*. *purpuratus* skeletogenesis [27]. One of the PEAK predictions for a target of Alx1 is glial cells missing (*Gcm*), which represents a known target of Alx1 [28]. The next 4 predicted Alx1 targets with the highest confidence scores are unknown connections. Genome-wide Alx1 ChIPseq has recently been performed and published [29]. We analyzed Alx1 ChIPseq peaks marking putative binding sites within 20kb of the 4 unknown predicted targets and, for 3 of the 4 targets, we found Alx1 peaks that overlapped with open chromatin as marked by ATAC-seq and DNase-seq (S5 File) [30]. We extended the analysis to all 27 of the predicted targets of Alx1 returned by PEAK, ordered by confidence scores, and found peaks within 20KB for additional predicted targets, with more variance in the statistical relevance of the peak calls and more variance in the degree of overlap with markers of open chromatin as the confidence score predicted by PEAK diminished (S5 File).

Spatial co-expression can also be used to evaluate the likelihood of gene interaction. The spatial expression of many developmentally important genes has been examined in *S*. *purpuratus*. Echinobase organizes known spatial expression profiles using a matrix of distinct cell types at each time point during development. We made use of these published profiles to evaluate spatial co-expression for our top 50 predictions (S6 File) and compared them to the co-expression of our bottom 50 predictions (S7 File). Only 5 of the top 50 predicted interactions were between genes with non-overlapping expression patterns. All 5 of those interactions that occurred between genes with mismatched spatial expression patterns involved regulation by a TF with known repressor activity in sea urchin (Eve, Alx1) or other organisms (Hmx, Runt1) [31, 32] where non-overlapping expression would be expected or conceivable. We were not able to identify any of the top 50 predicted interactions as definite false positives by spatial expression analysis alone. Among the top 50 predicted interactions, 21 interactions displayed overlapping spatial expression patterns. For the remaining 24 interactions, or almost 50%, either 1 or both genes did not have spatial expression data readily available in the database. The large number of predicted interactors without spatial expression information indicates that even in an extensively studied model organism there remains a significant number of unknown and untested genes. The bottom 50 predictions, as sorted by PEAK confidence scores, contained 2 interactions with non-overlapping expression patterns, 46 interactions without known spatial information for 1 or both genes and 2 interactions with SPU IDs that did not match the database. Comparing the top 50 predicted interactions with the bottom 50 predicted interactions revealed that there were clearly more interactions with spatial co-expression in the top 50 (42% versus 0%).

## Discussion

Computational prediction methods of GRN components have been explored in many organisms. In bacteria, for example, researchers used an integrative method to predict a GRN in *B*. *subtilis* with a large amount of input data [33]. Specifically, the *B*. *subtilis* study used more than 600 gene expression experiments and incorporated prior knowledge from the ground truth network to improve accuracy. The sensitivity of their GRN prediction is 74% and they predicted 2,258 new regulatory interactions. The scale of experiments used in the *B*. *subtilis* study by Arrieta-Ortiz et al. is difficult to achieve in multicellular organisms [33]. Also, as with most other machine learning applications, the more prior knowledge available to train the algorithm, the better predictions one can expect the algorithm to produce. The approach using PEAK described in this manuscript achieved a similar level of sensitivity while meeting three additional challenges. First, there is an extended challenge when working with a multicellular animal with many different cell types expressing different transcriptional programs. Second, there is a challenge of working with the highly dynamic embryonic developmental program in which new cell types are created and transcriptional programs change rapidly. Finally, we limited input to gene expression data, instead of adding additional prior information, to see what can be achieved with expression data alone. Our maximum result of 81.58% sensitivity allowed us to conclude that a relatively high level of sensitivity can be achieved from gene expression data alone, even when working with complex developmental GRNs. Although a successful result was achieved, there were limitations to the application of *S*. *purpuratus* data in evaluating PEAK on a developmental GRN. The RNAseq data set we used, although still referenced on individual gene pages in Echinobase as the source of expression data, has low temporal resolution during the period of embryogenesis covered by the GRN. We addressed this limitation by using the Nanostring gene expression data set which had a much higher temporal resolution, but the Nanostring data set did not include every gene in the GRN model. The ideal gene expression data set would have both high temporal resolution and complete transcriptomic coverage.

A few developmental model systems like *Drosophila melanogaster* have extremely large research communities producing extensive prior knowledge data sets in the form of ChIP assays, protein-protein interaction databases, functional gene annotation, extensive tissue expression information, TF-binding sites, and known regulatory interactions. In this case, the richness of prior knowledge was harnessed in a combination of supervised and unsupervised machine learning approaches to produce novel network interaction predictions [34]. Despite the wealth of omics data, only 204 network edges were available in *D*. *melanogaster* to train their supervised network, pointing to the need in all systems to continue rigorously verifying gene network connections. Uncovering universal regulatory mechanisms will require GRN knowledge from a wide variety of model species. The first omics step in many research programs establishing new model species is often the creation of a transcriptome, which inherently creates a quality source of gene expression data [17, 35–39]. Collectively, there is a glut of sequencing data that researchers are eager to use to understand the network of regulatory interactions that govern building the animal body plan. The investigation of the way changes in the GRN controlling embryonic development have shaped evolution is a particularly active research question and one that can benefit directly from using GRN inference approaches to create model networks. Our approach of using only gene expression data as input to generate regulatory interaction predictions will be particularly useful to researchers who are rapidly establishing new model systems.

One goal of GRN inference is to narrow the search space to a subset of promising interactions to be further studied and validated experimentally. With approximately 21,000 to 23,000 gene models predicted in the sea urchin, there are up to ~540 million possible gene-to-gene interactions. Using time series gene expression data as input into the PEAK prediction algorithm for the 547 transcripts most likely to be a part of the regulatory program, we generated 14,802 predicted interactions that can be ordered by confidence or searched for specific regulators or target genes. One caveat to our approach is the differential equations that PEAK is based on cannot predict self-interactions in which genes turn on or off their own expression. These self-interactions are known to be important to the precision and robustness of regulatory programs. Self-activation is known to give rise to bistability which is harder to model and train computationally; thus, most methods for GRN inference do not model self-regulation [40, 41]. While it is always advantageous to have an algorithm that can predict the greatest number of biologically meaningful interactions, experimental design can take this limitation into account and experimentally check for self-regulation when investigating the targets of regulatory genes. Another caveat to our approach is that while PEAK does return a direction of interaction (positive/enhancing or negative/repressing) and the direction is included in our list of all predictions (S4 File), we did not include direction in our analysis in order to compare our results to previously published methods. Without spatial expression in the input, it is challenging for the algorithm to always correctly predict the direction of the interaction. Nevertheless, we found that within the top 5 predictions for each target gene, the correct direction of interaction was predicted 75% of the time.

Although a set of putative TFs can be generated from any transcriptome using BLAST-to-GO, we also tested the ability of PEAK to make predictions using our data sets without specifying the set of TFs [17, 42]. Surprisingly, the sensitivity was not dramatically different with or without a specified TF list. Since the performance of PEAK was not overtly affected when the list of TF was not specified to the algorithm, a lack of complete functional gene annotations is not a hindrance to using our approach. It is also possible to specify the transcript half-life to PEAK, yet it is challenging to determine the optimal average half-life to use considering the differences between maternal and zygotic transcripts and individual variation. Our current recommendation is to use a range of half-life values to find the best fit to the data empirically. Future directions for improvement to the algorithm could include a more customizable way to vary half-life values for different transcript types.

While it is difficult to assess the rate of false positives in our predictions without targeted experiments, we investigated of the quality of our results using publicly available ChIPseq data sets and spatial expression data. Examining Alx ChIPseq data for our top predictions involving Alx as the regulator provided encouraging feedback. While the peaks would need to be validated with cis-regulatory analysis to confirm functionality, the similarity of number and position of peaks to those of known targets of Alx1 indicates that at least some of the peaks may represent functional binding sites and true targets. Spatial co-expression analysis alone was not able to identify any definitive false positives in the top 50 interactions. The five interactions with known mis-matched spatial expression profiles all involved a known repressor as the regulator. Furthermore, nearly half of the top 50 predictions were for interactions between genes where one or both genes did not have spatial expression data in Echinobase. Spatial expression analysis will be crucial to confirming any predictions and ideally could be included as input in the form of a single-cell RNAseq data set.

We found that using gene expression data as the sole input for machine learning GRN predictions was sufficient to generate predictions that match known regulatory interactions when using the PEAK program. Our approach also generated new possible gene-to-gene interactions that are not currently described. The new predictions from our data set will serve as a resource for the sea urchin community; a relatively small, but influential group, in the areas of *cis*‐regulatory biology and developmental regulatory networks. Our future research will include applying PEAK to emerging model species where transcriptomic gene expression data exists in order to generate predictions for building and testing initial GRN models. Our method is broadly applicable and accessible to any organism with gene expression data. Although no prior knowledge is required, PEAK can accept many forms of prior knowledge to improve the quality of predictions [10].

## Materials and methods

### Data sets

Two gene expression data sets were obtained to use as input into the PEAK algorithm. The first data set comes from the sea urchin transcriptome project [12] where 10 embryonic timepoints (labeled with time in units of hours-post-fertilization (hpf)) were assayed for transcript expression by RNAseq. The sequenced transcripts in the transcriptome data set derived from cDNA collected from: (1) the unfertilized egg, 0 hpf; (2) cleavage stage, 10 hpf; (3) hatched blastula stage, 18 hpf; (4) mesenchyme blastula, 24 hpf; (5) the early gastrula, 30 hpf; (6) mid-gastrula stage, 40 hpf; (7) late-gastrula stage, 48 hpf; (8) prism stage, 56 hpf; (9) late prism stage, 64 hpf (10) the pluteus stage, 72 hpf. All embryonic samples were obtained from a single male and female mating pair, except the 24hr sample which was done separately as a pilot experiment. Only a single replicate was sequenced for each time point, presumably due to limitations of the amount of material that can be obtained from a single spawning event and the decision not to introduce biological variation due to individual differences if multiple urchins were used. Each sample generated approximately 36.5M reads, of which 79% mapped to the *S*. *purpuratus* genome v3. Gene models were built by Cufflinks, and, after quality filtering, 21,092 transcript models were defined and assigned an 8-digit WHL ID number beginning with “22.” These models have been incorporated into the annotated sea urchin gene database and assigned to previously established “SPU ID” numbers. Initially, values of expression for each gene model were expressed in FPKMs (Fragments Per Kilobase of transcript per Million mapped reads) as determined by Cufflinks. The gene expression values were then converted into transcripts per embryo by the authors [12]. We obtained the full data set with gene models identified by WHL ID and SPU ID and expression values for each timepoint in transcripts per embryo. We converted the expression values into RPKMs (Reads Per Kilobase of transcript per Million mapped reads) for our analysis.

The existing GRN models for sea urchin development cover the time period from 0 to 30 hpf. Of the 10 time points sampled in the transcriptome data set from Tu et al. only 5 are represented in the range of 0-30hpf [12]. Therefore, we decided to also use data from a high density time series containing 34 time points, sampled every 2 hours over the first 72 hours of development. The data set from Roberto Feuda uses Nanostring technology, which is often used as a gold standard for absolute quantitation due to the fact that it measures RNA directly as opposed to using an enzymatic reaction [43]. Even the Tu et al. transcriptome data set was validated using independent Nanostring quantitation [18, 22]. A key difference between RNAseq and Nanostring is that Nanostring will only produce data for specific known gene models for which probes were designed, whereas RNAseq surveys the entire transcriptome. The sea urchin Nanostring data set we used queried 335 regulatory gene products, including transcription factors and other modulators of gene expression. This gene set overlaps nicely with the gene set present in the sea urchin ectoderm and endomesoderm GRN models. Specifically, 62 genes in total overlap between the Nanostring probe set and the ground truth GRN genes. The overlap includes 29 of 38 genes overlapping with the ectoderm GRN and 38 of 54 genes overlapping with the endomesoderm GRN. There are 6 genes in the Nanostring probe set that appear in both the ectoderm and endomesoderm GRNs (namely, *unc4*.*1*, *not*, *foxA*, *eve*, *myc*, and *bra*). The normalized RNA counts produced by the Nanostring’s Ncounter were used directly in our analysis.

### Sea urchin GRN models

The most recently updated versions of the complete *S*. *purpuratus* GRNs for endomesoderm and ectoderm development are hosted by the Institute for Systems Biology and can be accessed online using the web application BioTapestry Interactive Network Viewer [15]. The endomesoderm GRN can be found at http://grns.biotapestry.org/SpEndomes/, and the ectoderm GRN can be found at http://grns.biotapestry.org/SpEcto/. These two GRN models were built by a collaboration of sea urchin labs over the last thirty-some years. Each regulatory interaction is depicted as a directional line connecting two gene nodes, and each interaction is supported by experimental evidence, which can be accessed in the BioTapestry viewer directly. We obtained lists of the genes present in each network and a list of every gene-to-gene interaction present in the current version of the models from the BioTapestry director William Longabaugh. There are 39 genes represented in the ectoderm GRN and 55 genes represented in the endomesoderm GRN. The interaction list we obtained includes direct interactions and indirect interactions that are driven by signaling molecule intermediates. Interactions derived from signaling intermediates were not used in our comparison list. We also removed interactions where a gene regulates its own expression because the PEAK algorithm is not designed to be able to predict this type of interaction, due to the mathematical equations that it is built on. The final list used in our analysis contained 82 unique gene-to-gene interactions present in the ectoderm GRN and 121 unique gene-to-gene interactions present in the endoderm GRN (S8 File). These unique interactions made up our ground truth GRN models, which were used for comparison to the interactions predicted by the PEAK algorithm. The number of genes and connections included in our analysis when requiring a match between gene expression data set and corresponding ground truth network are described in Table 3 and listed as separate tabs in S8 File.

**Table 3:** Summary of data sets.

Data set	Transcriptome RNAseq data set	Transcriptome RNAseq data set	Nanostring data set	Nanostring data set
Ground Truth GRN (GT)	Ectoderm GRN	Endomesoderm GRN	Ectoderm GRN	Endomesoderm GRN
Method	RNAseq	RNAseq	Nanostring	Nanostring
Timepoints (T)	10	10	16	16
Genes (N)	37	53	30	38
Edges in GT	76	115	61	76

Summary of data sets used in the evaluation of PEAK’s predictions. For each data set, we only used the genes that appear both in the gene expression data and the ground truth GRN data. We also only used the timepoints in the Nanostring data set corresponding to the time period covered by the GRN (0-30hrs), which corresponded to 16 timepoints, sampled every 2 hours.

### Preprocessing and differential gene expression determination

The RNAseq data set was constructed with only one biological replica. Multiple methods have been developed to perform differential gene expression analysis on RNAseq data when only a single biological replica is available. These methods include: NOISeq [19], based on the multinomial distribution; GFold [21], based on the posterior distribution of log fold change; and EdgeR [20], based on the negative binomial (NB) distribution. To discover quantitative changes in expression levels between experimental time points, we first applied NOISeq, GFold, and EdgeR to determine the set of differentially expressed genes for further analysis.

For NOISeq, we normalized the data by RPKM (Reads Per Kilobase of transcript per Million mapped reads), which takes into account that more sequencing reads are generated from longer transcripts. The length of each transcript was obtained from the sea urchin database, Echinobase (https://www.echinobase.org) [44]. We omitted genes that had no record in the gene database. We set the simulation parameters as recommended in the NOISeq handbook, where the percentage (*pnr*) of the sequencing depth is *pnr* = 0.2, the number of samples to be simulated (*nss*) for each condition is *nss* = 5 and a small variability (*v*) is *v* = 0.02. We selected the differentially expressed genes with the higher NOISeq probabilities based on our chosen thresholds λ_1_ = 0.9, λ_2_ = 0.85. For GFold, we set the thresholds for the GFold value to λ_3_ = ∓1, λ_4_ = ∓ 1.5, since the GFold value is similar to the log2 fold change that is reliably used to select differentially expressed genes. For EdgeR, we set the log2 fold change (*log2fc*) cutoff as 2 and the edgeR dispersion as 0.01.

We used the gene database at Echinobase [44] to map all WHL IDs to SPU IDs to ensure that the IDs we analyzed before and after are consistent and unique.

### Computational GRN prediction

PEAK was used for our computational GRN predictions [10]. PEAK is a previously tested and published algorithm that relies on differential equations, context likelihood of relatedness (CLR), and Elastic Net. The particular mathematics underlying PEAK limit the computational load to enable gene network inference to be an efficiently solvable problem. For each target gene, PEAK builds a model using differential equations to predict the likelihood of being regulated by each transcription factor in the dataset. PEAK initially uses CLR to filter out unlikely TFs, then solves a regularized linear regression model (Elastic Net) to further optimize the predicted TFs and find their confidence score.

PEAK can be accessed as a front-end web application that is friendly to biologists, available here: http://detangle.cs.vt.edu/. For each experiment, the corresponding gene expression table is uploaded as input along with the corresponding list of transcription factors and metadata about the experiments. We also experimentally tested different half-life values. PEAK returns predicted interactions for each gene that scores above the confidence threshold set by the ‘PEAK value’. Each target gene has up to 30 ranked regulator (TF) predictions. Predictions are marked positive or negative when representing an enhancing interaction or a repressive interaction, respectively. Each interaction is assigned a confidence score, allowing users to sort the interactions with the highest confidence or the top 5 or top 10 predicted interactions for each gene.

### Assessment of Alx1 ChIPseq peaks

The Integrative Genomics Viewer (IGV) was used to visualize ChIPseq data for the Alx1 transcription factor, which had several novel predicted targets among our top predictions [45, 46]. The *S*. *purpuratus* genome version 3.1 was used as the reference genome because the newer version 5 release is still undergoing annotation and was more difficult to map existing datasets that organize genes by SPU_ID and transcripts by WHL IDs. Indexed transcript models were first loaded into IGV [12]. Alx1 ChIPseq data was obtained from a recent publication [29], and peak calls for three *P*-value cutoffs, p<0.1, p<0.05, and p<0.005 were loaded as individual tracks into IGV. To mark open chromatin, ATAC-seq and DNase-seq data from 24-hr *S*. *purpuratus* embryos were also loaded into the IGV [30]. For each of the 27 predicted target genes for Alx1, we analyzed a window of +/- 20kB from the endpoints of the gene model and counted the number of peaks called in that region at each of the *P*-value cutoffs. Images from IGV for windows containing the genes *chordin*, *cpb*, *homeo1*, and *hmg2*, and a table of the peak counts are included as S7 File.

### Spatial co-expression analysis

Spatial co-expression analysis was performed using published spatial expression information available within either the legacy Echinobase website or underlying the Biotapestry GRN model, which stores data for each gene and data supporting each interaction. We visualized the spatial expression information for each gene in a predicted interaction and marked the overlap with a distinct color pattern. The matrix of spatial expression catalogs tissue-specific expression over the first 30 hours of development. Using this analysis, we compared co-expression for our top 50 interactions to our bottom 50 interactions and marked each prediction as “overlapping,” “non-overlapping,” or “missing expression.” An interaction was marked as missing expression if either gene in an interaction was missing spatial expression information in the database. The results are all part of S8 File.

## Supporting information

S1 File Gene lists after GO and DEG filtering. This excel sheet contains 2 tabs. Sheet ‘GeneOntology_filter’ contains 1038 genes determined to be regulatory in nature by Gene Ontology, annotated by their SPU_ID and sheet ‘GO_filtered_DEGs’ contains 544 transcripts that passed both the GO filter and DEG filter.
(XLSX)

S2 File Transcription factors list. The 254 Transcription Factors (TFs) used during embryonic development according to a compilation of genes specified as TFs by Materna et al. [22] and genes specified as TFs according to the custom GO annotation by Tu et al. [12].
(XLSX)

S3 File Input files for PEAK prediction. This zipped file contains the metadata file, gene expression file and the Transcription Factor list used for PEAK prediction.
(ZIP)

S4 File Prediction result of PEAK. The output file of the PEAK prediction. Each row represents a predicted gene-to-gene interaction. The first column is the predicted regulating TF or Gene1 and the second column is the target gene or Gene 2. The third column is the confidence score of the prediction. Each target gene has up to 30 ranked regulator predictions. Predictions are marked with a positive or negative confidence score when representing an enhancing interaction or a repressive interaction, respectively. The genes are annotated by SPU_ID.
(CSV)

S5 File Analysis of Alx1 ChIPseq, ATAC-seq and DNAseq data for predicted Alx1 targets. Mapped Alx1 ChIPseq, ATAC-seq and DNAseq data onto sea urchin genome visualized in the region of 4 novel PEAK-predicted target genes for Alx1.
(PDF)

S6 File Spatial coexpression analysis for the top-50 predictions. The spatial expression comparison it its entirely for the Top-50 interactions, as sorted by absolute PEAK confidence scores. Starts with a summary table. For each interaction a separate page displays the spatial expression for each gene in each interaction presented as a color-coded co-expression matrix.
(DOCX)

S7 File Spatial coexpression analysis for the bottom-50 predictions. The spatial expression comparison it its entirely for the Bottom-50 interactions, as sorted by absolute PEAK confidence scores. Starts with a summary table. For each interaction a separate page displays the spatial expression for each gene in each interaction presented as a color-coded co-expression matrix.
(DOCX)

S8 File GRN ground truth interactions for each experimental analysis. This file has 6 tabs representing the ground truth GRN interactions used for comparison to each gene expression data set. Tab 1 is the ground truth ectoderm GRN interactions used for comparison to PEAK output from the RNAseq data. Tab 2 is the ground truth endoderm GRN interactions used for comparison to PEAK output from the RNAseq data. Tab 3 is the ground truth ectoderm GRN interactions used for comparison to PEAK output from the Nanostring data. Tab 4 is the ground truth endoderm GRN interactions used for comparison to PEAK output from the Nanostring data. The last two tabs show a direct comparison of which interactions were able to be used for analysis using the RNAseq dataset or the Nanostring dataset.
(XLSX)
