# Supplementary material for: Developmental gene regulatory network connections predicted by machine learning from gene expression data alone
Source: PLoS One. 2021 Dec 28;16(12):e0261926. doi: 10.1371/journal.pone.0261926 (PMC8714117; doi:10.1371/journal.pone.0261926)
Supplement: S5 File — Mapped Alx1 ChIPseq, ATAC-seq and DNAseq data onto sea urchin genome visualized in the region of 4 novel PEAK-predicted target genes for Alx1. (PDF) [file pone.0261926.s005.pdf]

Gene Name: Chordin
SPU ID: SPU_004983
WHL: WHL22.124093

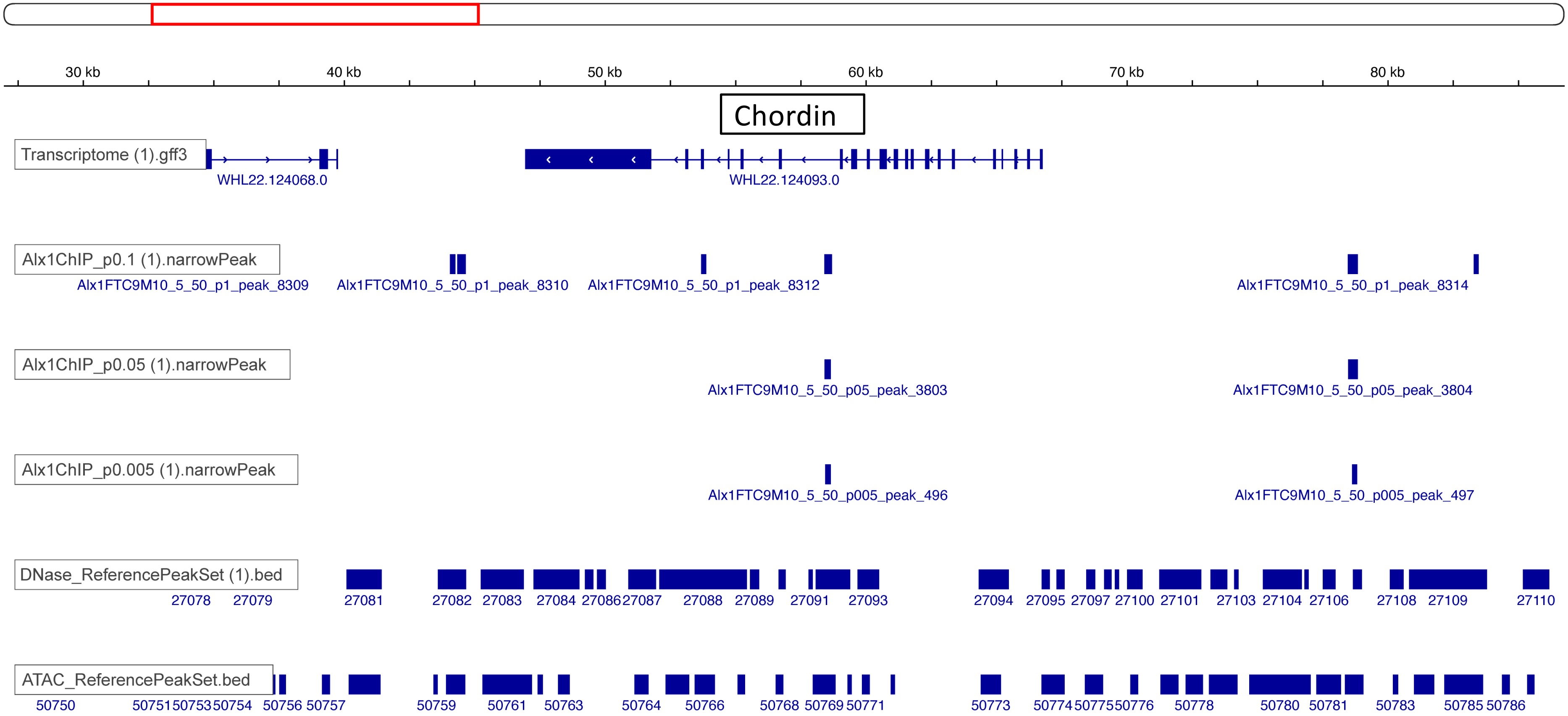

Gene Name: Cpb

SPU ID: SPU_019024

WHL: WHL22.581111

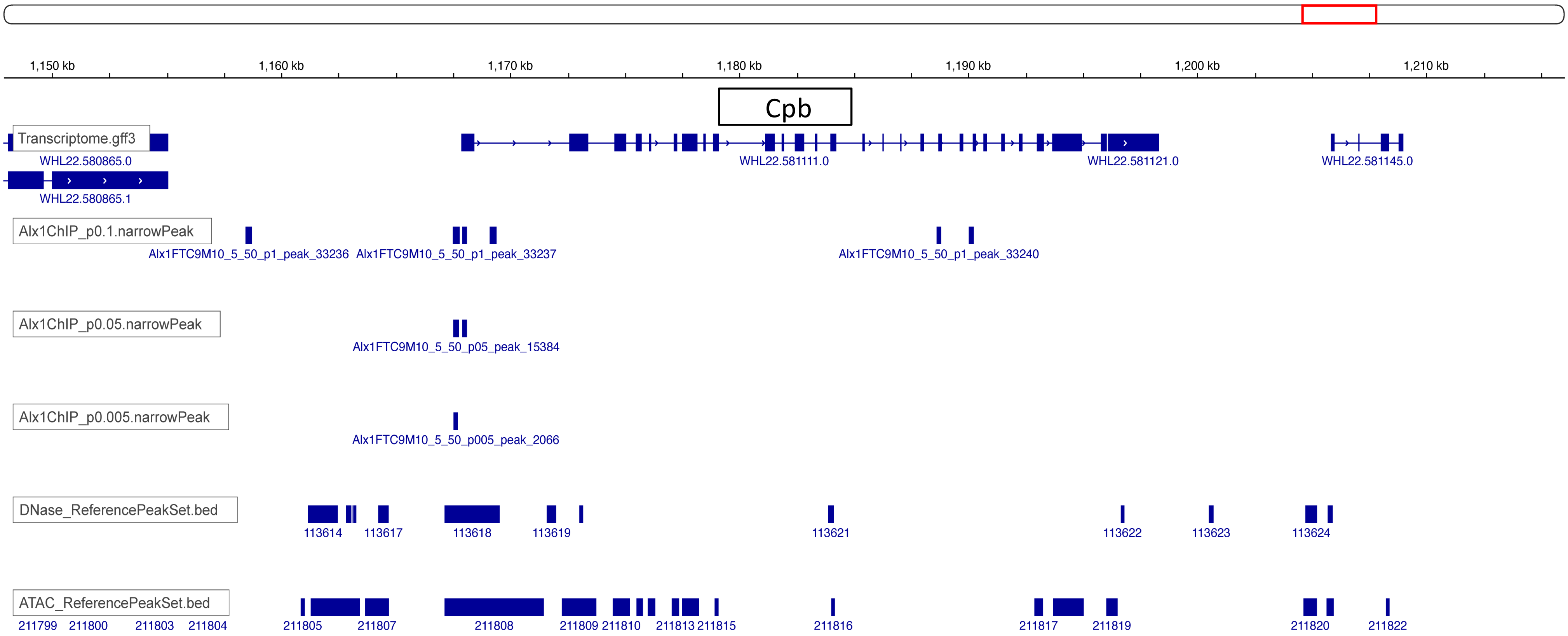

Gene Name: Homeo1
SPU ID: SPU_018056
WHL: WHL22.290173

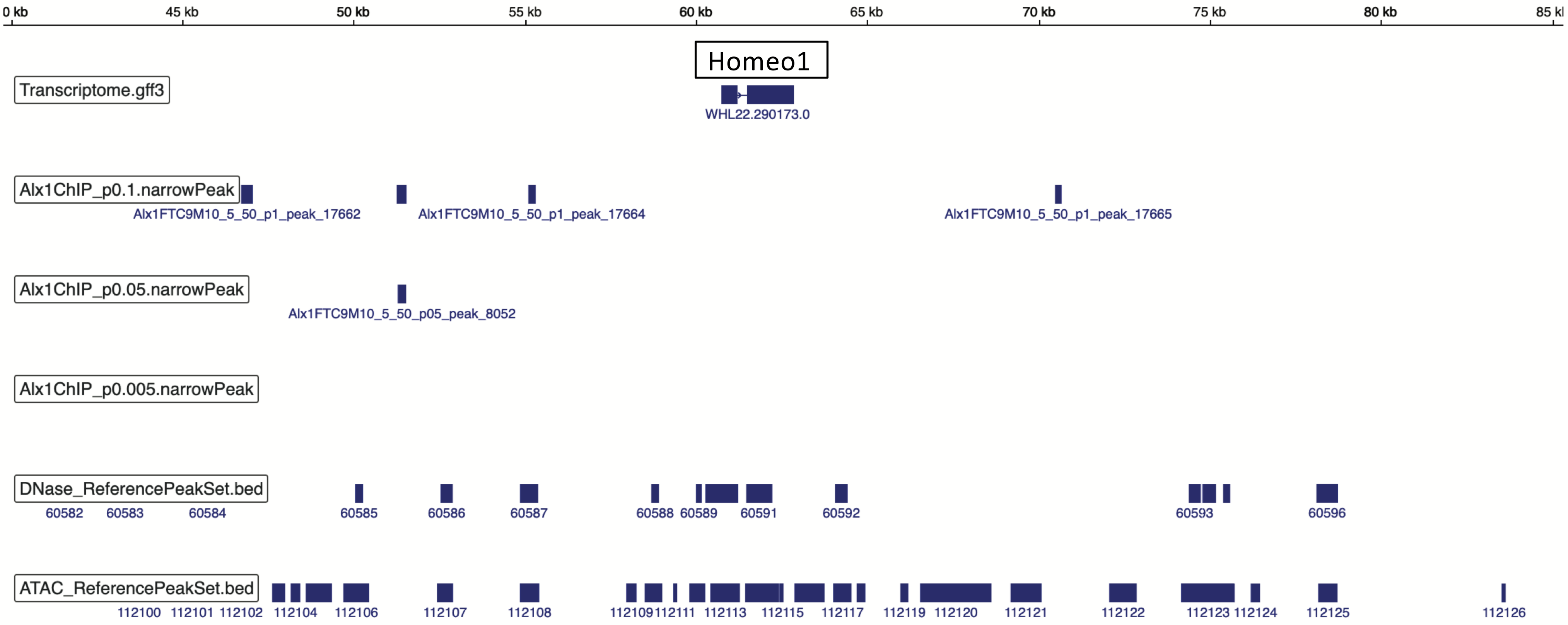

Gene Name: Hmg1
SPU ID: SPU_005572
WHL: n/a

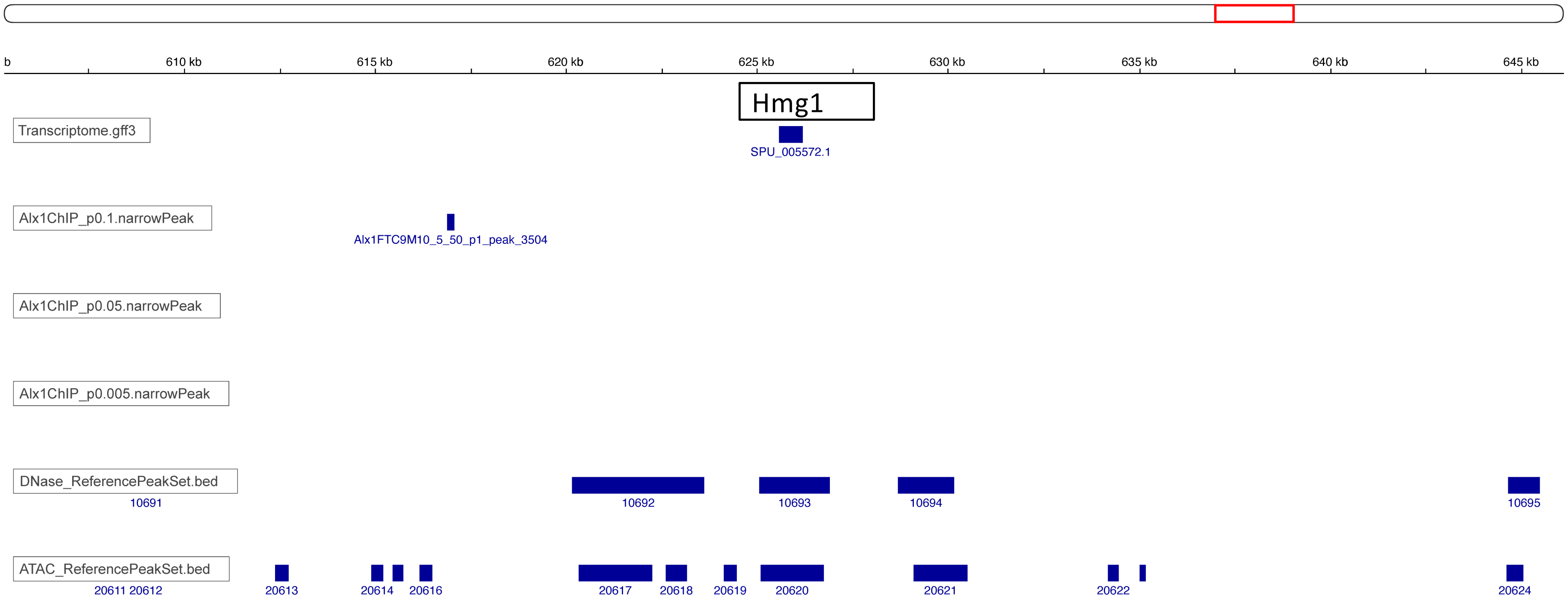

SPU ID	Gene Name	WHL	Genome v3.1 Location	# of peaks +/- 20kb			
				**p0.1**	**p0.05**	**p0.01**	**p0.005**
SPU_018056	Sp-Homeo1	WHL22.290173.0	Scaffold2250:60748-62866	-	4	1	-
SPU_006462	Sp-Gcm	WHL22.54333.0	Scaffold118:115352-123123	-	3	1	-
SPU_005572	Sp-Hmg2	N/A	Scaffold116:625586-626200	1	-	-	-
SPU_004983	Sp-Chordin	WHL22.124093.0	Scaffold1440:46956-66808	8	2	2	2
SPU_019024	Sp-cbp	WHL22.581111.0	Scaffold50:1167867-1196039	7	2	1	1
SPU_002603	SoxC	WHL22.622787.0	Scaffold622:183497-186988	9	4	1	1
SPU_018483	Sp-Erg	WHL22.552472.0	Scaffold499:88697-401319	39	26	11	7
SPU_003704	Sp-Tcf/Lef	WHL22.106048.0	Scaffold137:50694-189633	6	3	-	-
SPU_019384	Sp-Hinfp	WHL22.143511.0	Scaffold1527:34208-42861	5	3	3	2
SPU_004807	Sp-Z32	WHL22.520622.1	Scaffold45:307479-32125	7	3	1	-
SPU_022916	Sp-Fzd5/8	WHL22.42488.0	Scaffold113:922295-927951	3	1	-	-
SPU_017635	Sp-Pax4L	WHL22.82981.0	Scaffold1288:218374-233903	6	3	-	-
SPU_022049	Sp-Msx	WHL22.119881.0	Scaffold1422:38289-52620	6	2	-	-
SPU_004806	Sp-Z197	WHL22.520690.1	Scaffold45:321715-332323	5	2	-	-
SPU_022163	Sp-Max	WHL22.335395.0	Scaffold256:34515-54435	3	1	-	-
SPU_017348	Sp-Atbf1	WHL22.369566.0	Scaffold28:50791-169799	21	13	6	4
SPU_004217	Sp-SoxD	WHL22.118185.2	Scaffold141:231385-257442	7	2	1	-
SPU_004746	Sp-Fgfr2_1	WHL22.147868.0	Scaffold1548:272736-335018	16	14	1	1
SPU_003548	Sp-E78a	WHL22.454662.1	Scaffold361:161557-166196	4	3	1	-
SPU_014643	Sp-Prdm10L	WHL22.568415.0	Scaffold521:146357-161788	3	1	-	-
SPU_018355	Sp-Nr2dbd	WHL22.757746.0	Scaffold95:1080798-1135079	3	1	-	-
SPU_003490	znf131l	WHL22.296613.0	Scaffold2282:38122-74823	4	3	-	-
SPU_017441	znf845l1 / Sp-Z65	WHL22.501991.0	Scaffold421:142867-154459	3	1	1	-
SPU_023463_1	Sp-Wnt4	WHL22.587606.0	Scaffold556:26095-34535	2	1	-	-
SPU_021666	Sp-Dok	WHL22.625930.0	Scaffold628:228164-246066	3	2	-	-
SPU_007721	Sp-Gab	WHL22.28822.0	Scaffold1083:150726-190144	10	3	1	-
SPU_018888	Sp-Z40	WHL22.663957.0	Scaffold711:236982-243132	-	-	-	-
