# Supplementary material for: Developmental gene regulatory network connections predicted by machine learning from gene expression data alone
Source: PLoS One. 2021 Dec 28;16(12):e0261926. doi: 10.1371/journal.pone.0261926 (PMC8714117; doi:10.1371/journal.pone.0261926)
Supplement: S6 File — The spatial expression comparison it its entirely for the Top-50 interactions, as sorted by absolute PEAK confidence scores. Starts with a summary table. For each interaction a separate page displays the spatial expression for each gene in each interaction presented as a color-coded co-expression matrix. (DOCX) [file pone.0261926.s006.docx]

Table summarizing spatial co-expression analysis of the Top50 new PEAK predictions followed by a visualization of expression domains for each interaction. Predictions that matched a known interaction are the rows with a green background. Known spatial expression patterns from 0-30hrs were extracted using the common spatial expression matrix found in the Legacy Echinobase gene pages, in the data section within the Biotapestry Gene Regulatory Networks, or otherwise noted as based on in situ analysis in a particular manuscript. Spatial expression domains for each gene in the predicted interaction were compared and marked as displaying “overlap,” or “non-overlapping” expression. If one or both genes did not have spatial expression data available in the database, the interaction was marked as “missing expression.”

| **Number** | **Node1 ID** | **Node1 Name** | **Node2 ID** | **Node2 name** | **Absolute PEAK Confidence Score** | **Result** |
| --- | --- | --- | --- | --- | --- | --- |
| 1 | SPU_012253 | Eve | SPU_021608 | Hesc | 0.664353216 | overlap |
|  | SPU_013015 | Bra | SPU_006676 | FoxA | 0.512454161 | KNOWN |
| 2 | SPU_012253 | Eve | SPU_020311 | Klf2/4 | 0.401795905 | non-overlapping |
|  | SPU_010424 | Otxa | SPU_027235 | Blimp1a/b | 0.335402485 | KNOWN |
| 3 | SPU_012253 | Eve | SPU_019002 | FoxQ2 | 0.307964581 | non-overlapping |
| 4 | SPU_005358 | Creb-1 | SPU_027235 | Blimp1a/b | 0.304556525 | missing expression |
| 5 | SPU_015456 | Nr1h6b | SPU_027235 | Blimp1a/b | 0.293117698 | missing expression |
| 6 | SPU_002129 | Not | SPU_027235 | Blimp1a/b | 0.289643687 | overlap |
| 7 | SPU_011315 | Atoh8 | SPU_027235 | Blimp1a/b | 0.274355616 | missing expression |
| 8 | SPU_013015 | Bra | SPU_017106 | Deadringer | 0.267043517 | overlap |
| 9 | SPU_012253 | Eve | SPU_020371 | Wnt8 | 0.258509505 | overlap |
| 10 | SPU_013015 | Bra | SPU_000756 | Nkx2.2 | 0.254147541 | overlap |
| 11 | SPU_013015 | Bra | SPU_015374 | Id | 0.251584771 | missing expression |
| 12 | SPU_022817 | Alx1 | SPU_018056 | Homeo1 | 0.231163632 | missing expression |
| (repeat) | SPU_013015 | Bra | SPU_005718 | Deadringer | 0.231031721 |  |
| 13 | SPU_023386 | Tbx2/3 | SPU_017106 | Deadringer | 0.224613713 | overlap |
|  | SPU_012253 | Eve | SPU_000668 | Univin | 0.212374778 | KNOWN |
|  | SPU_022817 | Alx1 | SPU_006462 | Gcm | 0.207381986 | KNOWN |
| 14 | SPU_024903 | Ese | SPU_006462 | Gcm | 0.199851385 | overlap-marginal |
| 15 | SPU_027235 | Blimp1a/b | SPU_006462 | Gcm | 0.193354732 | overlap |
| 16 | SPU_022817 | Alx1 | SPU_005572 | Hmg2 | 0.186558701 | missing expression |
| (repeat) | SPU_023386 | Tbx2/3 | SPU_005718 | Deadringer | 0.185039083 |  |
| 17 | SPU_014576 | Etv1 | SPU_010351 | Irxa | 0.183659044 | missing expression |
| 18 | SPU_023386 | Tbx2/3 | SPU_006814 | HesA | 0.181967776 | missing expression |
| 19 | SPU_022049 | Msx | SPU_000756 | Nkx2.2 | 0.181928593 | overlap |
| 20 | SPU_012490 | Hmx1 | SPU_006462 | Gcm | 0.18181718 | not-overlapping |
| 21 | SPU_011315 | Atoh8 | SPU_006462 | Gcm | 0.181166832 | missing expression |
| 22 | SPU_028583 | Zic1 | SPU_010351 | Irxa | 0.180846956 | missing expression |
| 23 | SPU_012490 | Hmx1 | SPU_010351 | Irxa | 0.180090354 | overlap |
| 24 | SPU_026205 | Mnt | SPU_010351 | Irxa | 0.176152706 | missing expression |
| 25 | SPU_022049 | Msx | SPU_017106 | Deadringer | 0.175520066 | overlap |
|  | SPU_023386 | Tbx2/3 | SPU_010351 | Irxa | 0.175121268 | KNOWN |
| 26 | SPU_010424 | Otxa | SPU_010403 | FoxY | 0.16192926 | overlap |
| 27 | SPU_022817 | Alx1 | SPU_004983 | Chordin | 0.157150787 | not-overlapping |
|  | SPU_022049 | Msx | SPU_010351 | Irxa | 0.155181893 | KNOWN |
| 28 | SPU_022817 | Alx1 | SPU_019024 | Cbp | 0.148934141 | missing expression |
| 29 | SPU_008196 | Hnf1a/b | SPU_010351 | Irxa | 0.148776155 | overlap |
| 30 | SPU_022049 | Msx | SPU_002634 | Hox7 | 0.14783368 | overlap |
| 31 | SPU_000756 | Nkx2.2 | SPU_002634 | Hox7 | 0.14701684 | overlap |
| 32 | SPU_010424 | Otxa | SPU_004983 | Chordin | 0.141624971 | overlap |
| 33 | SPU_006917 | Runt1 | SPU_002634 | Hox7 | 0.141100118 | not-overlapping |
|  | SPU_023386 | Tbx2/3 | SPU_002634 | Hox7 | 0.139950714 | KNOWN |
| --repeat | SPU_022049 | Msx | SPU_005718 | Deadringer | 0.124365216 |  |
| 34 | SPU_004414 | Hlf | SPU_002634 | Hox7 | 0.115652024 | overlap |
| 35 | SPU_002631 | Hox11/13b | SPU_003704 | Tcf | 0.107338606 | missing expression |
| 36 | SPU_010424 | Otxa | SPU_003084 | Gpc6 | 0.093945944 | missing expression |
| 37 | SPU_010424 | Otxa | SPU_004287 | Smad4 | 0.091939819 | missing expression |
|  | SPU_002631 | Hox11/13b | SPU_027235 | Blimp1a/b | 0.084313981 | KNOWN |
| 38 | SPU_002631 | Hox11/13b | SPU_002129 | Not | 0.064496756 | overlap |
| 39 | SPU_002631 | Hox11/13b | SPU_006803 | Creb3 | 0.061841677 | missing expression |
| 40 | SPU_002631 | Hox11/13b | SPU_011583 | Z421 | 0.059092198 | missing expression |
|  | SPU_008196 | Hnf1a/b | SPU_008196 | Hnf1a/b | 0.042171143 | KNOWN |
| 41 | SPU_008196 | Hnf1a/b | SPU_013140 | Ron | 0.04026179 | missing expression |
| 42 | SPU_008196 | Hnf1a/b | SPU_010305 | Hnf1_1 | 0.039554829 | overlap |
| 43 | SPU_008196 | Hnf1a/b | SPU_004287 | Smad4 | 0.038424388 | missing expression |
| 44 | SPU_013569 | Lmo2 | SPU_005572 | Hmg2 | 0.640747321 | missing expression |
| 45 | SPU_021557 | Gapb | SPU_005572 | Hmg2 | 0.634676143 | missing expression |
| 46 | SPU_011635 | FoxJ2_1 | SPU_009155 | Beta-catenin | 0.633262552 | overlap |
| 47 | SPU_023090 | Prickle | SPU_002874 | Ets1/2 | 0.63180003 | missing expression |
| 48 | SPU_018056 | Homeo1 | SPU_002874 | Ets1/2 | 0.631211334 | missing expression |
| 49 | SPU_011189 | Z141 | SPU_009155 | Beta-catenin | 0.629876338 | overlap |
| 50 | SPU_005358 | Crem | SPU_021608 | HesC | 0.628806788 | missing expression |

Predicted interaction: SPU_012253 (Eve) → SPU_021608 (HesC)

Absolute confidence value: 0.664353216

Result: overlap

|  | **Node1: Eve** | **Node2: HesC** | **Both expressed** |
| --- | --- | --- | --- |
| Weak expression |  |  |  |
| Expressed |  |  |  |

|  | **0hr** | **6hr** | **9hr** | **12hr** | **15hr** | **18hr** | **21hr** | **24hr** | **27hr** | **30hr** |
| --- | --- | --- | --- | --- | --- | --- | --- | --- | --- | --- |
| Egg |  |  |  |  |  |  |  |  |  |  |
| Small Micromere |  |  |  |  |  |  |  |  |  |  |
| Skel. Micromere |  |  |  |  |  |  |  |  |  |  |
| Macromere |  |  |  |  |  |  |  |  |  |  |
| V2 |  |  |  |  |  |  |  |  |  |  |
| V2 Meso A |  |  |  |  |  |  |  |  |  |  |
| V2 Meso O |  |  |  |  |  |  |  |  |  |  |
| V2 Endo |  |  |  |  |  |  |  |  |  |  |
| V1 |  |  |  |  |  |  |  |  |  |  |
| V1 Endo A |  |  |  |  |  |  |  |  |  |  |
| V1 Endo O |  |  |  |  |  |  |  |  |  |  |
| V1 Ecto A |  |  |  |  |  |  |  |  |  |  |
| V1 Ecto O |  |  |  |  |  |  |  |  |  |  |
| Mesomere |  |  |  |  |  |  |  |  |  |  |
| Ecto A |  |  |  |  |  |  |  |  |  |  |
| Ecto O |  |  |  |  |  |  |  |  |  |  |
| Stomodeum |  |  |  |  |  |  |  |  |  |  |
| Oral Face |  |  |  |  |  |  |  |  |  |  |
| Ciliated Band |  |  |  |  |  |  |  |  |  |  |
| Apical Plate |  |  |  |  |  |  |  |  |  |  |

Predicted interaction: SPU_012253 (Eve) → SPU_020311 (Klf2/4)

Absolute confidence value: 0.401795905

*Expression for SPU_020311 (Klf2/4) reported in PMID: 16997293

Result: non-overlapping

|  | **Node1: Eve** | **Node2: Klf2/4*** | **Both expressed** |
| --- | --- | --- | --- |
| Weak expression |  |  |  |
| Expressed |  |  |  |

|  | **0hr** | **6hr** | **9hr** | **12hr** | **15hr** | **18hr** | **21hr** | **24hr** | **27hr** | **30hr** |
| --- | --- | --- | --- | --- | --- | --- | --- | --- | --- | --- |
| Egg |  |  |  |  |  |  |  |  |  |  |
| Small Micromere |  |  |  |  |  |  |  |  |  |  |
| Skel. Micromere |  |  |  |  |  |  |  |  |  |  |
| Macromere |  |  |  |  |  |  |  |  |  |  |
| V2 |  |  |  |  |  |  |  |  |  |  |
| V2 Meso A |  |  |  |  |  |  |  |  |  |  |
| V2 Meso O |  |  |  |  |  |  |  |  |  |  |
| V2 Endo |  |  |  |  |  |  |  |  |  |  |
| V1 |  |  |  |  |  |  |  |  |  |  |
| V1 Endo A |  |  |  |  |  |  |  |  |  |  |
| V1 Endo O |  |  |  |  |  |  |  |  |  |  |
| V1 Ecto A |  |  |  |  |  |  |  |  |  |  |
| V1 Ecto O |  |  |  |  |  |  |  |  |  |  |
| Mesomere |  |  |  |  |  |  |  |  |  |  |
| Ecto A |  |  |  |  |  |  |  |  |  |  |
| Ecto O |  |  |  |  |  |  |  |  |  |  |
| Stomodeum |  |  |  |  |  |  |  |  |  |  |
| Oral Face |  |  |  |  |  |  |  |  |  |  |
| Ciliated Band |  |  |  |  |  |  |  |  |  |  |
| Apical Plate |  |  |  |  |  |  |  |  |  |  |

Predicted interaction: SPU_012253 (Eve) → SPU_019002 (FoxQ2)

Absolute confidence value: 0.307964581

Result: non-overlapping

|  | **Node1: Eve** | **Node2: FoxQ2** | **Both expressed** |
| --- | --- | --- | --- |
| Weak expression |  |  |  |
| Expressed |  |  |  |

|  | **0hr** | **6hr** | **9hr** | **12hr** | **15hr** | **18hr** | **21hr** | **24hr** | **27hr** | **30hr** |
| --- | --- | --- | --- | --- | --- | --- | --- | --- | --- | --- |
| Egg |  |  |  |  |  |  |  |  |  |  |
| Small Micromere |  |  |  |  |  |  |  |  |  |  |
| Skel. Micromere |  |  |  |  |  |  |  |  |  |  |
| Macromere |  |  |  |  |  |  |  |  |  |  |
| V2 |  |  |  |  |  |  |  |  |  |  |
| V2 Meso A |  |  |  |  |  |  |  |  |  |  |
| V2 Meso O |  |  |  |  |  |  |  |  |  |  |
| V2 Endo |  |  |  |  |  |  |  |  |  |  |
| V1 |  |  |  |  |  |  |  |  |  |  |
| V1 Endo A |  |  |  |  |  |  |  |  |  |  |
| V1 Endo O |  |  |  |  |  |  |  |  |  |  |
| V1 Ecto A |  |  |  |  |  |  |  |  |  |  |
| V1 Ecto O |  |  |  |  |  |  |  |  |  |  |
| Mesomere |  |  |  |  |  |  |  |  |  |  |
| Ecto A |  |  |  |  |  |  |  |  |  |  |
| Ecto O |  |  |  |  |  |  |  |  |  |  |
| Stomodeum |  |  |  |  |  |  |  |  |  |  |
| Oral Face |  |  |  |  |  |  |  |  |  |  |
| Ciliated Band |  |  |  |  |  |  |  |  |  |  |
| Apical Plate |  |  |  |  |  |  |  |  |  |  |

Predicted interaction: SPU_005358 (Creb-1) → SPU_027235 (Blimb1a/b)

Absolute confidence value: 0.304556525

*No spatial expression data available for SPU_005358 (Creb-1)

Result: missing complete spatial expression data

|  | **Node1: Creb-1*** | **Node2: Blimp1a/b** | **Both expressed** |
| --- | --- | --- | --- |
| Weak expression |  |  |  |
| Expressed |  |  |  |

|  | **0hr** | **6hr** | **9hr** | **12hr** | **15hr** | **18hr** | **21hr** | **24hr** | **27hr** | **30hr** |
| --- | --- | --- | --- | --- | --- | --- | --- | --- | --- | --- |
| Egg |  |  |  |  |  |  |  |  |  |  |
| Small Micromere |  |  |  |  |  |  |  |  |  |  |
| Skel. Micromere |  |  |  |  |  |  |  |  |  |  |
| Macromere |  |  |  |  |  |  |  |  |  |  |
| V2 |  |  |  |  |  |  |  |  |  |  |
| V2 Meso A |  |  |  |  |  |  |  |  |  |  |
| V2 Meso O |  |  |  |  |  |  |  |  |  |  |
| V2 Endo |  |  |  |  |  |  |  |  |  |  |
| V1 |  |  |  |  |  |  |  |  |  |  |
| V1 Endo A |  |  |  |  |  |  |  |  |  |  |
| V1 Endo O |  |  |  |  |  |  |  |  |  |  |
| V1 Ecto A |  |  |  |  |  |  |  |  |  |  |
| V1 Ecto O |  |  |  |  |  |  |  |  |  |  |
| Mesomere |  |  |  |  |  |  |  |  |  |  |
| Ecto A |  |  |  |  |  |  |  |  |  |  |
| Ecto O |  |  |  |  |  |  |  |  |  |  |
| Stomodeum |  |  |  |  |  |  |  |  |  |  |
| Oral Face |  |  |  |  |  |  |  |  |  |  |
| Ciliated Band |  |  |  |  |  |  |  |  |  |  |
| Apical Plate |  |  |  |  |  |  |  |  |  |  |

Predicted interaction: SPU_015456 (Nr1h6b) → SPU_027235 (Blimb1a/b)

Absolute confidence value: 0.293117698

*No spatial expression data available for SPU_015456 (Nr1h6b)

Result: missing complete spatial expression data

|  | **Node1: Nr1h6b*** | **Node2: Blimp1a/b** | **Both expressed** |
| --- | --- | --- | --- |
| Weak expression |  |  |  |
| Expressed |  |  |  |

|  | **0hr** | **6hr** | **9hr** | **12hr** | **15hr** | **18hr** | **21hr** | **24hr** | **27hr** | **30hr** |
| --- | --- | --- | --- | --- | --- | --- | --- | --- | --- | --- |
| Egg |  |  |  |  |  |  |  |  |  |  |
| Small Micromere |  |  |  |  |  |  |  |  |  |  |
| Skel. Micromere |  |  |  |  |  |  |  |  |  |  |
| Macromere |  |  |  |  |  |  |  |  |  |  |
| V2 |  |  |  |  |  |  |  |  |  |  |
| V2 Meso A |  |  |  |  |  |  |  |  |  |  |
| V2 Meso O |  |  |  |  |  |  |  |  |  |  |
| V2 Endo |  |  |  |  |  |  |  |  |  |  |
| V1 |  |  |  |  |  |  |  |  |  |  |
| V1 Endo A |  |  |  |  |  |  |  |  |  |  |
| V1 Endo O |  |  |  |  |  |  |  |  |  |  |
| V1 Ecto A |  |  |  |  |  |  |  |  |  |  |
| V1 Ecto O |  |  |  |  |  |  |  |  |  |  |
| Mesomere |  |  |  |  |  |  |  |  |  |  |
| Ecto A |  |  |  |  |  |  |  |  |  |  |
| Ecto O |  |  |  |  |  |  |  |  |  |  |
| Stomodeum |  |  |  |  |  |  |  |  |  |  |
| Oral Face |  |  |  |  |  |  |  |  |  |  |
| Ciliated Band |  |  |  |  |  |  |  |  |  |  |
| Apical Plate |  |  |  |  |  |  |  |  |  |  |

Predicted interaction: SPU_002129 (Not) → SPU_027235 (Blimb1a/b)

Absolute confidence value: 0.289643687

Result: overlap

|  | **Node1: Not** | **Node2: Blimp1a/b** | **Both expressed** |
| --- | --- | --- | --- |
| Weak expression |  |  |  |
| Expressed |  |  |  |

|  | **0hr** | **6hr** | **9hr** | **12hr** | **15hr** | **18hr** | **21hr** | **24hr** | **27hr** | **30hr** |
| --- | --- | --- | --- | --- | --- | --- | --- | --- | --- | --- |
| Egg |  |  |  |  |  |  |  |  |  |  |
| Small Micromere |  |  |  |  |  |  |  |  |  |  |
| Skel. Micromere |  |  |  |  |  |  |  |  |  |  |
| Macromere |  |  |  |  |  |  |  |  |  |  |
| V2 |  |  |  |  |  |  |  |  |  |  |
| V2 Meso A |  |  |  |  |  |  |  |  |  |  |
| V2 Meso O |  |  |  |  |  |  |  |  |  |  |
| V2 Endo |  |  |  |  |  |  |  |  |  |  |
| V1 |  |  |  |  |  |  |  |  |  |  |
| V1 Endo A |  |  |  |  |  |  |  |  |  |  |
| V1 Endo O |  |  |  |  |  |  |  |  |  |  |
| V1 Ecto A |  |  |  |  |  |  |  |  |  |  |
| V1 Ecto O |  |  |  |  |  |  |  |  |  |  |
| Mesomere |  |  |  |  |  |  |  |  |  |  |
| Ecto A |  |  |  |  |  |  |  |  |  |  |
| Ecto O |  |  |  |  |  |  |  |  |  |  |
| Stomodeum |  |  |  |  |  |  |  |  |  |  |
| Oral Face |  |  |  |  |  |  |  |  |  |  |
| Ciliated Band |  |  |  |  |  |  |  |  |  |  |
| Apical Plate |  |  |  |  |  |  |  |  |  |  |

Predicted interaction: SPU_011315 (Atoh8) → SPU_027235 (Blimb1a/b)

Absolute confidence value: 0.274355616

*No spatial expression data available for SPU_011315 (Atoh8)

Result: missing complete spatial expression data

|  | **Node1: Atoh8*** | **Node2: Blimp1a/b** | **Both expressed** |
| --- | --- | --- | --- |
| Weak expression |  |  |  |
| Expressed |  |  |  |

|  | **0hr** | **6hr** | **9hr** | **12hr** | **15hr** | **18hr** | **21hr** | **24hr** | **27hr** | **30hr** |
| --- | --- | --- | --- | --- | --- | --- | --- | --- | --- | --- |
| Egg |  |  |  |  |  |  |  |  |  |  |
| Small Micromere |  |  |  |  |  |  |  |  |  |  |
| Skel. Micromere |  |  |  |  |  |  |  |  |  |  |
| Macromere |  |  |  |  |  |  |  |  |  |  |
| V2 |  |  |  |  |  |  |  |  |  |  |
| V2 Meso A |  |  |  |  |  |  |  |  |  |  |
| V2 Meso O |  |  |  |  |  |  |  |  |  |  |
| V2 Endo |  |  |  |  |  |  |  |  |  |  |
| V1 |  |  |  |  |  |  |  |  |  |  |
| V1 Endo A |  |  |  |  |  |  |  |  |  |  |
| V1 Endo O |  |  |  |  |  |  |  |  |  |  |
| V1 Ecto A |  |  |  |  |  |  |  |  |  |  |
| V1 Ecto O |  |  |  |  |  |  |  |  |  |  |
| Mesomere |  |  |  |  |  |  |  |  |  |  |
| Ecto A |  |  |  |  |  |  |  |  |  |  |
| Ecto O |  |  |  |  |  |  |  |  |  |  |
| Stomodeum |  |  |  |  |  |  |  |  |  |  |
| Oral Face |  |  |  |  |  |  |  |  |  |  |
| Ciliated Band |  |  |  |  |  |  |  |  |  |  |
| Apical Plate |  |  |  |  |  |  |  |  |  |  |

Predicted interaction: SPU_013015 (Bra) → SPU_017106 (Deadringer)

Absolute confidence value: 0.267043517

Result: overlap

|  | **Node1: Bra** | **Node2: Deadringer** | **Both expressed** |
| --- | --- | --- | --- |
| Weak expression |  |  |  |
| Expressed |  |  |  |

|  | **0hr** | **6hr** | **9hr** | **12hr** | **15hr** | **18hr** | **21hr** | **24hr** | **27hr** | **30hr** |
| --- | --- | --- | --- | --- | --- | --- | --- | --- | --- | --- |
| Egg |  |  |  |  |  |  |  |  |  |  |
| Small Micromere |  |  |  |  |  |  |  |  |  |  |
| Skel. Micromere |  |  |  |  |  |  |  |  |  |  |
| Macromere |  |  |  |  |  |  |  |  |  |  |
| V2 |  |  |  |  |  |  |  |  |  |  |
| V2 Meso A |  |  |  |  |  |  |  |  |  |  |
| V2 Meso O |  |  |  |  |  |  |  |  |  |  |
| V2 Endo |  |  |  |  |  |  |  |  |  |  |
| V1 |  |  |  |  |  |  |  |  |  |  |
| V1 Endo A |  |  |  |  |  |  |  |  |  |  |
| V1 Endo O |  |  |  |  |  |  |  |  |  |  |
| V1 Ecto A |  |  |  |  |  |  |  |  |  |  |
| V1 Ecto O |  |  |  |  |  |  |  |  |  |  |
| Mesomere |  |  |  |  |  |  |  |  |  |  |
| Ecto A |  |  |  |  |  |  |  |  |  |  |
| Ecto O |  |  |  |  |  |  |  |  |  |  |
| Stomodeum |  |  |  |  |  |  |  |  |  |  |
| Oral Face |  |  |  |  |  |  |  |  |  |  |
| Ciliated Band |  |  |  |  |  |  |  |  |  |  |
| Apical Plate |  |  |  |  |  |  |  |  |  |  |

Predicted interaction: SPU_012253 (Eve) → SPU_020371 (Wnt8)

Absolute confidence value: 0.258509505

Result: overlap

|  | **Node1: Eve** | **Node2: Wnt8** | **Both expressed** |
| --- | --- | --- | --- |
| Weak expression |  |  |  |
| Expressed |  |  |  |

|  | **0hr** | **6hr** | **9hr** | **12hr** | **15hr** | **18hr** | **21hr** | **24hr** | **27hr** | **30hr** |
| --- | --- | --- | --- | --- | --- | --- | --- | --- | --- | --- |
| Egg |  |  |  |  |  |  |  |  |  |  |
| Small Micromere |  |  |  |  |  |  |  |  |  |  |
| Skel. Micromere |  |  |  |  |  |  |  |  |  |  |
| Macromere |  |  |  |  |  |  |  |  |  |  |
| V2 |  |  |  |  |  |  |  |  |  |  |
| V2 Meso A |  |  |  |  |  |  |  |  |  |  |
| V2 Meso O |  |  |  |  |  |  |  |  |  |  |
| V2 Endo |  |  |  |  |  |  |  |  |  |  |
| V1 |  |  |  |  |  |  |  |  |  |  |
| V1 Endo A |  |  |  |  |  |  |  |  |  |  |
| V1 Endo O |  |  |  |  |  |  |  |  |  |  |
| V1 Ecto A |  |  |  |  |  |  |  |  |  |  |
| V1 Ecto O |  |  |  |  |  |  |  |  |  |  |
| Mesomere |  |  |  |  |  |  |  |  |  |  |
| Ecto A |  |  |  |  |  |  |  |  |  |  |
| Ecto O |  |  |  |  |  |  |  |  |  |  |
| Stomodeum |  |  |  |  |  |  |  |  |  |  |
| Oral Face |  |  |  |  |  |  |  |  |  |  |
| Ciliated Band |  |  |  |  |  |  |  |  |  |  |
| Apical Plate |  |  |  |  |  |  |  |  |  |  |

Predicted interaction: SPU_013015 (Bra) → SPU_ 000756 (Nkx2.2)

Absolute confidence value: 0.254147541

Result: overlap

|  | **Node1: Bra** | **Node2: Nkx2.2** | **Both expressed** |
| --- | --- | --- | --- |
| Weak expression |  |  |  |
| Expressed |  |  |  |

|  | **0hr** | **6hr** | **9hr** | **12hr** | **15hr** | **18hr** | **21hr** | **24hr** | **27hr** | **30hr** |
| --- | --- | --- | --- | --- | --- | --- | --- | --- | --- | --- |
| Egg |  |  |  |  |  |  |  |  |  |  |
| Small Micromere |  |  |  |  |  |  |  |  |  |  |
| Skel. Micromere |  |  |  |  |  |  |  |  |  |  |
| Macromere |  |  |  |  |  |  |  |  |  |  |
| V2 |  |  |  |  |  |  |  |  |  |  |
| V2 Meso A |  |  |  |  |  |  |  |  |  |  |
| V2 Meso O |  |  |  |  |  |  |  |  |  |  |
| V2 Endo |  |  |  |  |  |  |  |  |  |  |
| V1 |  |  |  |  |  |  |  |  |  |  |
| V1 Endo A |  |  |  |  |  |  |  |  |  |  |
| V1 Endo O |  |  |  |  |  |  |  |  |  |  |
| V1 Ecto A |  |  |  |  |  |  |  |  |  |  |
| V1 Ecto O |  |  |  |  |  |  |  |  |  |  |
| Mesomere |  |  |  |  |  |  |  |  |  |  |
| Ecto A |  |  |  |  |  |  |  |  |  |  |
| Ecto O |  |  |  |  |  |  |  |  |  |  |
| Stomodeum |  |  |  |  |  |  |  |  |  |  |
| Oral Face |  |  |  |  |  |  |  |  |  |  |
| Ciliated Band |  |  |  |  |  |  |  |  |  |  |
| Apical Plate |  |  |  |  |  |  |  |  |  |  |

Predicted interaction: SPU_013015 (Bra) → SPU_015374 (Id)

Absolute confidence value: 0.251584771

*No spatial expression data available for SPU_015374 (Id)

Result: missing complete spatial expression data

|  | **Node1: Bra** | **Node2: Id*** | **Both expressed** |
| --- | --- | --- | --- |
| Weak expression |  |  |  |
| Expressed |  |  |  |

|  | **0hr** | **6hr** | **9hr** | **12hr** | **15hr** | **18hr** | **21hr** | **24hr** | **27hr** | **30hr** |
| --- | --- | --- | --- | --- | --- | --- | --- | --- | --- | --- |
| Egg |  |  |  |  |  |  |  |  |  |  |
| Small Micromere |  |  |  |  |  |  |  |  |  |  |
| Skel. Micromere |  |  |  |  |  |  |  |  |  |  |
| Macromere |  |  |  |  |  |  |  |  |  |  |
| V2 |  |  |  |  |  |  |  |  |  |  |
| V2 Meso A |  |  |  |  |  |  |  |  |  |  |
| V2 Meso O |  |  |  |  |  |  |  |  |  |  |
| V2 Endo |  |  |  |  |  |  |  |  |  |  |
| V1 |  |  |  |  |  |  |  |  |  |  |
| V1 Endo A |  |  |  |  |  |  |  |  |  |  |
| V1 Endo O |  |  |  |  |  |  |  |  |  |  |
| V1 Ecto A |  |  |  |  |  |  |  |  |  |  |
| V1 Ecto O |  |  |  |  |  |  |  |  |  |  |
| Mesomere |  |  |  |  |  |  |  |  |  |  |
| Ecto A |  |  |  |  |  |  |  |  |  |  |
| Ecto O |  |  |  |  |  |  |  |  |  |  |
| Stomodeum |  |  |  |  |  |  |  |  |  |  |
| Oral Face |  |  |  |  |  |  |  |  |  |  |
| Ciliated Band |  |  |  |  |  |  |  |  |  |  |
| Apical Plate |  |  |  |  |  |  |  |  |  |  |

Predicted interaction: SPU_022817 (Alx1) → SPU_018056 (Homeo1)

Absolute confidence value: 0.231163632

*No spatial expression data available for SPU_018056 (Homeo1)

Result: missing complete spatial expression data

|  | **Node1: Alx1** | **Node2: Homeo1*** | **Both expressed** |
| --- | --- | --- | --- |
| Weak expression |  |  |  |
| Expressed |  |  |  |

|  | **0hr** | **6hr** | **9hr** | **12hr** | **15hr** | **18hr** | **21hr** | **24hr** | **27hr** | **30hr** |
| --- | --- | --- | --- | --- | --- | --- | --- | --- | --- | --- |
| Egg |  |  |  |  |  |  |  |  |  |  |
| Small Micromere |  |  |  |  |  |  |  |  |  |  |
| Skel. Micromere |  |  |  |  |  |  |  |  |  |  |
| Macromere |  |  |  |  |  |  |  |  |  |  |
| V2 |  |  |  |  |  |  |  |  |  |  |
| V2 Meso A |  |  |  |  |  |  |  |  |  |  |
| V2 Meso O |  |  |  |  |  |  |  |  |  |  |
| V2 Endo |  |  |  |  |  |  |  |  |  |  |
| V1 |  |  |  |  |  |  |  |  |  |  |
| V1 Endo A |  |  |  |  |  |  |  |  |  |  |
| V1 Endo O |  |  |  |  |  |  |  |  |  |  |
| V1 Ecto A |  |  |  |  |  |  |  |  |  |  |
| V1 Ecto O |  |  |  |  |  |  |  |  |  |  |
| Mesomere |  |  |  |  |  |  |  |  |  |  |
| Ecto A |  |  |  |  |  |  |  |  |  |  |
| Ecto O |  |  |  |  |  |  |  |  |  |  |
| Stomodeum |  |  |  |  |  |  |  |  |  |  |
| Oral Face |  |  |  |  |  |  |  |  |  |  |
| Ciliated Band |  |  |  |  |  |  |  |  |  |  |
| Apical Plate |  |  |  |  |  |  |  |  |  |  |

Predicted interaction: SPU_ 023386 (Tbx2/3) → SPU_017106 (Deadringer)

Absolute confidence value: 0.224613713

Result: overlap

|  | **Node1: Tbx2/3** | **Node2: Deadringer** | **Both expressed** |
| --- | --- | --- | --- |
| Weak expression |  |  |  |
| Expressed |  |  |  |

|  | **0hr** | **6hr** | **9hr** | **12hr** | **15hr** | **18hr** | **21hr** | **24hr** | **27hr** | **30hr** |
| --- | --- | --- | --- | --- | --- | --- | --- | --- | --- | --- |
| Egg |  |  |  |  |  |  |  |  |  |  |
| Small Micromere |  |  |  |  |  |  |  |  |  |  |
| Skel. Micromere |  |  |  |  |  |  |  |  |  |  |
| Macromere |  |  |  |  |  |  |  |  |  |  |
| V2 |  |  |  |  |  |  |  |  |  |  |
| V2 Meso A |  |  |  |  |  |  |  |  |  |  |
| V2 Meso O |  |  |  |  |  |  |  |  |  |  |
| V2 Endo |  |  |  |  |  |  |  |  |  |  |
| V1 |  |  |  |  |  |  |  |  |  |  |
| V1 Endo A |  |  |  |  |  |  |  |  |  |  |
| V1 Endo O |  |  |  |  |  |  |  |  |  |  |
| V1 Ecto A |  |  |  |  |  |  |  |  |  |  |
| V1 Ecto O |  |  |  |  |  |  |  |  |  |  |
| Mesomere |  |  |  |  |  |  |  |  |  |  |
| Ecto A |  |  |  |  |  |  |  |  |  |  |
| Ecto O |  |  |  |  |  |  |  |  |  |  |
| Stomodeum |  |  |  |  |  |  |  |  |  |  |
| Oral Face |  |  |  |  |  |  |  |  |  |  |
| Ciliated Band |  |  |  |  |  |  |  |  |  |  |
| Apical Plate |  |  |  |  |  |  |  |  |  |  |

Predicted interaction: SPU_024903 (Ese) → SPU_006462 (Gcm)

Absolute confidence value: 0.199851385

Result: overlap

|  | **Node1: Ese** | **Node2: Gcm** | **Both expressed** |
| --- | --- | --- | --- |
| Weak expression |  |  |  |
| Expressed |  |  |  |

|  | **0hr** | **6hr** | **9hr** | **12hr** | **15hr** | **18hr** | **21hr** | **24hr** | **27hr** | **30hr** |
| --- | --- | --- | --- | --- | --- | --- | --- | --- | --- | --- |
| Egg |  |  |  |  |  |  |  |  |  |  |
| Small Micromere |  |  |  |  |  |  |  |  |  |  |
| Skel. Micromere |  |  |  |  |  |  |  |  |  |  |
| Macromere |  |  |  |  |  |  |  |  |  |  |
| V2 |  |  |  |  |  |  |  |  |  |  |
| V2 Meso A |  |  |  |  |  |  |  |  |  |  |
| V2 Meso O |  |  |  |  |  |  |  |  |  |  |
| V2 Endo |  |  |  |  |  |  |  |  |  |  |
| V1 |  |  |  |  |  |  |  |  |  |  |
| V1 Endo A |  |  |  |  |  |  |  |  |  |  |
| V1 Endo O |  |  |  |  |  |  |  |  |  |  |
| V1 Ecto A |  |  |  |  |  |  |  |  |  |  |
| V1 Ecto O |  |  |  |  |  |  |  |  |  |  |
| Mesomere |  |  |  |  |  |  |  |  |  |  |
| Ecto A |  |  |  |  |  |  |  |  |  |  |
| Ecto O |  |  |  |  |  |  |  |  |  |  |
| Stomodeum |  |  |  |  |  |  |  |  |  |  |
| Oral Face |  |  |  |  |  |  |  |  |  |  |
| Ciliated Band |  |  |  |  |  |  |  |  |  |  |
| Apical Plate |  |  |  |  |  |  |  |  |  |  |

Predicted interaction: SPU_ SPU_027235(Blimp1a/b) → SPU_006462 (Gcm)

Absolute confidence value: 0.193354732

Result: overlap

|  | **Node1: Blimp1a/b** | **Node2: Gcm** | **Both expressed** |
| --- | --- | --- | --- |
| Weak expression |  |  |  |
| Expressed |  |  |  |

|  | **0hr** | **6hr** | **9hr** | **12hr** | **15hr** | **18hr** | **21hr** | **24hr** | **27hr** | **30hr** |
| --- | --- | --- | --- | --- | --- | --- | --- | --- | --- | --- |
| Egg |  |  |  |  |  |  |  |  |  |  |
| Small Micromere |  |  |  |  |  |  |  |  |  |  |
| Skel. Micromere |  |  |  |  |  |  |  |  |  |  |
| Macromere |  |  |  |  |  |  |  |  |  |  |
| V2 |  |  |  |  |  |  |  |  |  |  |
| V2 Meso A |  |  |  |  |  |  |  |  |  |  |
| V2 Meso O |  |  |  |  |  |  |  |  |  |  |
| V2 Endo |  |  |  |  |  |  |  |  |  |  |
| V1 |  |  |  |  |  |  |  |  |  |  |
| V1 Endo A |  |  |  |  |  |  |  |  |  |  |
| V1 Endo O |  |  |  |  |  |  |  |  |  |  |
| V1 Ecto A |  |  |  |  |  |  |  |  |  |  |
| V1 Ecto O |  |  |  |  |  |  |  |  |  |  |
| Mesomere |  |  |  |  |  |  |  |  |  |  |
| Ecto A |  |  |  |  |  |  |  |  |  |  |
| Ecto O |  |  |  |  |  |  |  |  |  |  |
| Stomodeum |  |  |  |  |  |  |  |  |  |  |
| Oral Face |  |  |  |  |  |  |  |  |  |  |
| Ciliated Band |  |  |  |  |  |  |  |  |  |  |
| Apical Plate |  |  |  |  |  |  |  |  |  |  |

Predicted interaction: SPU_022817 (Alx1) → SPU_005572 (Hmg2)

Absolute confidence value: 0.186558701

*No spatial expression data available for SPU_005572 (Hmg2)

Result: missing complete spatial expression data

|  | **Node1: Alx1** | **Node2: Hmg2*** | **Both expressed** |
| --- | --- | --- | --- |
| Weak expression |  |  |  |
| Expressed |  |  |  |

|  | **0hr** | **6hr** | **9hr** | **12hr** | **15hr** | **18hr** | **21hr** | **24hr** | **27hr** | **30hr** |
| --- | --- | --- | --- | --- | --- | --- | --- | --- | --- | --- |
| Egg |  |  |  |  |  |  |  |  |  |  |
| Small Micromere |  |  |  |  |  |  |  |  |  |  |
| Skel. Micromere |  |  |  |  |  |  |  |  |  |  |
| Macromere |  |  |  |  |  |  |  |  |  |  |
| V2 |  |  |  |  |  |  |  |  |  |  |
| V2 Meso A |  |  |  |  |  |  |  |  |  |  |
| V2 Meso O |  |  |  |  |  |  |  |  |  |  |
| V2 Endo |  |  |  |  |  |  |  |  |  |  |
| V1 |  |  |  |  |  |  |  |  |  |  |
| V1 Endo A |  |  |  |  |  |  |  |  |  |  |
| V1 Endo O |  |  |  |  |  |  |  |  |  |  |
| V1 Ecto A |  |  |  |  |  |  |  |  |  |  |
| V1 Ecto O |  |  |  |  |  |  |  |  |  |  |
| Mesomere |  |  |  |  |  |  |  |  |  |  |
| Ecto A |  |  |  |  |  |  |  |  |  |  |
| Ecto O |  |  |  |  |  |  |  |  |  |  |
| Stomodeum |  |  |  |  |  |  |  |  |  |  |
| Oral Face |  |  |  |  |  |  |  |  |  |  |
| Ciliated Band |  |  |  |  |  |  |  |  |  |  |
| Apical Plate |  |  |  |  |  |  |  |  |  |  |

Predicted interaction: SPU_ (Etv1) → SPU_ (Irxa)

Absolute confidence value: 0.183659044

*Spatial information for SPU_014576 (Etv1/Pea) is not listed in database matrix form

Result: missing complete spatial expression data

|  | **Node1: Etv(Pea)*** | **Node2: Irxa** | **Both expressed** |
| --- | --- | --- | --- |
| Weak expression |  |  |  |
| Expressed |  |  |  |

|  | **0hr** | **6hr** | **9hr** | **12hr** | **15hr** | **18hr** | **21hr** | **24hr** | **27hr** | **30hr** |
| --- | --- | --- | --- | --- | --- | --- | --- | --- | --- | --- |
| Egg |  |  |  |  |  |  |  |  |  |  |
| Small Micromere |  |  |  |  |  |  |  |  |  |  |
| Skel. Micromere |  |  |  |  |  |  |  |  |  |  |
| Macromere |  |  |  |  |  |  |  |  |  |  |
| V2 |  |  |  |  |  |  |  |  |  |  |
| V2 Meso A |  |  |  |  |  |  |  |  |  |  |
| V2 Meso O |  |  |  |  |  |  |  |  |  |  |
| V2 Endo |  |  |  |  |  |  |  |  |  |  |
| V1 |  |  |  |  |  |  |  |  |  |  |
| V1 Endo A |  |  |  |  |  |  |  |  |  |  |
| V1 Endo O |  |  |  |  |  |  |  |  |  |  |
| V1 Ecto A |  |  |  |  |  |  |  |  |  |  |
| V1 Ecto O |  |  |  |  |  |  |  |  |  |  |
| Mesomere |  |  |  |  |  |  |  |  |  |  |
| Ecto A |  |  |  |  |  |  |  |  |  |  |
| Ecto O |  |  |  |  |  |  |  |  |  |  |
| Stomodeum |  |  |  |  |  |  |  |  |  |  |
| Oral Face |  |  |  |  |  |  |  |  |  |  |
| Ciliated Band |  |  |  |  |  |  |  |  |  |  |
| Apical Plate |  |  |  |  |  |  |  |  |  |  |

Predicted interaction: SPU_023386 (Tbx2/3) → SPU_006814 (HesA)

Absolute confidence value: 0.181967776

*No spatial expression data available for SPU_006814 (HesA)

Result: missing complete spatial expression data

|  | **Node1: Tbx2/3** | **Node2: HesA*** | **Both expressed** |
| --- | --- | --- | --- |
| Weak expression |  |  |  |
| Expressed |  |  |  |

|  | **0hr** | **6hr** | **9hr** | **12hr** | **15hr** | **18hr** | **21hr** | **24hr** | **27hr** | **30hr** |
| --- | --- | --- | --- | --- | --- | --- | --- | --- | --- | --- |
| Egg |  |  |  |  |  |  |  |  |  |  |
| Small Micromere |  |  |  |  |  |  |  |  |  |  |
| Skel. Micromere |  |  |  |  |  |  |  |  |  |  |
| Macromere |  |  |  |  |  |  |  |  |  |  |
| V2 |  |  |  |  |  |  |  |  |  |  |
| V2 Meso A |  |  |  |  |  |  |  |  |  |  |
| V2 Meso O |  |  |  |  |  |  |  |  |  |  |
| V2 Endo |  |  |  |  |  |  |  |  |  |  |
| V1 |  |  |  |  |  |  |  |  |  |  |
| V1 Endo A |  |  |  |  |  |  |  |  |  |  |
| V1 Endo O |  |  |  |  |  |  |  |  |  |  |
| V1 Ecto A |  |  |  |  |  |  |  |  |  |  |
| V1 Ecto O |  |  |  |  |  |  |  |  |  |  |
| Mesomere |  |  |  |  |  |  |  |  |  |  |
| Ecto A |  |  |  |  |  |  |  |  |  |  |
| Ecto O |  |  |  |  |  |  |  |  |  |  |
| Stomodeum |  |  |  |  |  |  |  |  |  |  |
| Oral Face |  |  |  |  |  |  |  |  |  |  |
| Ciliated Band |  |  |  |  |  |  |  |  |  |  |
| Apical Plate |  |  |  |  |  |  |  |  |  |  |

Predicted interaction: SPU_022049 (Msx) → SPU_000756 (Nkx2.2)

Absolute confidence value: 0.181928593

Result: overlap

|  | **Node1: Msx** | **Node2: Nkx2.2** | **Both expressed** |
| --- | --- | --- | --- |
| Weak expression |  |  |  |
| Expressed |  |  |  |

|  | **0hr** | **6hr** | **9hr** | **12hr** | **15hr** | **18hr** | **21hr** | **24hr** | **27hr** | **30hr** |
| --- | --- | --- | --- | --- | --- | --- | --- | --- | --- | --- |
| Egg |  |  |  |  |  |  |  |  |  |  |
| Small Micromere |  |  |  |  |  |  |  |  |  |  |
| Skel. Micromere |  |  |  |  |  |  |  |  |  |  |
| Macromere |  |  |  |  |  |  |  |  |  |  |
| V2 |  |  |  |  |  |  |  |  |  |  |
| V2 Meso A |  |  |  |  |  |  |  |  |  |  |
| V2 Meso O |  |  |  |  |  |  |  |  |  |  |
| V2 Endo |  |  |  |  |  |  |  |  |  |  |
| V1 |  |  |  |  |  |  |  |  |  |  |
| V1 Endo A |  |  |  |  |  |  |  |  |  |  |
| V1 Endo O |  |  |  |  |  |  |  |  |  |  |
| V1 Ecto A |  |  |  |  |  |  |  |  |  |  |
| V1 Ecto O |  |  |  |  |  |  |  |  |  |  |
| Mesomere |  |  |  |  |  |  |  |  |  |  |
| Ecto A |  |  |  |  |  |  |  |  |  |  |
| Ecto O |  |  |  |  |  |  |  |  |  |  |
| Stomodeum |  |  |  |  |  |  |  |  |  |  |
| Oral Face |  |  |  |  |  |  |  |  |  |  |
| Ciliated Band |  |  |  |  |  |  |  |  |  |  |
| Apical Plate |  |  |  |  |  |  |  |  |  |  |

Predicted interaction: SPU_012490 (Hmx) → SPU_006462 (Gcm)

Absolute confidence value: 0.18181718

Result: non-overlapping

|  | **Node1: Hmx1** | **Node2: Gcm** | **Both expressed** |
| --- | --- | --- | --- |
| Weak expression |  |  |  |
| Expressed |  |  |  |

|  | **0hr** | **6hr** | **9hr** | **12hr** | **15hr** | **18hr** | **21hr** | **24hr** | **27hr** | **30hr** |
| --- | --- | --- | --- | --- | --- | --- | --- | --- | --- | --- |
| Egg |  |  |  |  |  |  |  |  |  |  |
| Small Micromere |  |  |  |  |  |  |  |  |  |  |
| Skel. Micromere |  |  |  |  |  |  |  |  |  |  |
| Macromere |  |  |  |  |  |  |  |  |  |  |
| V2 |  |  |  |  |  |  |  |  |  |  |
| V2 Meso A |  |  |  |  |  |  |  |  |  |  |
| V2 Meso O |  |  |  |  |  |  |  |  |  |  |
| V2 Endo |  |  |  |  |  |  |  |  |  |  |
| V1 |  |  |  |  |  |  |  |  |  |  |
| V1 Endo A |  |  |  |  |  |  |  |  |  |  |
| V1 Endo O |  |  |  |  |  |  |  |  |  |  |
| V1 Ecto A |  |  |  |  |  |  |  |  |  |  |
| V1 Ecto O |  |  |  |  |  |  |  |  |  |  |
| Mesomere |  |  |  |  |  |  |  |  |  |  |
| Ecto A |  |  |  |  |  |  |  |  |  |  |
| Ecto O |  |  |  |  |  |  |  |  |  |  |
| Stomodeum |  |  |  |  |  |  |  |  |  |  |
| Oral Face |  |  |  |  |  |  |  |  |  |  |
| Ciliated Band |  |  |  |  |  |  |  |  |  |  |
| Apical Plate |  |  |  |  |  |  |  |  |  |  |

Predicted interaction: SPU_011315 (Atoh8) → SPU_006462 (Gcm)

Absolute confidence value: 0.181166832

*No spatial expression data available for SPU_011315 (Atoh8)

Result: missing complete spatial expression data

|  | **Node1: Atoh8*** | **Node2: Gcm** | **Both expressed** |
| --- | --- | --- | --- |
| Weak expression |  |  |  |
| Expressed |  |  |  |

|  | **0hr** | **6hr** | **9hr** | **12hr** | **15hr** | **18hr** | **21hr** | **24hr** | **27hr** | **30hr** |
| --- | --- | --- | --- | --- | --- | --- | --- | --- | --- | --- |
| Egg |  |  |  |  |  |  |  |  |  |  |
| Small Micromere |  |  |  |  |  |  |  |  |  |  |
| Skel. Micromere |  |  |  |  |  |  |  |  |  |  |
| Macromere |  |  |  |  |  |  |  |  |  |  |
| V2 |  |  |  |  |  |  |  |  |  |  |
| V2 Meso A |  |  |  |  |  |  |  |  |  |  |
| V2 Meso O |  |  |  |  |  |  |  |  |  |  |
| V2 Endo |  |  |  |  |  |  |  |  |  |  |
| V1 |  |  |  |  |  |  |  |  |  |  |
| V1 Endo A |  |  |  |  |  |  |  |  |  |  |
| V1 Endo O |  |  |  |  |  |  |  |  |  |  |
| V1 Ecto A |  |  |  |  |  |  |  |  |  |  |
| V1 Ecto O |  |  |  |  |  |  |  |  |  |  |
| Mesomere |  |  |  |  |  |  |  |  |  |  |
| Ecto A |  |  |  |  |  |  |  |  |  |  |
| Ecto O |  |  |  |  |  |  |  |  |  |  |
| Stomodeum |  |  |  |  |  |  |  |  |  |  |
| Oral Face |  |  |  |  |  |  |  |  |  |  |
| Ciliated Band |  |  |  |  |  |  |  |  |  |  |
| Apical Plate |  |  |  |  |  |  |  |  |  |  |

Predicted interaction: SPU_028583 (Zic1) → SPU_010351 (IrxA)

Absolute confidence value: 0.180846956

*No spatial expression data available for SPU_028583 (Zic1)

Result: missing complete spatial expression data

|  | **Node1: Zic1*** | **Node2: IrxA** | **Both expressed** |
| --- | --- | --- | --- |
| Weak expression |  |  |  |
| Expressed |  |  |  |

|  | **0hr** | **6hr** | **9hr** | **12hr** | **15hr** | **18hr** | **21hr** | **24hr** | **27hr** | **30hr** |
| --- | --- | --- | --- | --- | --- | --- | --- | --- | --- | --- |
| Egg |  |  |  |  |  |  |  |  |  |  |
| Small Micromere |  |  |  |  |  |  |  |  |  |  |
| Skel. Micromere |  |  |  |  |  |  |  |  |  |  |
| Macromere |  |  |  |  |  |  |  |  |  |  |
| V2 |  |  |  |  |  |  |  |  |  |  |
| V2 Meso A |  |  |  |  |  |  |  |  |  |  |
| V2 Meso O |  |  |  |  |  |  |  |  |  |  |
| V2 Endo |  |  |  |  |  |  |  |  |  |  |
| V1 |  |  |  |  |  |  |  |  |  |  |
| V1 Endo A |  |  |  |  |  |  |  |  |  |  |
| V1 Endo O |  |  |  |  |  |  |  |  |  |  |
| V1 Ecto A |  |  |  |  |  |  |  |  |  |  |
| V1 Ecto O |  |  |  |  |  |  |  |  |  |  |
| Mesomere |  |  |  |  |  |  |  |  |  |  |
| Ecto A |  |  |  |  |  |  |  |  |  |  |
| Ecto O |  |  |  |  |  |  |  |  |  |  |
| Stomodeum |  |  |  |  |  |  |  |  |  |  |
| Oral Face |  |  |  |  |  |  |  |  |  |  |
| Ciliated Band |  |  |  |  |  |  |  |  |  |  |
| Apical Plate |  |  |  |  |  |  |  |  |  |  |

Predicted interaction: SPU_012490 (Hmx1) → SPU_010351 (IrxA)

Absolute confidence value: 0.180090354

Result: overlap

|  | **Node1: Hmx1** | **Node2: IrxA** | **Both expressed** |
| --- | --- | --- | --- |
| Weak expression |  |  |  |
| Expressed |  |  |  |

|  | **0hr** | **6hr** | **9hr** | **12hr** | **15hr** | **18hr** | **21hr** | **24hr** | **27hr** | **30hr** |
| --- | --- | --- | --- | --- | --- | --- | --- | --- | --- | --- |
| Egg |  |  |  |  |  |  |  |  |  |  |
| Small Micromere |  |  |  |  |  |  |  |  |  |  |
| Skel. Micromere |  |  |  |  |  |  |  |  |  |  |
| Macromere |  |  |  |  |  |  |  |  |  |  |
| V2 |  |  |  |  |  |  |  |  |  |  |
| V2 Meso A |  |  |  |  |  |  |  |  |  |  |
| V2 Meso O |  |  |  |  |  |  |  |  |  |  |
| V2 Endo |  |  |  |  |  |  |  |  |  |  |
| V1 |  |  |  |  |  |  |  |  |  |  |
| V1 Endo A |  |  |  |  |  |  |  |  |  |  |
| V1 Endo O |  |  |  |  |  |  |  |  |  |  |
| V1 Ecto A |  |  |  |  |  |  |  |  |  |  |
| V1 Ecto O |  |  |  |  |  |  |  |  |  |  |
| Mesomere |  |  |  |  |  |  |  |  |  |  |
| Ecto A |  |  |  |  |  |  |  |  |  |  |
| Ecto O |  |  |  |  |  |  |  |  |  |  |
| Stomodeum |  |  |  |  |  |  |  |  |  |  |
| Oral Face |  |  |  |  |  |  |  |  |  |  |
| Ciliated Band |  |  |  |  |  |  |  |  |  |  |
| Apical Plate |  |  |  |  |  |  |  |  |  |  |

Predicted interaction: SPU_026205 (Mnt) → SPU_010351 (IrxA)

Absolute confidence value: 0.176152706

*No spatial expression data available for SPU_026205 (Mnt)

Result: missing complete spatial expression data

|  | **Node1: Mnt*** | **Node2: IrxA** | **Both expressed** |
| --- | --- | --- | --- |
| Weak expression |  |  |  |
| Expressed |  |  |  |

|  | **0hr** | **6hr** | **9hr** | **12hr** | **15hr** | **18hr** | **21hr** | **24hr** | **27hr** | **30hr** |
| --- | --- | --- | --- | --- | --- | --- | --- | --- | --- | --- |
| Egg |  |  |  |  |  |  |  |  |  |  |
| Small Micromere |  |  |  |  |  |  |  |  |  |  |
| Skel. Micromere |  |  |  |  |  |  |  |  |  |  |
| Macromere |  |  |  |  |  |  |  |  |  |  |
| V2 |  |  |  |  |  |  |  |  |  |  |
| V2 Meso A |  |  |  |  |  |  |  |  |  |  |
| V2 Meso O |  |  |  |  |  |  |  |  |  |  |
| V2 Endo |  |  |  |  |  |  |  |  |  |  |
| V1 |  |  |  |  |  |  |  |  |  |  |
| V1 Endo A |  |  |  |  |  |  |  |  |  |  |
| V1 Endo O |  |  |  |  |  |  |  |  |  |  |
| V1 Ecto A |  |  |  |  |  |  |  |  |  |  |
| V1 Ecto O |  |  |  |  |  |  |  |  |  |  |
| Mesomere |  |  |  |  |  |  |  |  |  |  |
| Ecto A |  |  |  |  |  |  |  |  |  |  |
| Ecto O |  |  |  |  |  |  |  |  |  |  |
| Stomodeum |  |  |  |  |  |  |  |  |  |  |
| Oral Face |  |  |  |  |  |  |  |  |  |  |
| Ciliated Band |  |  |  |  |  |  |  |  |  |  |
| Apical Plate |  |  |  |  |  |  |  |  |  |  |

Predicted interaction: SPU_022049 (Msx) → SPU_017106 (Deadringer)

Absolute confidence value: 0.175520066

Result: overlap

|  | **Node1: Msx** | **Node2: Deadringer** | **Both expressed** |
| --- | --- | --- | --- |
| Weak expression |  |  |  |
| Expressed |  |  |  |

|  | **0hr** | **6hr** | **9hr** | **12hr** | **15hr** | **18hr** | **21hr** | **24hr** | **27hr** | **30hr** |
| --- | --- | --- | --- | --- | --- | --- | --- | --- | --- | --- |
| Egg |  |  |  |  |  |  |  |  |  |  |
| Small Micromere |  |  |  |  |  |  |  |  |  |  |
| Skel. Micromere |  |  |  |  |  |  |  |  |  |  |
| Macromere |  |  |  |  |  |  |  |  |  |  |
| V2 |  |  |  |  |  |  |  |  |  |  |
| V2 Meso A |  |  |  |  |  |  |  |  |  |  |
| V2 Meso O |  |  |  |  |  |  |  |  |  |  |
| V2 Endo |  |  |  |  |  |  |  |  |  |  |
| V1 |  |  |  |  |  |  |  |  |  |  |
| V1 Endo A |  |  |  |  |  |  |  |  |  |  |
| V1 Endo O |  |  |  |  |  |  |  |  |  |  |
| V1 Ecto A |  |  |  |  |  |  |  |  |  |  |
| V1 Ecto O |  |  |  |  |  |  |  |  |  |  |
| Mesomere |  |  |  |  |  |  |  |  |  |  |
| Ecto A |  |  |  |  |  |  |  |  |  |  |
| Ecto O |  |  |  |  |  |  |  |  |  |  |
| Stomodeum |  |  |  |  |  |  |  |  |  |  |
| Oral Face |  |  |  |  |  |  |  |  |  |  |
| Ciliated Band |  |  |  |  |  |  |  |  |  |  |
| Apical Plate |  |  |  |  |  |  |  |  |  |  |

Predicted interaction: SPU_010424 (OtxA) → SPU_010403 (FoxY)

Absolute confidence value: 0.16192926

Result: overlap

|  | **Node1: OtxA** | **Node2: FoxY** | **Both expressed** |
| --- | --- | --- | --- |
| Weak expression |  |  |  |
| Expressed |  |  |  |

|  | **0hr** | **6hr** | **9hr** | **12hr** | **15hr** | **18hr** | **21hr** | **24hr** | **27hr** | **30hr** |
| --- | --- | --- | --- | --- | --- | --- | --- | --- | --- | --- |
| Egg |  |  |  |  |  |  |  |  |  |  |
| Small Micromere |  |  |  |  |  |  |  |  |  |  |
| Skel. Micromere |  |  |  |  |  |  |  |  |  |  |
| Macromere |  |  |  |  |  |  |  |  |  |  |
| V2 |  |  |  |  |  |  |  |  |  |  |
| V2 Meso A |  |  |  |  |  |  |  |  |  |  |
| V2 Meso O |  |  |  |  |  |  |  |  |  |  |
| V2 Endo |  |  |  |  |  |  |  |  |  |  |
| V1 |  |  |  |  |  |  |  |  |  |  |
| V1 Endo A |  |  |  |  |  |  |  |  |  |  |
| V1 Endo O |  |  |  |  |  |  |  |  |  |  |
| V1 Ecto A |  |  |  |  |  |  |  |  |  |  |
| V1 Ecto O |  |  |  |  |  |  |  |  |  |  |
| Mesomere |  |  |  |  |  |  |  |  |  |  |
| Ecto A |  |  |  |  |  |  |  |  |  |  |
| Ecto O |  |  |  |  |  |  |  |  |  |  |
| Stomodeum |  |  |  |  |  |  |  |  |  |  |
| Oral Face |  |  |  |  |  |  |  |  |  |  |
| Ciliated Band |  |  |  |  |  |  |  |  |  |  |
| Apical Plate |  |  |  |  |  |  |  |  |  |  |

Predicted interaction: SPU_022817 (Alx1) → SPU_004983 (Chordin)

Absolute confidence value: 0.157150787

Result: non-overlapping expression

|  | **Node1: Alx1** | **Node2: Chordin** | **Both expressed** |
| --- | --- | --- | --- |
| Weak expression |  |  |  |
| Expressed |  |  |  |

|  | **0hr** | **6hr** | **9hr** | **12hr** | **15hr** | **18hr** | **21hr** | **24hr** | **27hr** | **30hr** |
| --- | --- | --- | --- | --- | --- | --- | --- | --- | --- | --- |
| Egg |  |  |  |  |  |  |  |  |  |  |
| Small Micromere |  |  |  |  |  |  |  |  |  |  |
| Skel. Micromere |  |  |  |  |  |  |  |  |  |  |
| Macromere |  |  |  |  |  |  |  |  |  |  |
| V2 |  |  |  |  |  |  |  |  |  |  |
| V2 Meso A |  |  |  |  |  |  |  |  |  |  |
| V2 Meso O |  |  |  |  |  |  |  |  |  |  |
| V2 Endo |  |  |  |  |  |  |  |  |  |  |
| V1 |  |  |  |  |  |  |  |  |  |  |
| V1 Endo A |  |  |  |  |  |  |  |  |  |  |
| V1 Endo O |  |  |  |  |  |  |  |  |  |  |
| V1 Ecto A |  |  |  |  |  |  |  |  |  |  |
| V1 Ecto O |  |  |  |  |  |  |  |  |  |  |
| Mesomere |  |  |  |  |  |  |  |  |  |  |
| Ecto A |  |  |  |  |  |  |  |  |  |  |
| Ecto O |  |  |  |  |  |  |  |  |  |  |
| Stomodeum |  |  |  |  |  |  |  |  |  |  |
| Oral Face |  |  |  |  |  |  |  |  |  |  |
| Ciliated Band |  |  |  |  |  |  |  |  |  |  |
| Apical Plate |  |  |  |  |  |  |  |  |  |  |

Predicted interaction: SPU_022817 (Alx1) → SPU_019024 (Cbp)

Absolute confidence value: 0.148934141

*No spatial expression data available for SPU_019024 (Cbp)

Result: missing complete spatial expression data

|  | **Node1: Alx1** | **Node2: Cbp*** | **Both expressed** |
| --- | --- | --- | --- |
| Weak expression |  |  |  |
| Expressed |  |  |  |

|  | **0hr** | **6hr** | **9hr** | **12hr** | **15hr** | **18hr** | **21hr** | **24hr** | **27hr** | **30hr** |
| --- | --- | --- | --- | --- | --- | --- | --- | --- | --- | --- |
| Egg |  |  |  |  |  |  |  |  |  |  |
| Small Micromere |  |  |  |  |  |  |  |  |  |  |
| Skel. Micromere |  |  |  |  |  |  |  |  |  |  |
| Macromere |  |  |  |  |  |  |  |  |  |  |
| V2 |  |  |  |  |  |  |  |  |  |  |
| V2 Meso A |  |  |  |  |  |  |  |  |  |  |
| V2 Meso O |  |  |  |  |  |  |  |  |  |  |
| V2 Endo |  |  |  |  |  |  |  |  |  |  |
| V1 |  |  |  |  |  |  |  |  |  |  |
| V1 Endo A |  |  |  |  |  |  |  |  |  |  |
| V1 Endo O |  |  |  |  |  |  |  |  |  |  |
| V1 Ecto A |  |  |  |  |  |  |  |  |  |  |
| V1 Ecto O |  |  |  |  |  |  |  |  |  |  |
| Mesomere |  |  |  |  |  |  |  |  |  |  |
| Ecto A |  |  |  |  |  |  |  |  |  |  |
| Ecto O |  |  |  |  |  |  |  |  |  |  |
| Stomodeum |  |  |  |  |  |  |  |  |  |  |
| Oral Face |  |  |  |  |  |  |  |  |  |  |
| Ciliated Band |  |  |  |  |  |  |  |  |  |  |
| Apical Plate |  |  |  |  |  |  |  |  |  |  |

Predicted interaction: SPU_008196 (Hnf1a/b) → SPU_010351 (IrxA)

Absolute confidence value: 0.148776155

Result: overlap

|  | **Node1: Hnf1a/b** | **Node2: IrxA** | **Both expressed** |
| --- | --- | --- | --- |
| Weak expression |  |  |  |
| Expressed |  |  |  |

|  | **0hr** | **6hr** | **9hr** | **12hr** | **15hr** | **18hr** | **21hr** | **24hr** | **27hr** | **30hr** |
| --- | --- | --- | --- | --- | --- | --- | --- | --- | --- | --- |
| Egg |  |  |  |  |  |  |  |  |  |  |
| Small Micromere |  |  |  |  |  |  |  |  |  |  |
| Skel. Micromere |  |  |  |  |  |  |  |  |  |  |
| Macromere |  |  |  |  |  |  |  |  |  |  |
| V2 |  |  |  |  |  |  |  |  |  |  |
| V2 Meso A |  |  |  |  |  |  |  |  |  |  |
| V2 Meso O |  |  |  |  |  |  |  |  |  |  |
| V2 Endo |  |  |  |  |  |  |  |  |  |  |
| V1 |  |  |  |  |  |  |  |  |  |  |
| V1 Endo A |  |  |  |  |  |  |  |  |  |  |
| V1 Endo O |  |  |  |  |  |  |  |  |  |  |
| V1 Ecto A |  |  |  |  |  |  |  |  |  |  |
| V1 Ecto O |  |  |  |  |  |  |  |  |  |  |
| Mesomere |  |  |  |  |  |  |  |  |  |  |
| Ecto A |  |  |  |  |  |  |  |  |  |  |
| Ecto O |  |  |  |  |  |  |  |  |  |  |
| Stomodeum |  |  |  |  |  |  |  |  |  |  |
| Oral Face |  |  |  |  |  |  |  |  |  |  |
| Ciliated Band |  |  |  |  |  |  |  |  |  |  |
| Apical Plate |  |  |  |  |  |  |  |  |  |  |

Predicted interaction: SPU_022049 (Msx) → SPU_002634 (Hox7)

Absolute confidence value: 0.14783368

Result: overlap

|  | **Node1: Msx** | **Node2: Hox7** | **Both expressed** |
| --- | --- | --- | --- |
| Weak expression |  |  |  |
| Expressed |  |  |  |

|  | **0hr** | **6hr** | **9hr** | **12hr** | **15hr** | **18hr** | **21hr** | **24hr** | **27hr** | **30hr** |
| --- | --- | --- | --- | --- | --- | --- | --- | --- | --- | --- |
| Egg |  |  |  |  |  |  |  |  |  |  |
| Small Micromere |  |  |  |  |  |  |  |  |  |  |
| Skel. Micromere |  |  |  |  |  |  |  |  |  |  |
| Macromere |  |  |  |  |  |  |  |  |  |  |
| V2 |  |  |  |  |  |  |  |  |  |  |
| V2 Meso A |  |  |  |  |  |  |  |  |  |  |
| V2 Meso O |  |  |  |  |  |  |  |  |  |  |
| V2 Endo |  |  |  |  |  |  |  |  |  |  |
| V1 |  |  |  |  |  |  |  |  |  |  |
| V1 Endo A |  |  |  |  |  |  |  |  |  |  |
| V1 Endo O |  |  |  |  |  |  |  |  |  |  |
| V1 Ecto A |  |  |  |  |  |  |  |  |  |  |
| V1 Ecto O |  |  |  |  |  |  |  |  |  |  |
| Mesomere |  |  |  |  |  |  |  |  |  |  |
| Ecto A |  |  |  |  |  |  |  |  |  |  |
| Ecto O |  |  |  |  |  |  |  |  |  |  |
| Stomodeum |  |  |  |  |  |  |  |  |  |  |
| Oral Face |  |  |  |  |  |  |  |  |  |  |
| Ciliated Band |  |  |  |  |  |  |  |  |  |  |
| Apical Plate |  |  |  |  |  |  |  |  |  |  |

Predicted interaction: SPU_000756 (Nkx2.2) → SPU_002634 (Hox7)

Absolute confidence value: 0.14701684

Result: overlap

|  | **Node1: Nkx2.2** | **Node2: Hox7** | **Both expressed** |
| --- | --- | --- | --- |
| Weak expression |  |  |  |
| Expressed |  |  |  |

|  | **0hr** | **6hr** | **9hr** | **12hr** | **15hr** | **18hr** | **21hr** | **24hr** | **27hr** | **30hr** |
| --- | --- | --- | --- | --- | --- | --- | --- | --- | --- | --- |
| Egg |  |  |  |  |  |  |  |  |  |  |
| Small Micromere |  |  |  |  |  |  |  |  |  |  |
| Skel. Micromere |  |  |  |  |  |  |  |  |  |  |
| Macromere |  |  |  |  |  |  |  |  |  |  |
| V2 |  |  |  |  |  |  |  |  |  |  |
| V2 Meso A |  |  |  |  |  |  |  |  |  |  |
| V2 Meso O |  |  |  |  |  |  |  |  |  |  |
| V2 Endo |  |  |  |  |  |  |  |  |  |  |
| V1 |  |  |  |  |  |  |  |  |  |  |
| V1 Endo A |  |  |  |  |  |  |  |  |  |  |
| V1 Endo O |  |  |  |  |  |  |  |  |  |  |
| V1 Ecto A |  |  |  |  |  |  |  |  |  |  |
| V1 Ecto O |  |  |  |  |  |  |  |  |  |  |
| Mesomere |  |  |  |  |  |  |  |  |  |  |
| Ecto A |  |  |  |  |  |  |  |  |  |  |
| Ecto O |  |  |  |  |  |  |  |  |  |  |
| Stomodeum |  |  |  |  |  |  |  |  |  |  |
| Oral Face |  |  |  |  |  |  |  |  |  |  |
| Ciliated Band |  |  |  |  |  |  |  |  |  |  |
| Apical Plate |  |  |  |  |  |  |  |  |  |  |

Predicted interaction: SPU_010424 (OtxA) → SPU_004983 (Chordin)

Absolute confidence value: 0.141624971

Result: overlap

|  | **Node1: OtxA** | **Node2: Chordin** | **Both expressed** |
| --- | --- | --- | --- |
| Weak expression |  |  |  |
| Expressed |  |  |  |

|  | **0hr** | **6hr** | **9hr** | **12hr** | **15hr** | **18hr** | **21hr** | **24hr** | **27hr** | **30hr** |
| --- | --- | --- | --- | --- | --- | --- | --- | --- | --- | --- |
| Egg |  |  |  |  |  |  |  |  |  |  |
| Small Micromere |  |  |  |  |  |  |  |  |  |  |
| Skel. Micromere |  |  |  |  |  |  |  |  |  |  |
| Macromere |  |  |  |  |  |  |  |  |  |  |
| V2 |  |  |  |  |  |  |  |  |  |  |
| V2 Meso A |  |  |  |  |  |  |  |  |  |  |
| V2 Meso O |  |  |  |  |  |  |  |  |  |  |
| V2 Endo |  |  |  |  |  |  |  |  |  |  |
| V1 |  |  |  |  |  |  |  |  |  |  |
| V1 Endo A |  |  |  |  |  |  |  |  |  |  |
| V1 Endo O |  |  |  |  |  |  |  |  |  |  |
| V1 Ecto A |  |  |  |  |  |  |  |  |  |  |
| V1 Ecto O |  |  |  |  |  |  |  |  |  |  |
| Mesomere |  |  |  |  |  |  |  |  |  |  |
| Ecto A |  |  |  |  |  |  |  |  |  |  |
| Ecto O |  |  |  |  |  |  |  |  |  |  |
| Stomodeum |  |  |  |  |  |  |  |  |  |  |
| Oral Face |  |  |  |  |  |  |  |  |  |  |
| Ciliated Band |  |  |  |  |  |  |  |  |  |  |
| Apical Plate |  |  |  |  |  |  |  |  |  |  |

Predicted interaction: SPU_006917 (Runt1) → SPU_002634 (Hox7)

Absolute confidence value: 0.141100118

Result: non-overlapping expression

|  | **Node1: Runt1** | **Node2: Hox7** | **Both expressed** |
| --- | --- | --- | --- |
| Weak expression |  |  |  |
| Expressed |  |  |  |

|  | **0hr** | **6hr** | **9hr** | **12hr** | **15hr** | **18hr** | **21hr** | **24hr** | **27hr** | **30hr** |
| --- | --- | --- | --- | --- | --- | --- | --- | --- | --- | --- |
| Egg |  |  |  |  |  |  |  |  |  |  |
| Small Micromere |  |  |  |  |  |  |  |  |  |  |
| Skel. Micromere |  |  |  |  |  |  |  |  |  |  |
| Macromere |  |  |  |  |  |  |  |  |  |  |
| V2 |  |  |  |  |  |  |  |  |  |  |
| V2 Meso A |  |  |  |  |  |  |  |  |  |  |
| V2 Meso O |  |  |  |  |  |  |  |  |  |  |
| V2 Endo |  |  |  |  |  |  |  |  |  |  |
| V1 |  |  |  |  |  |  |  |  |  |  |
| V1 Endo A |  |  |  |  |  |  |  |  |  |  |
| V1 Endo O |  |  |  |  |  |  |  |  |  |  |
| V1 Ecto A |  |  |  |  |  |  |  |  |  |  |
| V1 Ecto O |  |  |  |  |  |  |  |  |  |  |
| Mesomere |  |  |  |  |  |  |  |  |  |  |
| Ecto A |  |  |  |  |  |  |  |  |  |  |
| Ecto O |  |  |  |  |  |  |  |  |  |  |
| Stomodeum |  |  |  |  |  |  |  |  |  |  |
| Oral Face |  |  |  |  |  |  |  |  |  |  |
| Ciliated Band |  |  |  |  |  |  |  |  |  |  |
| Apical Plate |  |  |  |  |  |  |  |  |  |  |

Predicted interaction: SPU_004414 (Hlf) → SPU_002634 (Hox7)

Absolute confidence value: 0.115652024

Result: overlap

|  | **Node1: Hlf** | **Node2: Hox7** | **Both expressed** |
| --- | --- | --- | --- |
| Weak expression |  |  |  |
| Expressed |  |  |  |

|  | **0hr** | **6hr** | **9hr** | **12hr** | **15hr** | **18hr** | **21hr** | **24hr** | **27hr** | **30hr** |
| --- | --- | --- | --- | --- | --- | --- | --- | --- | --- | --- |
| Egg |  |  |  |  |  |  |  |  |  |  |
| Small Micromere |  |  |  |  |  |  |  |  |  |  |
| Skel. Micromere |  |  |  |  |  |  |  |  |  |  |
| Macromere |  |  |  |  |  |  |  |  |  |  |
| V2 |  |  |  |  |  |  |  |  |  |  |
| V2 Meso A |  |  |  |  |  |  |  |  |  |  |
| V2 Meso O |  |  |  |  |  |  |  |  |  |  |
| V2 Endo |  |  |  |  |  |  |  |  |  |  |
| V1 |  |  |  |  |  |  |  |  |  |  |
| V1 Endo A |  |  |  |  |  |  |  |  |  |  |
| V1 Endo O |  |  |  |  |  |  |  |  |  |  |
| V1 Ecto A |  |  |  |  |  |  |  |  |  |  |
| V1 Ecto O |  |  |  |  |  |  |  |  |  |  |
| Mesomere |  |  |  |  |  |  |  |  |  |  |
| Ecto A |  |  |  |  |  |  |  |  |  |  |
| Ecto O |  |  |  |  |  |  |  |  |  |  |
| Stomodeum |  |  |  |  |  |  |  |  |  |  |
| Oral Face |  |  |  |  |  |  |  |  |  |  |
| Ciliated Band |  |  |  |  |  |  |  |  |  |  |
| Apical Plate |  |  |  |  |  |  |  |  |  |  |

Predicted interaction: SPU_002631 (Hox11/13b) → SPU_003704 (Tcf)

Absolute confidence value: 0.107338606

*No spatial expression information for (SPU_003704) Tcf

Result: missing complete spatial expression data

|  | **Node1: Hox11/13b** | **Node2: Tcf*** | **Both expressed** |
| --- | --- | --- | --- |
| Weak expression |  |  |  |
| Expressed |  |  |  |

|  | **0hr** | **6hr** | **9hr** | **12hr** | **15hr** | **18hr** | **21hr** | **24hr** | **27hr** | **30hr** |
| --- | --- | --- | --- | --- | --- | --- | --- | --- | --- | --- |
| Egg |  |  |  |  |  |  |  |  |  |  |
| Small Micromere |  |  |  |  |  |  |  |  |  |  |
| Skel. Micromere |  |  |  |  |  |  |  |  |  |  |
| Macromere |  |  |  |  |  |  |  |  |  |  |
| V2 |  |  |  |  |  |  |  |  |  |  |
| V2 Meso A |  |  |  |  |  |  |  |  |  |  |
| V2 Meso O |  |  |  |  |  |  |  |  |  |  |
| V2 Endo |  |  |  |  |  |  |  |  |  |  |
| V1 |  |  |  |  |  |  |  |  |  |  |
| V1 Endo A |  |  |  |  |  |  |  |  |  |  |
| V1 Endo O |  |  |  |  |  |  |  |  |  |  |
| V1 Ecto A |  |  |  |  |  |  |  |  |  |  |
| V1 Ecto O |  |  |  |  |  |  |  |  |  |  |
| Mesomere |  |  |  |  |  |  |  |  |  |  |
| Ecto A |  |  |  |  |  |  |  |  |  |  |
| Ecto O |  |  |  |  |  |  |  |  |  |  |
| Stomodeum |  |  |  |  |  |  |  |  |  |  |
| Oral Face |  |  |  |  |  |  |  |  |  |  |
| Ciliated Band |  |  |  |  |  |  |  |  |  |  |
| Apical Plate |  |  |  |  |  |  |  |  |  |  |

Predicted interaction: SPU_010424 (OtxA) → SPU_003084 (Gpc6)

Absolute confidence value: 0.093945944

*No spatial expression available for SPU_003084 (Gpc6)

Result: missing complete spatial expression data

|  | **Node1: OtxA** | **Node2: Gpc6*** | **Both expressed** |
| --- | --- | --- | --- |
| Weak expression |  |  |  |
| Expressed |  |  |  |

|  | **0hr** | **6hr** | **9hr** | **12hr** | **15hr** | **18hr** | **21hr** | **24hr** | **27hr** | **30hr** |
| --- | --- | --- | --- | --- | --- | --- | --- | --- | --- | --- |
| Egg |  |  |  |  |  |  |  |  |  |  |
| Small Micromere |  |  |  |  |  |  |  |  |  |  |
| Skel. Micromere |  |  |  |  |  |  |  |  |  |  |
| Macromere |  |  |  |  |  |  |  |  |  |  |
| V2 |  |  |  |  |  |  |  |  |  |  |
| V2 Meso A |  |  |  |  |  |  |  |  |  |  |
| V2 Meso O |  |  |  |  |  |  |  |  |  |  |
| V2 Endo |  |  |  |  |  |  |  |  |  |  |
| V1 |  |  |  |  |  |  |  |  |  |  |
| V1 Endo A |  |  |  |  |  |  |  |  |  |  |
| V1 Endo O |  |  |  |  |  |  |  |  |  |  |
| V1 Ecto A |  |  |  |  |  |  |  |  |  |  |
| V1 Ecto O |  |  |  |  |  |  |  |  |  |  |
| Mesomere |  |  |  |  |  |  |  |  |  |  |
| Ecto A |  |  |  |  |  |  |  |  |  |  |
| Ecto O |  |  |  |  |  |  |  |  |  |  |
| Stomodeum |  |  |  |  |  |  |  |  |  |  |
| Oral Face |  |  |  |  |  |  |  |  |  |  |
| Ciliated Band |  |  |  |  |  |  |  |  |  |  |
| Apical Plate |  |  |  |  |  |  |  |  |  |  |

Predicted interaction: SPU_010424 (OtxA) → SPU_004287 (Smad4)

Absolute confidence value: 0.091939819

*Spatial Expression for SPU_004287 (Smad4) is unavailable before 36hours

Result: missing complete spatial expression data

|  | **Node1: OtxA** | **Node2: Smad4*** | **Both expressed** |
| --- | --- | --- | --- |
| Weak expression |  |  |  |
| Expressed |  |  |  |

|  | **0hr** | **6hr** | **9hr** | **12hr** | **15hr** | **18hr** | **21hr** | **24hr** | **27hr** | **30hr** |
| --- | --- | --- | --- | --- | --- | --- | --- | --- | --- | --- |
| Egg |  |  |  |  |  |  |  |  |  |  |
| Small Micromere |  |  |  |  |  |  |  |  |  |  |
| Skel. Micromere |  |  |  |  |  |  |  |  |  |  |
| Macromere |  |  |  |  |  |  |  |  |  |  |
| V2 |  |  |  |  |  |  |  |  |  |  |
| V2 Meso A |  |  |  |  |  |  |  |  |  |  |
| V2 Meso O |  |  |  |  |  |  |  |  |  |  |
| V2 Endo |  |  |  |  |  |  |  |  |  |  |
| V1 |  |  |  |  |  |  |  |  |  |  |
| V1 Endo A |  |  |  |  |  |  |  |  |  |  |
| V1 Endo O |  |  |  |  |  |  |  |  |  |  |
| V1 Ecto A |  |  |  |  |  |  |  |  |  |  |
| V1 Ecto O |  |  |  |  |  |  |  |  |  |  |
| Mesomere |  |  |  |  |  |  |  |  |  |  |
| Ecto A |  |  |  |  |  |  |  |  |  |  |
| Ecto O |  |  |  |  |  |  |  |  |  |  |
| Stomodeum |  |  |  |  |  |  |  |  |  |  |
| Oral Face |  |  |  |  |  |  |  |  |  |  |
| Ciliated Band |  |  |  |  |  |  |  |  |  |  |
| Apical Plate |  |  |  |  |  |  |  |  |  |  |

Predicted interaction: SPU_002631 (Hox11/13b) → SPU_002129 (Not)

Absolute confidence value: 0.064496756

Result: overlap

|  | **Node1: Hox11/13b** | **Node2: Not** | **Both expressed** |
| --- | --- | --- | --- |
| Weak expression |  |  |  |
| Expressed |  |  |  |

|  | **0hr** | **6hr** | **9hr** | **12hr** | **15hr** | **18hr** | **21hr** | **24hr** | **27hr** | **30hr** |
| --- | --- | --- | --- | --- | --- | --- | --- | --- | --- | --- |
| Egg |  |  |  |  |  |  |  |  |  |  |
| Small Micromere |  |  |  |  |  |  |  |  |  |  |
| Skel. Micromere |  |  |  |  |  |  |  |  |  |  |
| Macromere |  |  |  |  |  |  |  |  |  |  |
| V2 |  |  |  |  |  |  |  |  |  |  |
| V2 Meso A |  |  |  |  |  |  |  |  |  |  |
| V2 Meso O |  |  |  |  |  |  |  |  |  |  |
| V2 Endo |  |  |  |  |  |  |  |  |  |  |
| V1 |  |  |  |  |  |  |  |  |  |  |
| V1 Endo A |  |  |  |  |  |  |  |  |  |  |
| V1 Endo O |  |  |  |  |  |  |  |  |  |  |
| V1 Ecto A |  |  |  |  |  |  |  |  |  |  |
| V1 Ecto O |  |  |  |  |  |  |  |  |  |  |
| Mesomere |  |  |  |  |  |  |  |  |  |  |
| Ecto A |  |  |  |  |  |  |  |  |  |  |
| Ecto O |  |  |  |  |  |  |  |  |  |  |
| Stomodeum |  |  |  |  |  |  |  |  |  |  |
| Oral Face |  |  |  |  |  |  |  |  |  |  |
| Ciliated Band |  |  |  |  |  |  |  |  |  |  |
| Apical Plate |  |  |  |  |  |  |  |  |  |  |

Predicted interaction: SPU_002631 (Hox11/13b) → SPU_006803 (Creb3)

Absolute confidence value: 0.061841677

*No spatial expression data available for SPU_006803 (Creb3)

Result: missing complete spatial expression data

|  | **Node1: Hox11/13b** | **Node2: Creb3*** | **Both expressed** |
| --- | --- | --- | --- |
| Weak expression |  |  |  |
| Expressed |  |  |  |

|  | **0hr** | **6hr** | **9hr** | **12hr** | **15hr** | **18hr** | **21hr** | **24hr** | **27hr** | **30hr** |
| --- | --- | --- | --- | --- | --- | --- | --- | --- | --- | --- |
| Egg |  |  |  |  |  |  |  |  |  |  |
| Small Micromere |  |  |  |  |  |  |  |  |  |  |
| Skel. Micromere |  |  |  |  |  |  |  |  |  |  |
| Macromere |  |  |  |  |  |  |  |  |  |  |
| V2 |  |  |  |  |  |  |  |  |  |  |
| V2 Meso A |  |  |  |  |  |  |  |  |  |  |
| V2 Meso O |  |  |  |  |  |  |  |  |  |  |
| V2 Endo |  |  |  |  |  |  |  |  |  |  |
| V1 |  |  |  |  |  |  |  |  |  |  |
| V1 Endo A |  |  |  |  |  |  |  |  |  |  |
| V1 Endo O |  |  |  |  |  |  |  |  |  |  |
| V1 Ecto A |  |  |  |  |  |  |  |  |  |  |
| V1 Ecto O |  |  |  |  |  |  |  |  |  |  |
| Mesomere |  |  |  |  |  |  |  |  |  |  |
| Ecto A |  |  |  |  |  |  |  |  |  |  |
| Ecto O |  |  |  |  |  |  |  |  |  |  |
| Stomodeum |  |  |  |  |  |  |  |  |  |  |
| Oral Face |  |  |  |  |  |  |  |  |  |  |
| Ciliated Band |  |  |  |  |  |  |  |  |  |  |
| Apical Plate |  |  |  |  |  |  |  |  |  |  |

Predicted interaction: SPU_002631 (Hox11/13b) → SPU_011583 (Z421)

Absolute confidence value: 0.059092198

*No spatial expression data available for SPU_011583 (Z421)

Result: missing complete spatial expression data

|  | **Node1: Hox11/13b** | **Node2: Z421*** | **Both expressed** |
| --- | --- | --- | --- |
| Weak expression |  |  |  |
| Expressed |  |  |  |

|  | **0hr** | **6hr** | **9hr** | **12hr** | **15hr** | **18hr** | **21hr** | **24hr** | **27hr** | **30hr** |
| --- | --- | --- | --- | --- | --- | --- | --- | --- | --- | --- |
| Egg |  |  |  |  |  |  |  |  |  |  |
| Small Micromere |  |  |  |  |  |  |  |  |  |  |
| Skel. Micromere |  |  |  |  |  |  |  |  |  |  |
| Macromere |  |  |  |  |  |  |  |  |  |  |
| V2 |  |  |  |  |  |  |  |  |  |  |
| V2 Meso A |  |  |  |  |  |  |  |  |  |  |
| V2 Meso O |  |  |  |  |  |  |  |  |  |  |
| V2 Endo |  |  |  |  |  |  |  |  |  |  |
| V1 |  |  |  |  |  |  |  |  |  |  |
| V1 Endo A |  |  |  |  |  |  |  |  |  |  |
| V1 Endo O |  |  |  |  |  |  |  |  |  |  |
| V1 Ecto A |  |  |  |  |  |  |  |  |  |  |
| V1 Ecto O |  |  |  |  |  |  |  |  |  |  |
| Mesomere |  |  |  |  |  |  |  |  |  |  |
| Ecto A |  |  |  |  |  |  |  |  |  |  |
| Ecto O |  |  |  |  |  |  |  |  |  |  |
| Stomodeum |  |  |  |  |  |  |  |  |  |  |
| Oral Face |  |  |  |  |  |  |  |  |  |  |
| Ciliated Band |  |  |  |  |  |  |  |  |  |  |
| Apical Plate |  |  |  |  |  |  |  |  |  |  |

Predicted interaction: SPU_008196 (Hnf1a/b) → SPU_013140 (Ron)

Absolute confidence value: 0.04026179

*No spatial expression available for SPU_013140 (Ron)

Result: missing complete spatial expression data

|  | **Node1: Hnf1a/b** | **Node2: Ron*** | **Both expressed** |
| --- | --- | --- | --- |
| Weak expression |  |  |  |
| Expressed |  |  |  |

|  | **0hr** | **6hr** | **9hr** | **12hr** | **15hr** | **18hr** | **21hr** | **24hr** | **27hr** | **30hr** |
| --- | --- | --- | --- | --- | --- | --- | --- | --- | --- | --- |
| Egg |  |  |  |  |  |  |  |  |  |  |
| Small Micromere |  |  |  |  |  |  |  |  |  |  |
| Skel. Micromere |  |  |  |  |  |  |  |  |  |  |
| Macromere |  |  |  |  |  |  |  |  |  |  |
| V2 |  |  |  |  |  |  |  |  |  |  |
| V2 Meso A |  |  |  |  |  |  |  |  |  |  |
| V2 Meso O |  |  |  |  |  |  |  |  |  |  |
| V2 Endo |  |  |  |  |  |  |  |  |  |  |
| V1 |  |  |  |  |  |  |  |  |  |  |
| V1 Endo A |  |  |  |  |  |  |  |  |  |  |
| V1 Endo O |  |  |  |  |  |  |  |  |  |  |
| V1 Ecto A |  |  |  |  |  |  |  |  |  |  |
| V1 Ecto O |  |  |  |  |  |  |  |  |  |  |
| Mesomere |  |  |  |  |  |  |  |  |  |  |
| Ecto A |  |  |  |  |  |  |  |  |  |  |
| Ecto O |  |  |  |  |  |  |  |  |  |  |
| Stomodeum |  |  |  |  |  |  |  |  |  |  |
| Oral Face |  |  |  |  |  |  |  |  |  |  |
| Ciliated Band |  |  |  |  |  |  |  |  |  |  |
| Apical Plate |  |  |  |  |  |  |  |  |  |  |

Predicted interaction: SPU_008196 (Hnf1a/b) → SPU_010305 (Hnf1_1)

Absolute confidence value: 0.039554829

Result: overlap due to indistinguishable spatial expression

**Two isoforms of the same gene, with different SPU IDs and different WHL IDs from the transcriptome. Their spatial expression hasn’t been distinguished, so this could represent an auto-interaction.

|  | **Node1: Hnf1a/b**** | **Node2: Hnf1_1**** | **Both expressed** |
| --- | --- | --- | --- |
| Weak expression |  |  |  |
| Expressed |  |  |  |

|  | **0hr** | **6hr** | **9hr** | **12hr** | **15hr** | **18hr** | **21hr** | **24hr** | **27hr** | **30hr** |
| --- | --- | --- | --- | --- | --- | --- | --- | --- | --- | --- |
| Egg |  |  |  |  |  |  |  |  |  |  |
| Small Micromere |  |  |  |  |  |  |  |  |  |  |
| Skel. Micromere |  |  |  |  |  |  |  |  |  |  |
| Macromere |  |  |  |  |  |  |  |  |  |  |
| V2 |  |  |  |  |  |  |  |  |  |  |
| V2 Meso A |  |  |  |  |  |  |  |  |  |  |
| V2 Meso O |  |  |  |  |  |  |  |  |  |  |
| V2 Endo |  |  |  |  |  |  |  |  |  |  |
| V1 |  |  |  |  |  |  |  |  |  |  |
| V1 Endo A |  |  |  |  |  |  |  |  |  |  |
| V1 Endo O |  |  |  |  |  |  |  |  |  |  |
| V1 Ecto A |  |  |  |  |  |  |  |  |  |  |
| V1 Ecto O |  |  |  |  |  |  |  |  |  |  |
| Mesomere |  |  |  |  |  |  |  |  |  |  |
| Ecto A |  |  |  |  |  |  |  |  |  |  |
| Ecto O |  |  |  |  |  |  |  |  |  |  |
| Stomodeum |  |  |  |  |  |  |  |  |  |  |
| Oral Face |  |  |  |  |  |  |  |  |  |  |
| Ciliated Band |  |  |  |  |  |  |  |  |  |  |
| Apical Plate |  |  |  |  |  |  |  |  |  |  |

Predicted interaction: SPU_008196 (Hnf1a/b) → SPU_004287 (Sp-Smad4)

Absolute confidence value: 0.038424388

*spatial expression for SPU_004287 (Smad4) is unavailable before 36hours

Result: missing complete spatial expression data

|  | **Node1: Hnf1a/b** | **Node2: Smad4*** | **Both expressed** |
| --- | --- | --- | --- |
| Weak expression |  |  |  |
| Expressed |  |  |  |

|  | **0hr** | **6hr** | **9hr** | **12hr** | **15hr** | **18hr** | **21hr** | **24hr** | **27hr** | **30hr** |
| --- | --- | --- | --- | --- | --- | --- | --- | --- | --- | --- |
| Egg |  |  |  |  |  |  |  |  |  |  |
| Small Micromere |  |  |  |  |  |  |  |  |  |  |
| Skel. Micromere |  |  |  |  |  |  |  |  |  |  |
| Macromere |  |  |  |  |  |  |  |  |  |  |
| V2 |  |  |  |  |  |  |  |  |  |  |
| V2 Meso A |  |  |  |  |  |  |  |  |  |  |
| V2 Meso O |  |  |  |  |  |  |  |  |  |  |
| V2 Endo |  |  |  |  |  |  |  |  |  |  |
| V1 |  |  |  |  |  |  |  |  |  |  |
| V1 Endo A |  |  |  |  |  |  |  |  |  |  |
| V1 Endo O |  |  |  |  |  |  |  |  |  |  |
| V1 Ecto A |  |  |  |  |  |  |  |  |  |  |
| V1 Ecto O |  |  |  |  |  |  |  |  |  |  |
| Mesomere |  |  |  |  |  |  |  |  |  |  |
| Ecto A |  |  |  |  |  |  |  |  |  |  |
| Ecto O |  |  |  |  |  |  |  |  |  |  |
| Stomodeum |  |  |  |  |  |  |  |  |  |  |
| Oral Face |  |  |  |  |  |  |  |  |  |  |
| Ciliated Band |  |  |  |  |  |  |  |  |  |  |
| Apical Plate |  |  |  |  |  |  |  |  |  |  |

Predicted interaction: SPU_013569 (Lmo2) → SPU_005572 (Hmg2)

Absolute confidence value: 0.640747321

*No spatial expression available for SPU_013569 (Lmo2) or SPU_005572 (Hmg2)

Result: missing complete spatial expression data

|  | **Node1: Lmo2*** | **Node2: Hmg2*** | **Both expressed** |
| --- | --- | --- | --- |
| Weak expression |  |  |  |
| Expressed |  |  |  |

|  | **0hr** | **6hr** | **9hr** | **12hr** | **15hr** | **18hr** | **21hr** | **24hr** | **27hr** | **30hr** |
| --- | --- | --- | --- | --- | --- | --- | --- | --- | --- | --- |
| Egg |  |  |  |  |  |  |  |  |  |  |
| Small Micromere |  |  |  |  |  |  |  |  |  |  |
| Skel. Micromere |  |  |  |  |  |  |  |  |  |  |
| Macromere |  |  |  |  |  |  |  |  |  |  |
| V2 |  |  |  |  |  |  |  |  |  |  |
| V2 Meso A |  |  |  |  |  |  |  |  |  |  |
| V2 Meso O |  |  |  |  |  |  |  |  |  |  |
| V2 Endo |  |  |  |  |  |  |  |  |  |  |
| V1 |  |  |  |  |  |  |  |  |  |  |
| V1 Endo A |  |  |  |  |  |  |  |  |  |  |
| V1 Endo O |  |  |  |  |  |  |  |  |  |  |
| V1 Ecto A |  |  |  |  |  |  |  |  |  |  |
| V1 Ecto O |  |  |  |  |  |  |  |  |  |  |
| Mesomere |  |  |  |  |  |  |  |  |  |  |
| Ecto A |  |  |  |  |  |  |  |  |  |  |
| Ecto O |  |  |  |  |  |  |  |  |  |  |
| Stomodeum |  |  |  |  |  |  |  |  |  |  |
| Oral Face |  |  |  |  |  |  |  |  |  |  |
| Ciliated Band |  |  |  |  |  |  |  |  |  |  |
| Apical Plate |  |  |  |  |  |  |  |  |  |  |

Predicted interaction: SPU_021557 (Gapb) → SPU_005572 (Hmg2)

Absolute confidence value: 0.634676143

*spatial expression is missing for SPU_021557 (Gapb) or SPU_005572 (Hmg2)

Result: missing complete spatial expression data

|  | **Node1: Gapb*** | **Node2: Gene2*** | **Both expressed** |
| --- | --- | --- | --- |
| Weak expression |  |  |  |
| Expressed |  |  |  |

|  | **0hr** | **6hr** | **9hr** | **12hr** | **15hr** | **18hr** | **21hr** | **24hr** | **27hr** | **30hr** |
| --- | --- | --- | --- | --- | --- | --- | --- | --- | --- | --- |
| Egg |  |  |  |  |  |  |  |  |  |  |
| Small Micromere |  |  |  |  |  |  |  |  |  |  |
| Skel. Micromere |  |  |  |  |  |  |  |  |  |  |
| Macromere |  |  |  |  |  |  |  |  |  |  |
| V2 |  |  |  |  |  |  |  |  |  |  |
| V2 Meso A |  |  |  |  |  |  |  |  |  |  |
| V2 Meso O |  |  |  |  |  |  |  |  |  |  |
| V2 Endo |  |  |  |  |  |  |  |  |  |  |
| V1 |  |  |  |  |  |  |  |  |  |  |
| V1 Endo A |  |  |  |  |  |  |  |  |  |  |
| V1 Endo O |  |  |  |  |  |  |  |  |  |  |
| V1 Ecto A |  |  |  |  |  |  |  |  |  |  |
| V1 Ecto O |  |  |  |  |  |  |  |  |  |  |
| Mesomere |  |  |  |  |  |  |  |  |  |  |
| Ecto A |  |  |  |  |  |  |  |  |  |  |
| Ecto O |  |  |  |  |  |  |  |  |  |  |
| Stomodeum |  |  |  |  |  |  |  |  |  |  |
| Oral Face |  |  |  |  |  |  |  |  |  |  |
| Ciliated Band |  |  |  |  |  |  |  |  |  |  |
| Apical Plate |  |  |  |  |  |  |  |  |  |  |

Predicted interaction: SPU_011635 (FoxJ2_1) → SPU_009155 (Beta-catenin)

Absolute confidence value: 0.633262552

Result: overlap

|  | **Node1: FoxJ2_1** | **Node2: Beta-catenin** | **Both expressed** |
| --- | --- | --- | --- |
| Weak expression |  |  |  |
| Expressed |  |  |  |

|  | **0hr** | **6hr** | **9hr** | **12hr** | **15hr** | **18hr** | **21hr** | **24hr** | **27hr** | **30hr** |
| --- | --- | --- | --- | --- | --- | --- | --- | --- | --- | --- |
| Egg |  |  |  |  |  |  |  |  |  |  |
| Small Micromere |  |  |  |  |  |  |  |  |  |  |
| Skel. Micromere |  |  |  |  |  |  |  |  |  |  |
| Macromere |  |  |  |  |  |  |  |  |  |  |
| V2 |  |  |  |  |  |  |  |  |  |  |
| V2 Meso A |  |  |  |  |  |  |  |  |  |  |
| V2 Meso O |  |  |  |  |  |  |  |  |  |  |
| V2 Endo |  |  |  |  |  |  |  |  |  |  |
| V1 |  |  |  |  |  |  |  |  |  |  |
| V1 Endo A |  |  |  |  |  |  |  |  |  |  |
| V1 Endo O |  |  |  |  |  |  |  |  |  |  |
| V1 Ecto A |  |  |  |  |  |  |  |  |  |  |
| V1 Ecto O |  |  |  |  |  |  |  |  |  |  |
| Mesomere |  |  |  |  |  |  |  |  |  |  |
| Ecto A |  |  |  |  |  |  |  |  |  |  |
| Ecto O |  |  |  |  |  |  |  |  |  |  |
| Stomodeum |  |  |  |  |  |  |  |  |  |  |
| Oral Face |  |  |  |  |  |  |  |  |  |  |
| Ciliated Band |  |  |  |  |  |  |  |  |  |  |
| Apical Plate |  |  |  |  |  |  |  |  |  |  |

Predicted interaction: SPU_023090 (Prickle) → SPU_002874 (Ets1/2)

Absolute confidence value: 0.63180003

*No spatial expression available for SPU_023090 (Prickle)

Result: missing complete spatial expression data

|  | **Node1: Prickle*** | **Node2: Ets1/2** | **Both expressed** |
| --- | --- | --- | --- |
| Weak expression |  |  |  |
| Expressed |  |  |  |

|  | **0hr** | **6hr** | **9hr** | **12hr** | **15hr** | **18hr** | **21hr** | **24hr** | **27hr** | **30hr** |
| --- | --- | --- | --- | --- | --- | --- | --- | --- | --- | --- |
| Egg |  |  |  |  |  |  |  |  |  |  |
| Small Micromere |  |  |  |  |  |  |  |  |  |  |
| Skel. Micromere |  |  |  |  |  |  |  |  |  |  |
| Macromere |  |  |  |  |  |  |  |  |  |  |
| V2 |  |  |  |  |  |  |  |  |  |  |
| V2 Meso A |  |  |  |  |  |  |  |  |  |  |
| V2 Meso O |  |  |  |  |  |  |  |  |  |  |
| V2 Endo |  |  |  |  |  |  |  |  |  |  |
| V1 |  |  |  |  |  |  |  |  |  |  |
| V1 Endo A |  |  |  |  |  |  |  |  |  |  |
| V1 Endo O |  |  |  |  |  |  |  |  |  |  |
| V1 Ecto A |  |  |  |  |  |  |  |  |  |  |
| V1 Ecto O |  |  |  |  |  |  |  |  |  |  |
| Mesomere |  |  |  |  |  |  |  |  |  |  |
| Ecto A |  |  |  |  |  |  |  |  |  |  |
| Ecto O |  |  |  |  |  |  |  |  |  |  |
| Stomodeum |  |  |  |  |  |  |  |  |  |  |
| Oral Face |  |  |  |  |  |  |  |  |  |  |
| Ciliated Band |  |  |  |  |  |  |  |  |  |  |
| Apical Plate |  |  |  |  |  |  |  |  |  |  |

Predicted interaction: SPU_018056 (Homeo1) → SPU_002874 (Ets1/2)

Absolute confidence value: 0.631211334

*No spatial expression available for SPU_018056 (Homeo1)

Result: missing complete spatial expression data

|  | **Node1: Homeo1*** | **Node2: Ets1/2** | **Both expressed** |
| --- | --- | --- | --- |
| Weak expression |  |  |  |
| Expressed |  |  |  |

|  | **0hr** | **6hr** | **9hr** | **12hr** | **15hr** | **18hr** | **21hr** | **24hr** | **27hr** | **30hr** |
| --- | --- | --- | --- | --- | --- | --- | --- | --- | --- | --- |
| Egg |  |  |  |  |  |  |  |  |  |  |
| Small Micromere |  |  |  |  |  |  |  |  |  |  |
| Skel. Micromere |  |  |  |  |  |  |  |  |  |  |
| Macromere |  |  |  |  |  |  |  |  |  |  |
| V2 |  |  |  |  |  |  |  |  |  |  |
| V2 Meso A |  |  |  |  |  |  |  |  |  |  |
| V2 Meso O |  |  |  |  |  |  |  |  |  |  |
| V2 Endo |  |  |  |  |  |  |  |  |  |  |
| V1 |  |  |  |  |  |  |  |  |  |  |
| V1 Endo A |  |  |  |  |  |  |  |  |  |  |
| V1 Endo O |  |  |  |  |  |  |  |  |  |  |
| V1 Ecto A |  |  |  |  |  |  |  |  |  |  |
| V1 Ecto O |  |  |  |  |  |  |  |  |  |  |
| Mesomere |  |  |  |  |  |  |  |  |  |  |
| Ecto A |  |  |  |  |  |  |  |  |  |  |
| Ecto O |  |  |  |  |  |  |  |  |  |  |
| Stomodeum |  |  |  |  |  |  |  |  |  |  |
| Oral Face |  |  |  |  |  |  |  |  |  |  |
| Ciliated Band |  |  |  |  |  |  |  |  |  |  |
| Apical Plate |  |  |  |  |  |  |  |  |  |  |

Predicted interaction: SPU_011189 (Z141) → SPU_009155 (Beta-catenin)

Absolute confidence value: 0.629876338

Expression for SPU_011189 (Z141) reported in PMID: 16997293

|  | **Node1: Gene1** | **Node2: Gene2** | **Both expressed** |
| --- | --- | --- | --- |
| Weak expression |  |  |  |
| Expressed |  |  |  |

|  | **0hr** | **6hr** | **9hr** | **12hr** | **15hr** | **18hr** | **21hr** | **24hr** | **27hr** | **30hr** |
| --- | --- | --- | --- | --- | --- | --- | --- | --- | --- | --- |
| Egg |  |  |  |  |  |  |  |  |  |  |
| Small Micromere |  |  |  |  |  |  |  |  |  |  |
| Skel. Micromere |  |  |  |  |  |  |  |  |  |  |
| Macromere |  |  |  |  |  |  |  |  |  |  |
| V2 |  |  |  |  |  |  |  |  |  |  |
| V2 Meso A |  |  |  |  |  |  |  |  |  |  |
| V2 Meso O |  |  |  |  |  |  |  |  |  |  |
| V2 Endo |  |  |  |  |  |  |  |  |  |  |
| V1 |  |  |  |  |  |  |  |  |  |  |
| V1 Endo A |  |  |  |  |  |  |  |  |  |  |
| V1 Endo O |  |  |  |  |  |  |  |  |  |  |
| V1 Ecto A |  |  |  |  |  |  |  |  |  |  |
| V1 Ecto O |  |  |  |  |  |  |  |  |  |  |
| Mesomere |  |  |  |  |  |  |  |  |  |  |
| Ecto A |  |  |  |  |  |  |  |  |  |  |
| Ecto O |  |  |  |  |  |  |  |  |  |  |
| Stomodeum |  |  |  |  |  |  |  |  |  |  |
| Oral Face |  |  |  |  |  |  |  |  |  |  |
| Ciliated Band |  |  |  |  |  |  |  |  |  |  |
| Apical Plate |  |  |  |  |  |  |  |  |  |  |

Predicted interaction: SPU_005358 (Crem) → SPU_021608 (HesC)

Absolute confidence value: 0.628806788

*No spatial expression available for SPU_005358 (Crem)

Result: missing complete spatial expression data

|  | **Node1: Crem*** | **Node2: HesC** | **Both expressed** |
| --- | --- | --- | --- |
| Weak expression |  |  |  |
| Expressed |  |  |  |

|  | **0hr** | **6hr** | **9hr** | **12hr** | **15hr** | **18hr** | **21hr** | **24hr** | **27hr** | **30hr** |
| --- | --- | --- | --- | --- | --- | --- | --- | --- | --- | --- |
| Egg |  |  |  |  |  |  |  |  |  |  |
| Small Micromere |  |  |  |  |  |  |  |  |  |  |
| Skel. Micromere |  |  |  |  |  |  |  |  |  |  |
| Macromere |  |  |  |  |  |  |  |  |  |  |
| V2 |  |  |  |  |  |  |  |  |  |  |
| V2 Meso A |  |  |  |  |  |  |  |  |  |  |
| V2 Meso O |  |  |  |  |  |  |  |  |  |  |
| V2 Endo |  |  |  |  |  |  |  |  |  |  |
| V1 |  |  |  |  |  |  |  |  |  |  |
| V1 Endo A |  |  |  |  |  |  |  |  |  |  |
| V1 Endo O |  |  |  |  |  |  |  |  |  |  |
| V1 Ecto A |  |  |  |  |  |  |  |  |  |  |
| V1 Ecto O |  |  |  |  |  |  |  |  |  |  |
| Mesomere |  |  |  |  |  |  |  |  |  |  |
| Ecto A |  |  |  |  |  |  |  |  |  |  |
| Ecto O |  |  |  |  |  |  |  |  |  |  |
| Stomodeum |  |  |  |  |  |  |  |  |  |  |
| Oral Face |  |  |  |  |  |  |  |  |  |  |
| Ciliated Band |  |  |  |  |  |  |  |  |  |  |
| Apical Plate |  |  |  |  |  |  |  |  |  |  |
