# Supplementary material for: Developmental gene regulatory network connections predicted by machine learning from gene expression data alone
Source: PLoS One. 2021 Dec 28;16(12):e0261926. doi: 10.1371/journal.pone.0261926 (PMC8714117; doi:10.1371/journal.pone.0261926)
Supplement: S7 File — The spatial expression comparison it its entirely for the Bottom-50 interactions, as sorted by absolute PEAK confidence scores. Starts with a summary table. For each interaction a separate page displays the spatial expression for each gene in each interaction presented as a color-coded co-expression matrix. (DOCX) [file pone.0261926.s007.docx]

Table summarizing spatial expression analysis of the Bottom50 new PEAK predictions followed by a visualization of expression domains for each interaction. Predictions that matched a known interaction are the rows with a green background. Known spatial expression patterns from 0-30hrs were extracted using the common spatial expression matrix found in the Legacy Echinobase gene pages, in the data section within the Biotapestry Gene Regulatory Networks, or otherwise noted as based on in situ analysis in a particular manuscript. Spatial expression domains for each gene in the predicted interaction were compared and marked as displaying “overlap,” or “non-overlapping” expression. If one or both genes did not have spatial expression data available in the database, the interaction was marked as “missing expression.”

| **Number** | **Node1 ID** | **Node 1 Name** | **Node2 ID** | **Node 2 Name** | **Peak Confidence Score** | **Result** |
| --- | --- | --- | --- | --- | --- | --- |
| 1 | SPU_015719 | Sp-FoxL1 | SPU_020803 | Sp-Trk | 0.0000006930726419 | missing exp |
| 2 | SPU_020565 | Sp-Msxl | SPU_004024 | Sp-Pka_1 | 0.0000008316871703 | missing exp |
| 3 | SPU_019002 | Sp-FoxQ2_1 | SPU_028479 | Sp-Tel | 0.000002633676039 | Non-overlapping |
| 4 | SPU_022242 | Sp-Smadip | SPU_013576 | Sp-Z96 | 0.00000429705038 | missing exp |
| 5 | SPU_006803 | Sp-Creb3l3 | SPU_011270 | Sp-Z300 | 0.000004851508493 | missing exp |
| 6 | SPU_013689 | Sp-L3mbt_1 | SPU_007190 | Sp-Z185 | 0.000006792111891 | missing exp |
| 7 | SPU_018391 | Sp-Tbx20 | SPU_011174 | Sp-Nfe2 | 0.000007207955476 | missing exp |
| 8 | SPU_006991 | Sp-Lim1 | SPU_017750 | Sp-Z159 | 0.000007623799061 | missing exp |
| 9 | SPU_006991 | Sp-Lim1 | SPU_014836 | Sp-Cp2 | 0.000008178257175 | missing exp |
| 10 | SPU_024918 | Sp-NeuroD | SPU_017404 | Sp-Nr1h6a | 0.000009564402458 | missing exp |
| - | SPU_006421 | No Gene found | SPU_017404 | Sp-Nr1h6a | 0.00001552482718 | - |
| 11 | SPU_027235 | Sp-Blimp1a/b | SPU_009079 | Sp-FgfrL5 | 0.00001788127416 | Missing exp |
| 12 | SPU_017379 | Sp-Six1/2 | SPU_001415 | Sp-Z200 | 0.0000185743468 | Missing exp |
| 13 | SPU_008117 | Sp-Shr2 | SPU_017404 | Sp-Nr1h6a | 0.00002009910662 | Missing exp |
| 14 | SPU_025590 | Sp-FoxM | SPU_015199 | Sp-Z185_1 | 0.00002023772114 | Missing exp |
| 15 | SPU_006808 | Sp-Af9 | SPU_026763 | Sp-Scmh1 | 0.00002481200058 | Missing exp |
| 16 | SPU_004287 | Sp-Smad4 | SPU_014687 | Sp-Z310_1 | 0.00002869320738 | Missing exp |
| 17 | SPU_006312 | Sp-Dp1 | SPU_002554 | Sp-Phf20L | 0.00002910905096 | Missing exp |
| 18 | SPU_004287 | Sp-Smad4 | SPU_013810 | Sp-Ptpn6/11 | 0.00002938628002 | Missing exp |
| 19 | SPU_007644 | Sp-FoxJ2 | SPU_027708 | Sp-Z491 | 0.00003007935266 | Missing exp |
| 20 | SPU_004414 | Sp-Hlf | SPU_012111 | Sp-Z53 | 0.00003063381077 | Missing exp |
| 21 | SPU_012008 | Sp-Myor2 | SPU_014461 | Sp-Pitx1 | 0.00003257441417 | Missing exp |
| 22 | SPU_002320 | Sp-FoxN1/4 | SPU_021666 | Sp-Dok | 0.00003299025776 | Missing exp |
| 23 | SPU_003166 | Sp-Myc | SPU_020330 | Sp-Grem | 0.00003312887228 | Missing exp |
| 24 | SPU_019332 | Sp-Ppar1 | SPU_013305 | Sp-Grf | 0.00003326748681 | Missing exp |
| 25 | SPU_004148 | Sp-Krl | SPU_000806 | Sp-Cad96Cal | 0.00003354471587 | Missing exp |
| 26 | SPU_012772 | Sp-Klf7 | SPU_000483 | Sp-Z353 | 0.00003562393379 | Missing exp |
| 27 | SPU_026905 | Sp-Atf2 | SPU_004148 | Sp-Krl | 0.00003936652606 | Missing exp |
| 28 | SPU_005358 | Sp-Crem | SPU_013140 | Sp-Ron | 0.00004227743116 | Missing exp |
| 29 | SPU_017379 | Sp-Six1/2 | SPU_015829 | Sp-Pmar1d | 0.0000439408055 | Non-overlapping |
| 30 | SPU_017407 | Sp-Clock | SPU_020646 | Sp-Ror | 0.00004435664908 | Missing exp |
| 31 | SPU_012772 | Sp-Klf7 | SPU_022678 | Sp-RoraL | 0.00004602002342 | Missing exp |
| 32 | SPU_018126 | Sp-Tgif | SPU_003378 | Sp-Plag1L | 0.00004615863795 | Missing exp |
| 33 | SPU_022846 | Sp-FoxABL | SPU_022892 | Sp-Z61 | 0.00004976261569 | Missing exp |
| 34 | SPU_008528 | Sp-Ets4 | SPU_022841 | Sp-Z142 | 0.00004990123022 | Missing exp |
| 35 | SPU_006991 | Sp-Lim1 | SPU_012877 | Sp-Kif27 | 0.00005142599003 | Missing exp |
| 36 | SPU_011635 | Sp-FoxJ2_1 | SPU_016490 | Sp-Mtf1 | 0.00005530719682 | Missing exp |
| 37 | SPU_000975 | Sp-FoxF | SPU_011202 | Sp-Meis | 0.00005600026947 | Missing exp |
| 38 | SPU_012203 | Sp-Rel | SPU_004551 | Sp-FoxB | 0.00005600026947 | Missing exp |
| 39 | SPU_019332 | Sp-Ppar1 | SPU_008953 | Sp-Talpid3 | 0.00006196069419 | Missing exp |
| 40 | SPU_015374 | Sp-Id | SPU_030139 | Sp-Igf2 | 0.00006196069419 | Missing exp |
| 41 | SPU_003595 | Sp-Cutl | SPU_024953 | Sp-Z34 | 0.00006209930872 | Missing exp |
| 42 | SPU_007981 | Sp-Lmpt | SPU_008747 | Sp-Fused | 0.00006320822494 | Missing exp |
| - | SPU_006421 | No Gene found | SPU_007644 | Sp-FoxJ2 | 0.00006320822494 | - |
| 43 | SPU_014793 | Sp-Spalt | SPU_005522 | Sp-Ift172_3 | 0.00006362406853 | Missing exp |
| 44 | SPU_011635 | Sp-FoxJ2_1 | SPU_014687 | Sp-Z310_1 | 0.00006403991211 | Missing exp |
| 45 | SPU_015828 | Sp-Pmar1c | SPU_022955 | Sp-Z76 | 0.00006556467193 | Missing exp |
| 46 | SPU_011246 | Sp-IrxB | SPU_002428 | Sp-Mib2 | 0.00006722804627 | Missing exp |
| 47 | SPU_026763 | Sp-Scmh1 | SPU_019825 | Sp-Z352 | 0.00006792111891 | Missing exp |
| 48 | SPU_006676 | Sp-FoxA | SPU_027144 | Sp-Pik3ca | 0.00006861419155 | Missing exp |

Predicted interaction: SPU_015719 (Sp-FoxL1) → SPU_020803 (Sp-Trk)

Absolute confidence value: 0.0000006930726419139

*Gene expression pattern not available for SPU_015719 (Sp-FoxL1) or SPU_020803 (Sp-Trk) during 0-30hrs

Result: missing complete spatial expression data

|  | **Node1: Sp-FoxL1*** | **Node2: Sp-Trk*** | **Both expressed** |
| --- | --- | --- | --- |
| Weak expression |  |  |  |
| Expressed |  |  |  |

|  | **0hr** | **6hr** | **9hr** | **12hr** | **15hr** | **18hr** | **21hr** | **24hr** | **27hr** | **30hr** |
| --- | --- | --- | --- | --- | --- | --- | --- | --- | --- | --- |
| Egg |  |  |  |  |  |  |  |  |  |  |
| Small Micromere |  |  |  |  |  |  |  |  |  |  |
| Skel. Micromere |  |  |  |  |  |  |  |  |  |  |
| Macromere |  |  |  |  |  |  |  |  |  |  |
| V2 |  |  |  |  |  |  |  |  |  |  |
| V2 Meso A |  |  |  |  |  |  |  |  |  |  |
| V2 Meso O |  |  |  |  |  |  |  |  |  |  |
| V2 Endo |  |  |  |  |  |  |  |  |  |  |
| V1 |  |  |  |  |  |  |  |  |  |  |
| V1 Endo A |  |  |  |  |  |  |  |  |  |  |
| V1 Endo O |  |  |  |  |  |  |  |  |  |  |
| V1 Ecto A |  |  |  |  |  |  |  |  |  |  |
| V1 Ecto O |  |  |  |  |  |  |  |  |  |  |
| Mesomere |  |  |  |  |  |  |  |  |  |  |
| Ecto A |  |  |  |  |  |  |  |  |  |  |
| Ecto O |  |  |  |  |  |  |  |  |  |  |
| Stomodeum |  |  |  |  |  |  |  |  |  |  |
| Oral Face |  |  |  |  |  |  |  |  |  |  |
| Ciliated Band |  |  |  |  |  |  |  |  |  |  |
| Apical Plate |  |  |  |  |  |  |  |  |  |  |

Predicted interaction: SPU_020565 (Msxl) → SPU_004024 (Pka_1)

Absolute confidence value: 0.00000083168717029668

* Gene expression pattern not available for SPU_020565 (Msxl) or SPU_004024 (Pka_1)

Result: missing complete spatial expression data

|  | **Node1: Msxl*** | **Node2: Pka_1*** | **Both expressed** |
| --- | --- | --- | --- |
| Weak expression |  |  |  |
| Expressed |  |  |  |

|  | **0hr** | **6hr** | **9hr** | **12hr** | **15hr** | **18hr** | **21hr** | **24hr** | **27hr** | **30hr** |
| --- | --- | --- | --- | --- | --- | --- | --- | --- | --- | --- |
| Egg |  |  |  |  |  |  |  |  |  |  |
| Small Micromere |  |  |  |  |  |  |  |  |  |  |
| Skel. Micromere |  |  |  |  |  |  |  |  |  |  |
| Macromere |  |  |  |  |  |  |  |  |  |  |
| V2 |  |  |  |  |  |  |  |  |  |  |
| V2 Meso A |  |  |  |  |  |  |  |  |  |  |
| V2 Meso O |  |  |  |  |  |  |  |  |  |  |
| V2 Endo |  |  |  |  |  |  |  |  |  |  |
| V1 |  |  |  |  |  |  |  |  |  |  |
| V1 Endo A |  |  |  |  |  |  |  |  |  |  |
| V1 Endo O |  |  |  |  |  |  |  |  |  |  |
| V1 Ecto A |  |  |  |  |  |  |  |  |  |  |
| V1 Ecto O |  |  |  |  |  |  |  |  |  |  |
| Mesomere |  |  |  |  |  |  |  |  |  |  |
| Ecto A |  |  |  |  |  |  |  |  |  |  |
| Ecto O |  |  |  |  |  |  |  |  |  |  |
| Stomodeum |  |  |  |  |  |  |  |  |  |  |
| Oral Face |  |  |  |  |  |  |  |  |  |  |
| Ciliated Band |  |  |  |  |  |  |  |  |  |  |
| Apical Plate |  |  |  |  |  |  |  |  |  |  |

Predicted interaction: SPU_019002 (FoxQ2_1) → SPU_028479 (Tel)

Absolute confidence value: 0.000002633676039

**SPU_028479 (Tel) was part of a redundancy in the genome assembly. The SPU_ID for the redundant gene model is SPU_008351, the gene name is Tel_1

Result: non-overlapping

|  | **Node1: FoxQ2_1** | **Node2: Tel_1**** | **Both expressed** |
| --- | --- | --- | --- |
| Weak expression |  |  |  |
| Expressed |  |  |  |

|  | **0hr** | **6hr** | **9hr** | **12hr** | **15hr** | **18hr** | **21hr** | **24hr** | **27hr** | **30hr** |
| --- | --- | --- | --- | --- | --- | --- | --- | --- | --- | --- |
| Egg |  |  |  |  |  |  |  |  |  |  |
| Small Micromere |  |  |  |  |  |  |  |  |  |  |
| Skel. Micromere |  |  |  |  |  |  |  |  |  |  |
| Macromere |  |  |  |  |  |  |  |  |  |  |
| V2 |  |  |  |  |  |  |  |  |  |  |
| V2 Meso A |  |  |  |  |  |  |  |  |  |  |
| V2 Meso O |  |  |  |  |  |  |  |  |  |  |
| V2 Endo |  |  |  |  |  |  |  |  |  |  |
| V1 |  |  |  |  |  |  |  |  |  |  |
| V1 Endo A |  |  |  |  |  |  |  |  |  |  |
| V1 Endo O |  |  |  |  |  |  |  |  |  |  |
| V1 Ecto A |  |  |  |  |  |  |  |  |  |  |
| V1 Ecto O |  |  |  |  |  |  |  |  |  |  |
| Mesomere |  |  |  |  |  |  |  |  |  |  |
| Ecto A |  |  |  |  |  |  |  |  |  |  |
| Ecto O |  |  |  |  |  |  |  |  |  |  |
| Stomodeum |  |  |  |  |  |  |  |  |  |  |
| Oral Face |  |  |  |  |  |  |  |  |  |  |
| Ciliated Band |  |  |  |  |  |  |  |  |  |  |
| Apical Plate |  |  |  |  |  |  |  |  |  |  |

Predicted interaction: SPU_022242 (Smadip) → SPU_013576 (Z96)

Absolute confidence value: 0.00000429705037986618

*Gene expression not available for SPU_022242 (Smadip) or SPU_013576 (Z96)

Result: missing complete spatial expression data

|  | **Node1: Smadip*** | **Node2: Z96*** | **Both expressed** |
| --- | --- | --- | --- |
| Weak expression |  |  |  |
| Expressed |  |  |  |

|  | **0hr** | **6hr** | **9hr** | **12hr** | **15hr** | **18hr** | **21hr** | **24hr** | **27hr** | **30hr** |
| --- | --- | --- | --- | --- | --- | --- | --- | --- | --- | --- |
| Egg |  |  |  |  |  |  |  |  |  |  |
| Small Micromere |  |  |  |  |  |  |  |  |  |  |
| Skel. Micromere |  |  |  |  |  |  |  |  |  |  |
| Macromere |  |  |  |  |  |  |  |  |  |  |
| V2 |  |  |  |  |  |  |  |  |  |  |
| V2 Meso A |  |  |  |  |  |  |  |  |  |  |
| V2 Meso O |  |  |  |  |  |  |  |  |  |  |
| V2 Endo |  |  |  |  |  |  |  |  |  |  |
| V1 |  |  |  |  |  |  |  |  |  |  |
| V1 Endo A |  |  |  |  |  |  |  |  |  |  |
| V1 Endo O |  |  |  |  |  |  |  |  |  |  |
| V1 Ecto A |  |  |  |  |  |  |  |  |  |  |
| V1 Ecto O |  |  |  |  |  |  |  |  |  |  |
| Mesomere |  |  |  |  |  |  |  |  |  |  |
| Ecto A |  |  |  |  |  |  |  |  |  |  |
| Ecto O |  |  |  |  |  |  |  |  |  |  |
| Stomodeum |  |  |  |  |  |  |  |  |  |  |
| Oral Face |  |  |  |  |  |  |  |  |  |  |
| Ciliated Band |  |  |  |  |  |  |  |  |  |  |
| Apical Plate |  |  |  |  |  |  |  |  |  |  |

Predicted interaction: SPU_006803 (Creb3l3) → SPU_011270 (Z300)

Absolute confidence value: 0.0000048515084933973

*Gene expression is not available for SPU_006803 (Creb3l3) or SPU_011270 (Z300)

Result: missing complete spatial expression data

|  | **Node1: Creb3l3*** | **Node2: Z300*** | **Both expressed** |
| --- | --- | --- | --- |
| Weak expression |  |  |  |
| Expressed |  |  |  |

|  | **0hr** | **6hr** | **9hr** | **12hr** | **15hr** | **18hr** | **21hr** | **24hr** | **27hr** | **30hr** |
| --- | --- | --- | --- | --- | --- | --- | --- | --- | --- | --- |
| Egg |  |  |  |  |  |  |  |  |  |  |
| Small Micromere |  |  |  |  |  |  |  |  |  |  |
| Skel. Micromere |  |  |  |  |  |  |  |  |  |  |
| Macromere |  |  |  |  |  |  |  |  |  |  |
| V2 |  |  |  |  |  |  |  |  |  |  |
| V2 Meso A |  |  |  |  |  |  |  |  |  |  |
| V2 Meso O |  |  |  |  |  |  |  |  |  |  |
| V2 Endo |  |  |  |  |  |  |  |  |  |  |
| V1 |  |  |  |  |  |  |  |  |  |  |
| V1 Endo A |  |  |  |  |  |  |  |  |  |  |
| V1 Endo O |  |  |  |  |  |  |  |  |  |  |
| V1 Ecto A |  |  |  |  |  |  |  |  |  |  |
| V1 Ecto O |  |  |  |  |  |  |  |  |  |  |
| Mesomere |  |  |  |  |  |  |  |  |  |  |
| Ecto A |  |  |  |  |  |  |  |  |  |  |
| Ecto O |  |  |  |  |  |  |  |  |  |  |
| Stomodeum |  |  |  |  |  |  |  |  |  |  |
| Oral Face |  |  |  |  |  |  |  |  |  |  |
| Ciliated Band |  |  |  |  |  |  |  |  |  |  |
| Apical Plate |  |  |  |  |  |  |  |  |  |  |

Predicted interaction: SPU_013689 (L3mbt_1) → SPU_007190 (Z185)

Absolute confidence value: 0.00000679211189075622

*Gene expression is not available for SPU_013689 (L3mbt_1) or SPU_007190 (Z185)

Result: missing complete spatial expression data

|  | **Node1: L3mbt_1*** | **Node2: Z185*** | **Both expressed** |
| --- | --- | --- | --- |
| Weak expression |  |  |  |
| Expressed |  |  |  |

|  | **0hr** | **6hr** | **9hr** | **12hr** | **15hr** | **18hr** | **21hr** | **24hr** | **27hr** | **30hr** |
| --- | --- | --- | --- | --- | --- | --- | --- | --- | --- | --- |
| Egg |  |  |  |  |  |  |  |  |  |  |
| Small Micromere |  |  |  |  |  |  |  |  |  |  |
| Skel. Micromere |  |  |  |  |  |  |  |  |  |  |
| Macromere |  |  |  |  |  |  |  |  |  |  |
| V2 |  |  |  |  |  |  |  |  |  |  |
| V2 Meso A |  |  |  |  |  |  |  |  |  |  |
| V2 Meso O |  |  |  |  |  |  |  |  |  |  |
| V2 Endo |  |  |  |  |  |  |  |  |  |  |
| V1 |  |  |  |  |  |  |  |  |  |  |
| V1 Endo A |  |  |  |  |  |  |  |  |  |  |
| V1 Endo O |  |  |  |  |  |  |  |  |  |  |
| V1 Ecto A |  |  |  |  |  |  |  |  |  |  |
| V1 Ecto O |  |  |  |  |  |  |  |  |  |  |
| Mesomere |  |  |  |  |  |  |  |  |  |  |
| Ecto A |  |  |  |  |  |  |  |  |  |  |
| Ecto O |  |  |  |  |  |  |  |  |  |  |
| Stomodeum |  |  |  |  |  |  |  |  |  |  |
| Oral Face |  |  |  |  |  |  |  |  |  |  |
| Ciliated Band |  |  |  |  |  |  |  |  |  |  |
| Apical Plate |  |  |  |  |  |  |  |  |  |  |

Predicted interaction: SPU_018391 (Tbx20) → SPU_011174 (Nfe2)

Absolute confidence value: 0.00000720795547590456

*Gene expression is not available for SPU_018391 (Tbx20)

Result: missing complete spatial expression data

|  | **Node1: Tbx20*** | **Node2: Nfe2** | **Both expressed** |
| --- | --- | --- | --- |
| Weak expression |  |  |  |
| Expressed |  |  |  |

|  | **0hr** | **6hr** | **9hr** | **12hr** | **15hr** | **18hr** | **21hr** | **24hr** | **27hr** | **30hr** |
| --- | --- | --- | --- | --- | --- | --- | --- | --- | --- | --- |
| Egg |  |  |  |  |  |  |  |  |  |  |
| Small Micromere |  |  |  |  |  |  |  |  |  |  |
| Skel. Micromere |  |  |  |  |  |  |  |  |  |  |
| Macromere |  |  |  |  |  |  |  |  |  |  |
| V2 |  |  |  |  |  |  |  |  |  |  |
| V2 Meso A |  |  |  |  |  |  |  |  |  |  |
| V2 Meso O |  |  |  |  |  |  |  |  |  |  |
| V2 Endo |  |  |  |  |  |  |  |  |  |  |
| V1 |  |  |  |  |  |  |  |  |  |  |
| V1 Endo A |  |  |  |  |  |  |  |  |  |  |
| V1 Endo O |  |  |  |  |  |  |  |  |  |  |
| V1 Ecto A |  |  |  |  |  |  |  |  |  |  |
| V1 Ecto O |  |  |  |  |  |  |  |  |  |  |
| Mesomere |  |  |  |  |  |  |  |  |  |  |
| Ecto A |  |  |  |  |  |  |  |  |  |  |
| Ecto O |  |  |  |  |  |  |  |  |  |  |
| Stomodeum |  |  |  |  |  |  |  |  |  |  |
| Oral Face |  |  |  |  |  |  |  |  |  |  |
| Ciliated Band |  |  |  |  |  |  |  |  |  |  |
| Apical Plate |  |  |  |  |  |  |  |  |  |  |

Predicted interaction: SPU_006991 (Lim1) → SPU_017750 (Z159)

Absolute confidence value: 0.0000076237990610529

*Gene expression is not available for SPU_017750 (Z159)

Result: missing complete spatial expression data

|  | **Node1: Lim1** | **Node2: Z159*** | **Both expressed** |
| --- | --- | --- | --- |
| Weak expression |  |  |  |
| Expressed |  |  |  |

|  | **0hr** | **6hr** | **9hr** | **12hr** | **15hr** | **18hr** | **21hr** | **24hr** | **27hr** | **30hr** |
| --- | --- | --- | --- | --- | --- | --- | --- | --- | --- | --- |
| Egg |  |  |  |  |  |  |  |  |  |  |
| Small Micromere |  |  |  |  |  |  |  |  |  |  |
| Skel. Micromere |  |  |  |  |  |  |  |  |  |  |
| Macromere |  |  |  |  |  |  |  |  |  |  |
| V2 |  |  |  |  |  |  |  |  |  |  |
| V2 Meso A |  |  |  |  |  |  |  |  |  |  |
| V2 Meso O |  |  |  |  |  |  |  |  |  |  |
| V2 Endo |  |  |  |  |  |  |  |  |  |  |
| V1 |  |  |  |  |  |  |  |  |  |  |
| V1 Endo A |  |  |  |  |  |  |  |  |  |  |
| V1 Endo O |  |  |  |  |  |  |  |  |  |  |
| V1 Ecto A |  |  |  |  |  |  |  |  |  |  |
| V1 Ecto O |  |  |  |  |  |  |  |  |  |  |
| Mesomere |  |  |  |  |  |  |  |  |  |  |
| Ecto A |  |  |  |  |  |  |  |  |  |  |
| Ecto O |  |  |  |  |  |  |  |  |  |  |
| Stomodeum |  |  |  |  |  |  |  |  |  |  |
| Oral Face |  |  |  |  |  |  |  |  |  |  |
| Ciliated Band |  |  |  |  |  |  |  |  |  |  |
| Apical Plate |  |  |  |  |  |  |  |  |  |  |

Predicted interaction: SPU_006991 (Lim1) → SPU_014836 (Cp2)

Absolute confidence value: 0.00000817825717458402

*Gene expression is not available for SPU_014836 (Cp2)

Result: missing complete spatial expression data

|  | **Node1: Lim1** | **Node2: Cp2*** | **Both expressed** |
| --- | --- | --- | --- |
| Weak expression |  |  |  |
| Expressed |  |  |  |

|  | **0hr** | **6hr** | **9hr** | **12hr** | **15hr** | **18hr** | **21hr** | **24hr** | **27hr** | **30hr** |
| --- | --- | --- | --- | --- | --- | --- | --- | --- | --- | --- |
| Egg |  |  |  |  |  |  |  |  |  |  |
| Small Micromere |  |  |  |  |  |  |  |  |  |  |
| Skel. Micromere |  |  |  |  |  |  |  |  |  |  |
| Macromere |  |  |  |  |  |  |  |  |  |  |
| V2 |  |  |  |  |  |  |  |  |  |  |
| V2 Meso A |  |  |  |  |  |  |  |  |  |  |
| V2 Meso O |  |  |  |  |  |  |  |  |  |  |
| V2 Endo |  |  |  |  |  |  |  |  |  |  |
| V1 |  |  |  |  |  |  |  |  |  |  |
| V1 Endo A |  |  |  |  |  |  |  |  |  |  |
| V1 Endo O |  |  |  |  |  |  |  |  |  |  |
| V1 Ecto A |  |  |  |  |  |  |  |  |  |  |
| V1 Ecto O |  |  |  |  |  |  |  |  |  |  |
| Mesomere |  |  |  |  |  |  |  |  |  |  |
| Ecto A |  |  |  |  |  |  |  |  |  |  |
| Ecto O |  |  |  |  |  |  |  |  |  |  |
| Stomodeum |  |  |  |  |  |  |  |  |  |  |
| Oral Face |  |  |  |  |  |  |  |  |  |  |
| Ciliated Band |  |  |  |  |  |  |  |  |  |  |
| Apical Plate |  |  |  |  |  |  |  |  |  |  |

Predicted interaction: SPU_024918 (NeuroD) → SPU_017404 (Nr1h6a)

Absolute confidence value: 0.00000956440245841182

*Gene expressions is not available for SPU_024918 (NeuroD) or SPU_017404 (Nr1h6a)

Result: missing complete spatial expression data

|  | **Node1: NeuroD*** | **Node2: Nr1h6a*** | **Both expressed** |
| --- | --- | --- | --- |
| Weak expression |  |  |  |
| Expressed |  |  |  |

|  | **0hr** | **6hr** | **9hr** | **12hr** | **15hr** | **18hr** | **21hr** | **24hr** | **27hr** | **30hr** |
| --- | --- | --- | --- | --- | --- | --- | --- | --- | --- | --- |
| Egg |  |  |  |  |  |  |  |  |  |  |
| Small Micromere |  |  |  |  |  |  |  |  |  |  |
| Skel. Micromere |  |  |  |  |  |  |  |  |  |  |
| Macromere |  |  |  |  |  |  |  |  |  |  |
| V2 |  |  |  |  |  |  |  |  |  |  |
| V2 Meso A |  |  |  |  |  |  |  |  |  |  |
| V2 Meso O |  |  |  |  |  |  |  |  |  |  |
| V2 Endo |  |  |  |  |  |  |  |  |  |  |
| V1 |  |  |  |  |  |  |  |  |  |  |
| V1 Endo A |  |  |  |  |  |  |  |  |  |  |
| V1 Endo O |  |  |  |  |  |  |  |  |  |  |
| V1 Ecto A |  |  |  |  |  |  |  |  |  |  |
| V1 Ecto O |  |  |  |  |  |  |  |  |  |  |
| Mesomere |  |  |  |  |  |  |  |  |  |  |
| Ecto A |  |  |  |  |  |  |  |  |  |  |
| Ecto O |  |  |  |  |  |  |  |  |  |  |
| Stomodeum |  |  |  |  |  |  |  |  |  |  |
| Oral Face |  |  |  |  |  |  |  |  |  |  |
| Ciliated Band |  |  |  |  |  |  |  |  |  |  |
| Apical Plate |  |  |  |  |  |  |  |  |  |  |

Predicted interaction: SPU_027235 (Blimp1a/b) → SPU_009079 (FgfrL5)

Absolute confidence value:

*Gene expression is not available for SPU_009079 (FgfrL5)

Result: missing complete spatial expression data

|  | **Node1: Blimp1** | **Node2: FgfrL5*** | **Both expressed** |
| --- | --- | --- | --- |
| Weak expression |  |  |  |
| Expressed |  |  |  |

|  | **0hr** | **6hr** | **9hr** | **12hr** | **15hr** | **18hr** | **21hr** | **24hr** | **27hr** | **30hr** |
| --- | --- | --- | --- | --- | --- | --- | --- | --- | --- | --- |
| Egg |  |  |  |  |  |  |  |  |  |  |
| Small Micromere |  |  |  |  |  |  |  |  |  |  |
| Skel. Micromere |  |  |  |  |  |  |  |  |  |  |
| Macromere |  |  |  |  |  |  |  |  |  |  |
| V2 |  |  |  |  |  |  |  |  |  |  |
| V2 Meso A |  |  |  |  |  |  |  |  |  |  |
| V2 Meso O |  |  |  |  |  |  |  |  |  |  |
| V2 Endo |  |  |  |  |  |  |  |  |  |  |
| V1 |  |  |  |  |  |  |  |  |  |  |
| V1 Endo A |  |  |  |  |  |  |  |  |  |  |
| V1 Endo O |  |  |  |  |  |  |  |  |  |  |
| V1 Ecto A |  |  |  |  |  |  |  |  |  |  |
| V1 Ecto O |  |  |  |  |  |  |  |  |  |  |
| Mesomere |  |  |  |  |  |  |  |  |  |  |
| Ecto A |  |  |  |  |  |  |  |  |  |  |
| Ecto O |  |  |  |  |  |  |  |  |  |  |
| Stomodeum |  |  |  |  |  |  |  |  |  |  |
| Oral Face |  |  |  |  |  |  |  |  |  |  |
| Ciliated Band |  |  |  |  |  |  |  |  |  |  |
| Apical Plate |  |  |  |  |  |  |  |  |  |  |

Predicted interaction: SPU_017379 (Six1/2) → SPU_001415 (Z200)

Absolute confidence value: 0.0000185743468032925

*Gene expression is not available for SPU_017379 (Six1/2) or SPU_001415 (Z200)

Result: missing complete spatial expression data

|  | **Node1: Six1/2*** | **Node2: Z200*** | **Both expressed** |
| --- | --- | --- | --- |
| Weak expression |  |  |  |
| Expressed |  |  |  |

|  | **0hr** | **6hr** | **9hr** | **12hr** | **15hr** | **18hr** | **21hr** | **24hr** | **27hr** | **30hr** |
| --- | --- | --- | --- | --- | --- | --- | --- | --- | --- | --- |
| Egg |  |  |  |  |  |  |  |  |  |  |
| Small Micromere |  |  |  |  |  |  |  |  |  |  |
| Skel. Micromere |  |  |  |  |  |  |  |  |  |  |
| Macromere |  |  |  |  |  |  |  |  |  |  |
| V2 |  |  |  |  |  |  |  |  |  |  |
| V2 Meso A |  |  |  |  |  |  |  |  |  |  |
| V2 Meso O |  |  |  |  |  |  |  |  |  |  |
| V2 Endo |  |  |  |  |  |  |  |  |  |  |
| V1 |  |  |  |  |  |  |  |  |  |  |
| V1 Endo A |  |  |  |  |  |  |  |  |  |  |
| V1 Endo O |  |  |  |  |  |  |  |  |  |  |
| V1 Ecto A |  |  |  |  |  |  |  |  |  |  |
| V1 Ecto O |  |  |  |  |  |  |  |  |  |  |
| Mesomere |  |  |  |  |  |  |  |  |  |  |
| Ecto A |  |  |  |  |  |  |  |  |  |  |
| Ecto O |  |  |  |  |  |  |  |  |  |  |
| Stomodeum |  |  |  |  |  |  |  |  |  |  |
| Oral Face |  |  |  |  |  |  |  |  |  |  |
| Ciliated Band |  |  |  |  |  |  |  |  |  |  |
| Apical Plate |  |  |  |  |  |  |  |  |  |  |

Predicted interaction: SPU_008117 (Shr2) → SPU_017404 (Nr1h6a)

Absolute confidence value: 0.0000200991066155031

*Gene expression is not available for SPU_017404

Result: missing complete spatial expression data

|  | **Node1: Shr2** | **Node2: Nr1h6a*** | **Both expressed** |
| --- | --- | --- | --- |
| Weak expression |  |  |  |
| Expressed |  |  |  |

|  | **0hr** | **6hr** | **9hr** | **12hr** | **15hr** | **18hr** | **21hr** | **24hr** | **27hr** | **30hr** |
| --- | --- | --- | --- | --- | --- | --- | --- | --- | --- | --- |
| Egg |  |  |  |  |  |  |  |  |  |  |
| Small Micromere |  |  |  |  |  |  |  |  |  |  |
| Skel. Micromere |  |  |  |  |  |  |  |  |  |  |
| Macromere |  |  |  |  |  |  |  |  |  |  |
| V2 |  |  |  |  |  |  |  |  |  |  |
| V2 Meso A |  |  |  |  |  |  |  |  |  |  |
| V2 Meso O |  |  |  |  |  |  |  |  |  |  |
| V2 Endo |  |  |  |  |  |  |  |  |  |  |
| V1 |  |  |  |  |  |  |  |  |  |  |
| V1 Endo A |  |  |  |  |  |  |  |  |  |  |
| V1 Endo O |  |  |  |  |  |  |  |  |  |  |
| V1 Ecto A |  |  |  |  |  |  |  |  |  |  |
| V1 Ecto O |  |  |  |  |  |  |  |  |  |  |
| Mesomere |  |  |  |  |  |  |  |  |  |  |
| Ecto A |  |  |  |  |  |  |  |  |  |  |
| Ecto O |  |  |  |  |  |  |  |  |  |  |
| Stomodeum |  |  |  |  |  |  |  |  |  |  |
| Oral Face |  |  |  |  |  |  |  |  |  |  |
| Ciliated Band |  |  |  |  |  |  |  |  |  |  |
| Apical Plate |  |  |  |  |  |  |  |  |  |  |

Predicted interaction: SPU_025590 (FoxM) → SPU_015199 (Z185.1)

Absolute confidence value: 0.00002023772114

*Gene expression is not available for SPU_025590 (FoxM) or SPU_015199 (Z185.1)

Result: missing complete spatial expression data

|  | **Node1: FoxM*** | **Node2: Z185.1*** | **Both expressed** |
| --- | --- | --- | --- |
| Weak expression |  |  |  |
| Expressed |  |  |  |

|  | **0hr** | **6hr** | **9hr** | **12hr** | **15hr** | **18hr** | **21hr** | **24hr** | **27hr** | **30hr** |
| --- | --- | --- | --- | --- | --- | --- | --- | --- | --- | --- |
| Egg |  |  |  |  |  |  |  |  |  |  |
| Small Micromere |  |  |  |  |  |  |  |  |  |  |
| Skel. Micromere |  |  |  |  |  |  |  |  |  |  |
| Macromere |  |  |  |  |  |  |  |  |  |  |
| V2 |  |  |  |  |  |  |  |  |  |  |
| V2 Meso A |  |  |  |  |  |  |  |  |  |  |
| V2 Meso O |  |  |  |  |  |  |  |  |  |  |
| V2 Endo |  |  |  |  |  |  |  |  |  |  |
| V1 |  |  |  |  |  |  |  |  |  |  |
| V1 Endo A |  |  |  |  |  |  |  |  |  |  |
| V1 Endo O |  |  |  |  |  |  |  |  |  |  |
| V1 Ecto A |  |  |  |  |  |  |  |  |  |  |
| V1 Ecto O |  |  |  |  |  |  |  |  |  |  |
| Mesomere |  |  |  |  |  |  |  |  |  |  |
| Ecto A |  |  |  |  |  |  |  |  |  |  |
| Ecto O |  |  |  |  |  |  |  |  |  |  |
| Stomodeum |  |  |  |  |  |  |  |  |  |  |
| Oral Face |  |  |  |  |  |  |  |  |  |  |
| Ciliated Band |  |  |  |  |  |  |  |  |  |  |
| Apical Plate |  |  |  |  |  |  |  |  |  |  |

Predicted interaction: SPU_006808 (Af9) → SPU_026763 (Scmh1)

Absolute confidence value: 0.0000248120005805176

*Gene expression is not available for SPU_006808 (Af9) or SPU_026763 (Scmh1)

Result: missing complete spatial expression data

|  | **Node1: Af9*** | **Node2: Scmh1*** | **Both expressed** |
| --- | --- | --- | --- |
| Weak expression |  |  |  |
| Expressed |  |  |  |

|  | **0hr** | **6hr** | **9hr** | **12hr** | **15hr** | **18hr** | **21hr** | **24hr** | **27hr** | **30hr** |
| --- | --- | --- | --- | --- | --- | --- | --- | --- | --- | --- |
| Egg |  |  |  |  |  |  |  |  |  |  |
| Small Micromere |  |  |  |  |  |  |  |  |  |  |
| Skel. Micromere |  |  |  |  |  |  |  |  |  |  |
| Macromere |  |  |  |  |  |  |  |  |  |  |
| V2 |  |  |  |  |  |  |  |  |  |  |
| V2 Meso A |  |  |  |  |  |  |  |  |  |  |
| V2 Meso O |  |  |  |  |  |  |  |  |  |  |
| V2 Endo |  |  |  |  |  |  |  |  |  |  |
| V1 |  |  |  |  |  |  |  |  |  |  |
| V1 Endo A |  |  |  |  |  |  |  |  |  |  |
| V1 Endo O |  |  |  |  |  |  |  |  |  |  |
| V1 Ecto A |  |  |  |  |  |  |  |  |  |  |
| V1 Ecto O |  |  |  |  |  |  |  |  |  |  |
| Mesomere |  |  |  |  |  |  |  |  |  |  |
| Ecto A |  |  |  |  |  |  |  |  |  |  |
| Ecto O |  |  |  |  |  |  |  |  |  |  |
| Stomodeum |  |  |  |  |  |  |  |  |  |  |
| Oral Face |  |  |  |  |  |  |  |  |  |  |
| Ciliated Band |  |  |  |  |  |  |  |  |  |  |
| Apical Plate |  |  |  |  |  |  |  |  |  |  |

Predicted interaction: SPU_004287 (Smad4) → SPU_014687 (Z310_1)

Absolute confidence value: 0.0000286932073752354

*Gene expression is not available from 0-30hrs for SPU_004287 (Smad4) or SPU_014687 (Z310_1)

Result: missing complete spatial expression data

|  | **Node1: Smad4** | **Node2: Z310_1*** | **Both expressed** |
| --- | --- | --- | --- |
| Weak expression |  |  |  |
| Expressed |  |  |  |

|  | **0hr** | **6hr** | **9hr** | **12hr** | **15hr** | **18hr** | **21hr** | **24hr** | **27hr** | **30hr** |
| --- | --- | --- | --- | --- | --- | --- | --- | --- | --- | --- |
| Egg |  |  |  |  |  |  |  |  |  |  |
| Small Micromere |  |  |  |  |  |  |  |  |  |  |
| Skel. Micromere |  |  |  |  |  |  |  |  |  |  |
| Macromere |  |  |  |  |  |  |  |  |  |  |
| V2 |  |  |  |  |  |  |  |  |  |  |
| V2 Meso A |  |  |  |  |  |  |  |  |  |  |
| V2 Meso O |  |  |  |  |  |  |  |  |  |  |
| V2 Endo |  |  |  |  |  |  |  |  |  |  |
| V1 |  |  |  |  |  |  |  |  |  |  |
| V1 Endo A |  |  |  |  |  |  |  |  |  |  |
| V1 Endo O |  |  |  |  |  |  |  |  |  |  |
| V1 Ecto A |  |  |  |  |  |  |  |  |  |  |
| V1 Ecto O |  |  |  |  |  |  |  |  |  |  |
| Mesomere |  |  |  |  |  |  |  |  |  |  |
| Ecto A |  |  |  |  |  |  |  |  |  |  |
| Ecto O |  |  |  |  |  |  |  |  |  |  |
| Stomodeum |  |  |  |  |  |  |  |  |  |  |
| Oral Face |  |  |  |  |  |  |  |  |  |  |
| Ciliated Band |  |  |  |  |  |  |  |  |  |  |
| Apical Plate |  |  |  |  |  |  |  |  |  |  |

Predicted interaction: SPU_006312 (Dp1) → SPU_002554 (Phf20L)

Absolute confidence value: 0.0000291090509603838

*Gene expression is not available for SPU_006312 (Dp1) or SPU_002554 (Phf20L)

Result: missing complete spatial expression data

|  | **Node1: Dp1*** | **Node2: Phf20L*** | **Both expressed** |
| --- | --- | --- | --- |
| Weak expression |  |  |  |
| Expressed |  |  |  |

|  | **0hr** | **6hr** | **9hr** | **12hr** | **15hr** | **18hr** | **21hr** | **24hr** | **27hr** | **30hr** |
| --- | --- | --- | --- | --- | --- | --- | --- | --- | --- | --- |
| Egg |  |  |  |  |  |  |  |  |  |  |
| Small Micromere |  |  |  |  |  |  |  |  |  |  |
| Skel. Micromere |  |  |  |  |  |  |  |  |  |  |
| Macromere |  |  |  |  |  |  |  |  |  |  |
| V2 |  |  |  |  |  |  |  |  |  |  |
| V2 Meso A |  |  |  |  |  |  |  |  |  |  |
| V2 Meso O |  |  |  |  |  |  |  |  |  |  |
| V2 Endo |  |  |  |  |  |  |  |  |  |  |
| V1 |  |  |  |  |  |  |  |  |  |  |
| V1 Endo A |  |  |  |  |  |  |  |  |  |  |
| V1 Endo O |  |  |  |  |  |  |  |  |  |  |
| V1 Ecto A |  |  |  |  |  |  |  |  |  |  |
| V1 Ecto O |  |  |  |  |  |  |  |  |  |  |
| Mesomere |  |  |  |  |  |  |  |  |  |  |
| Ecto A |  |  |  |  |  |  |  |  |  |  |
| Ecto O |  |  |  |  |  |  |  |  |  |  |
| Stomodeum |  |  |  |  |  |  |  |  |  |  |
| Oral Face |  |  |  |  |  |  |  |  |  |  |
| Ciliated Band |  |  |  |  |  |  |  |  |  |  |
| Apical Plate |  |  |  |  |  |  |  |  |  |  |

Predicted interaction: SPU_004287 (Smad4) → SPU_013810 (Ptpn6/1)

Absolute confidence value: 0.00002938628002

*Gene expression is not available from 0-30hrs for SPU_004287 (Smad4) or SPU_013810 (Ptpn6/1)

Result: missing complete spatial expression data

|  | **Node1: Smad4*** | **Node2: Ptpn6/1*** | **Both expressed** |
| --- | --- | --- | --- |
| Weak expression |  |  |  |
| Expressed |  |  |  |

|  | **0hr** | **6hr** | **9hr** | **12hr** | **15hr** | **18hr** | **21hr** | **24hr** | **27hr** | **30hr** |
| --- | --- | --- | --- | --- | --- | --- | --- | --- | --- | --- |
| Egg |  |  |  |  |  |  |  |  |  |  |
| Small Micromere |  |  |  |  |  |  |  |  |  |  |
| Skel. Micromere |  |  |  |  |  |  |  |  |  |  |
| Macromere |  |  |  |  |  |  |  |  |  |  |
| V2 |  |  |  |  |  |  |  |  |  |  |
| V2 Meso A |  |  |  |  |  |  |  |  |  |  |
| V2 Meso O |  |  |  |  |  |  |  |  |  |  |
| V2 Endo |  |  |  |  |  |  |  |  |  |  |
| V1 |  |  |  |  |  |  |  |  |  |  |
| V1 Endo A |  |  |  |  |  |  |  |  |  |  |
| V1 Endo O |  |  |  |  |  |  |  |  |  |  |
| V1 Ecto A |  |  |  |  |  |  |  |  |  |  |
| V1 Ecto O |  |  |  |  |  |  |  |  |  |  |
| Mesomere |  |  |  |  |  |  |  |  |  |  |
| Ecto A |  |  |  |  |  |  |  |  |  |  |
| Ecto O |  |  |  |  |  |  |  |  |  |  |
| Stomodeum |  |  |  |  |  |  |  |  |  |  |
| Oral Face |  |  |  |  |  |  |  |  |  |  |
| Ciliated Band |  |  |  |  |  |  |  |  |  |  |
| Apical Plate |  |  |  |  |  |  |  |  |  |  |

Predicted interaction: SPU_007644 (FoxJ2) → SPU_027708 (Z491)

Absolute confidence value: 0.0000300793526590632

*Gene expression is not available for SPU_027708 (Z491)

Result: missing complete spatial expression data

|  | **Node1: FoxJ2** | **Node2: Z491*** | **Both expressed** |
| --- | --- | --- | --- |
| Weak expression |  |  |  |
| Expressed |  |  |  |

|  | **0hr** | **6hr** | **9hr** | **12hr** | **15hr** | **18hr** | **21hr** | **24hr** | **27hr** | **30hr** |
| --- | --- | --- | --- | --- | --- | --- | --- | --- | --- | --- |
| Egg |  |  |  |  |  |  |  |  |  |  |
| Small Micromere |  |  |  |  |  |  |  |  |  |  |
| Skel. Micromere |  |  |  |  |  |  |  |  |  |  |
| Macromere |  |  |  |  |  |  |  |  |  |  |
| V2 |  |  |  |  |  |  |  |  |  |  |
| V2 Meso A |  |  |  |  |  |  |  |  |  |  |
| V2 Meso O |  |  |  |  |  |  |  |  |  |  |
| V2 Endo |  |  |  |  |  |  |  |  |  |  |
| V1 |  |  |  |  |  |  |  |  |  |  |
| V1 Endo A |  |  |  |  |  |  |  |  |  |  |
| V1 Endo O |  |  |  |  |  |  |  |  |  |  |
| V1 Ecto A |  |  |  |  |  |  |  |  |  |  |
| V1 Ecto O |  |  |  |  |  |  |  |  |  |  |
| Mesomere |  |  |  |  |  |  |  |  |  |  |
| Ecto A |  |  |  |  |  |  |  |  |  |  |
| Ecto O |  |  |  |  |  |  |  |  |  |  |
| Stomodeum |  |  |  |  |  |  |  |  |  |  |
| Oral Face |  |  |  |  |  |  |  |  |  |  |
| Ciliated Band |  |  |  |  |  |  |  |  |  |  |
| Apical Plate |  |  |  |  |  |  |  |  |  |  |

Predicted interaction: SPU_004414 (Hlf) → SPU_012111 (Z53)

Absolute confidence value: 0.0000306338107725944

*Gene expression is not available for SPU_012111 (Z53)

Result: missing complete spatial expression data

|  | **Node1: Hlf** | **Node2: Z53*** | **Both expressed** |
| --- | --- | --- | --- |
| Weak expression |  |  |  |
| Expressed |  |  |  |

|  | **0hr** | **6hr** | **9hr** | **12hr** | **15hr** | **18hr** | **21hr** | **24hr** | **27hr** | **30hr** |
| --- | --- | --- | --- | --- | --- | --- | --- | --- | --- | --- |
| Egg |  |  |  |  |  |  |  |  |  |  |
| Small Micromere |  |  |  |  |  |  |  |  |  |  |
| Skel. Micromere |  |  |  |  |  |  |  |  |  |  |
| Macromere |  |  |  |  |  |  |  |  |  |  |
| V2 |  |  |  |  |  |  |  |  |  |  |
| V2 Meso A |  |  |  |  |  |  |  |  |  |  |
| V2 Meso O |  |  |  |  |  |  |  |  |  |  |
| V2 Endo |  |  |  |  |  |  |  |  |  |  |
| V1 |  |  |  |  |  |  |  |  |  |  |
| V1 Endo A |  |  |  |  |  |  |  |  |  |  |
| V1 Endo O |  |  |  |  |  |  |  |  |  |  |
| V1 Ecto A |  |  |  |  |  |  |  |  |  |  |
| V1 Ecto O |  |  |  |  |  |  |  |  |  |  |
| Mesomere |  |  |  |  |  |  |  |  |  |  |
| Ecto A |  |  |  |  |  |  |  |  |  |  |
| Ecto O |  |  |  |  |  |  |  |  |  |  |
| Stomodeum |  |  |  |  |  |  |  |  |  |  |
| Oral Face |  |  |  |  |  |  |  |  |  |  |
| Ciliated Band |  |  |  |  |  |  |  |  |  |  |
| Apical Plate |  |  |  |  |  |  |  |  |  |  |

Predicted interaction: SPU_012008 (MyoR2) → SPU_014461 (Pitx1)

Absolute confidence value: 0.0000325744141699533

*Gene expression is not available from 0-30 hrs for SPU_012008 (MyoR2) or SPU_014461 (Pitx1)

Result: missing complete spatial expression data

|  | **Node1: MyoR2*** | **Node2: Pitx1*** | **Both expressed** |
| --- | --- | --- | --- |
| Weak expression |  |  |  |
| Expressed |  |  |  |

|  | **0hr** | **6hr** | **9hr** | **12hr** | **15hr** | **18hr** | **21hr** | **24hr** | **27hr** | **30hr** |
| --- | --- | --- | --- | --- | --- | --- | --- | --- | --- | --- |
| Egg |  |  |  |  |  |  |  |  |  |  |
| Small Micromere |  |  |  |  |  |  |  |  |  |  |
| Skel. Micromere |  |  |  |  |  |  |  |  |  |  |
| Macromere |  |  |  |  |  |  |  |  |  |  |
| V2 |  |  |  |  |  |  |  |  |  |  |
| V2 Meso A |  |  |  |  |  |  |  |  |  |  |
| V2 Meso O |  |  |  |  |  |  |  |  |  |  |
| V2 Endo |  |  |  |  |  |  |  |  |  |  |
| V1 |  |  |  |  |  |  |  |  |  |  |
| V1 Endo A |  |  |  |  |  |  |  |  |  |  |
| V1 Endo O |  |  |  |  |  |  |  |  |  |  |
| V1 Ecto A |  |  |  |  |  |  |  |  |  |  |
| V1 Ecto O |  |  |  |  |  |  |  |  |  |  |
| Mesomere |  |  |  |  |  |  |  |  |  |  |
| Ecto A |  |  |  |  |  |  |  |  |  |  |
| Ecto O |  |  |  |  |  |  |  |  |  |  |
| Stomodeum |  |  |  |  |  |  |  |  |  |  |
| Oral Face |  |  |  |  |  |  |  |  |  |  |
| Ciliated Band |  |  |  |  |  |  |  |  |  |  |
| Apical Plate |  |  |  |  |  |  |  |  |  |  |

Predicted interaction: SPU_002320 (FoxN1/4) → SPU_021666 (Dok)

Absolute confidence value: 0.0000329902577551016

*Gene expression is not available for SPU_002320 (FoxN1/4) → SPU_021666 (Dok)

Result: missing complete spatial expression data

|  | **Node1: FoxN1/4*** | **Node2: Dok*** | **Both expressed** |
| --- | --- | --- | --- |
| Weak expression |  |  |  |
| Expressed |  |  |  |

|  | **0hr** | **6hr** | **9hr** | **12hr** | **15hr** | **18hr** | **21hr** | **24hr** | **27hr** | **30hr** |
| --- | --- | --- | --- | --- | --- | --- | --- | --- | --- | --- |
| Egg |  |  |  |  |  |  |  |  |  |  |
| Small Micromere |  |  |  |  |  |  |  |  |  |  |
| Skel. Micromere |  |  |  |  |  |  |  |  |  |  |
| Macromere |  |  |  |  |  |  |  |  |  |  |
| V2 |  |  |  |  |  |  |  |  |  |  |
| V2 Meso A |  |  |  |  |  |  |  |  |  |  |
| V2 Meso O |  |  |  |  |  |  |  |  |  |  |
| V2 Endo |  |  |  |  |  |  |  |  |  |  |
| V1 |  |  |  |  |  |  |  |  |  |  |
| V1 Endo A |  |  |  |  |  |  |  |  |  |  |
| V1 Endo O |  |  |  |  |  |  |  |  |  |  |
| V1 Ecto A |  |  |  |  |  |  |  |  |  |  |
| V1 Ecto O |  |  |  |  |  |  |  |  |  |  |
| Mesomere |  |  |  |  |  |  |  |  |  |  |
| Ecto A |  |  |  |  |  |  |  |  |  |  |
| Ecto O |  |  |  |  |  |  |  |  |  |  |
| Stomodeum |  |  |  |  |  |  |  |  |  |  |
| Oral Face |  |  |  |  |  |  |  |  |  |  |
| Ciliated Band |  |  |  |  |  |  |  |  |  |  |
| Apical Plate |  |  |  |  |  |  |  |  |  |  |

Predicted interaction: SPU_003166 (Myc) → SPU_020330 (Grem)

Absolute confidence value: 0.0000331288722834844

*Gene expression is not available for SPU_020330 (Grem)

Result: missing complete spatial expression data

|  | **Node1: Myc** | **Node2: Grem*** | **Both expressed** |
| --- | --- | --- | --- |
| Weak expression |  |  |  |
| Expressed |  |  |  |

|  | **0hr** | **6hr** | **9hr** | **12hr** | **15hr** | **18hr** | **21hr** | **24hr** | **27hr** | **30hr** |
| --- | --- | --- | --- | --- | --- | --- | --- | --- | --- | --- |
| Egg |  |  |  |  |  |  |  |  |  |  |
| Small Micromere |  |  |  |  |  |  |  |  |  |  |
| Skel. Micromere |  |  |  |  |  |  |  |  |  |  |
| Macromere |  |  |  |  |  |  |  |  |  |  |
| V2 |  |  |  |  |  |  |  |  |  |  |
| V2 Meso A |  |  |  |  |  |  |  |  |  |  |
| V2 Meso O |  |  |  |  |  |  |  |  |  |  |
| V2 Endo |  |  |  |  |  |  |  |  |  |  |
| V1 |  |  |  |  |  |  |  |  |  |  |
| V1 Endo A |  |  |  |  |  |  |  |  |  |  |
| V1 Endo O |  |  |  |  |  |  |  |  |  |  |
| V1 Ecto A |  |  |  |  |  |  |  |  |  |  |
| V1 Ecto O |  |  |  |  |  |  |  |  |  |  |
| Mesomere |  |  |  |  |  |  |  |  |  |  |
| Ecto A |  |  |  |  |  |  |  |  |  |  |
| Ecto O |  |  |  |  |  |  |  |  |  |  |
| Stomodeum |  |  |  |  |  |  |  |  |  |  |
| Oral Face |  |  |  |  |  |  |  |  |  |  |
| Ciliated Band |  |  |  |  |  |  |  |  |  |  |
| Apical Plate |  |  |  |  |  |  |  |  |  |  |

Predicted interaction: SPU_019332 (Ppar1) → SPU_013305 (Grf)

Absolute confidence value: 0.0000332674868118672

*Gene expression is not available for SPU_019332 (Ppar1) or SPU_013305 (Grf)

Result: missing complete spatial expression data

|  | **Node1: Ppar1*** | **Node2: Grf*** | **Both expressed** |
| --- | --- | --- | --- |
| Weak expression |  |  |  |
| Expressed |  |  |  |

|  | **0hr** | **6hr** | **9hr** | **12hr** | **15hr** | **18hr** | **21hr** | **24hr** | **27hr** | **30hr** |
| --- | --- | --- | --- | --- | --- | --- | --- | --- | --- | --- |
| Egg |  |  |  |  |  |  |  |  |  |  |
| Small Micromere |  |  |  |  |  |  |  |  |  |  |
| Skel. Micromere |  |  |  |  |  |  |  |  |  |  |
| Macromere |  |  |  |  |  |  |  |  |  |  |
| V2 |  |  |  |  |  |  |  |  |  |  |
| V2 Meso A |  |  |  |  |  |  |  |  |  |  |
| V2 Meso O |  |  |  |  |  |  |  |  |  |  |
| V2 Endo |  |  |  |  |  |  |  |  |  |  |
| V1 |  |  |  |  |  |  |  |  |  |  |
| V1 Endo A |  |  |  |  |  |  |  |  |  |  |
| V1 Endo O |  |  |  |  |  |  |  |  |  |  |
| V1 Ecto A |  |  |  |  |  |  |  |  |  |  |
| V1 Ecto O |  |  |  |  |  |  |  |  |  |  |
| Mesomere |  |  |  |  |  |  |  |  |  |  |
| Ecto A |  |  |  |  |  |  |  |  |  |  |
| Ecto O |  |  |  |  |  |  |  |  |  |  |
| Stomodeum |  |  |  |  |  |  |  |  |  |  |
| Oral Face |  |  |  |  |  |  |  |  |  |  |
| Ciliated Band |  |  |  |  |  |  |  |  |  |  |
| Apical Plate |  |  |  |  |  |  |  |  |  |  |

Predicted interaction: SPU_004148 (Krl) → SPU_000806 (Cad96cal)

Absolute confidence value: 0.0000335447158686327

*Gene expression is not available for SPU_000806 (Cad96cal)

Result: missing complete spatial expression data

|  | **Node1: Krl** | **Node2: Cad96cal*** | **Both expressed** |
| --- | --- | --- | --- |
| Weak expression |  |  |  |
| Expressed |  |  |  |

|  | **0hr** | **6hr** | **9hr** | **12hr** | **15hr** | **18hr** | **21hr** | **24hr** | **27hr** | **30hr** |
| --- | --- | --- | --- | --- | --- | --- | --- | --- | --- | --- |
| Egg |  |  |  |  |  |  |  |  |  |  |
| Small Micromere |  |  |  |  |  |  |  |  |  |  |
| Skel. Micromere |  |  |  |  |  |  |  |  |  |  |
| Macromere |  |  |  |  |  |  |  |  |  |  |
| V2 |  |  |  |  |  |  |  |  |  |  |
| V2 Meso A |  |  |  |  |  |  |  |  |  |  |
| V2 Meso O |  |  |  |  |  |  |  |  |  |  |
| V2 Endo |  |  |  |  |  |  |  |  |  |  |
| V1 |  |  |  |  |  |  |  |  |  |  |
| V1 Endo A |  |  |  |  |  |  |  |  |  |  |
| V1 Endo O |  |  |  |  |  |  |  |  |  |  |
| V1 Ecto A |  |  |  |  |  |  |  |  |  |  |
| V1 Ecto O |  |  |  |  |  |  |  |  |  |  |
| Mesomere |  |  |  |  |  |  |  |  |  |  |
| Ecto A |  |  |  |  |  |  |  |  |  |  |
| Ecto O |  |  |  |  |  |  |  |  |  |  |
| Stomodeum |  |  |  |  |  |  |  |  |  |  |
| Oral Face |  |  |  |  |  |  |  |  |  |  |
| Ciliated Band |  |  |  |  |  |  |  |  |  |  |
| Apical Plate |  |  |  |  |  |  |  |  |  |  |

Predicted interaction: SPU_012772 (Klf7) → SPU_000483 (Z353)

Absolute confidence value: 0.0000356239337943744

*Gene expression is not available for SPU_012772 (Klf7) or SPU_000483 (Z353)

Result: missing complete spatial expression data

|  | **Node1: Klf7*** | **Node2: Z353*** | **Both expressed** |
| --- | --- | --- | --- |
| Weak expression |  |  |  |
| Expressed |  |  |  |

|  | **0hr** | **6hr** | **9hr** | **12hr** | **15hr** | **18hr** | **21hr** | **24hr** | **27hr** | **30hr** |
| --- | --- | --- | --- | --- | --- | --- | --- | --- | --- | --- |
| Egg |  |  |  |  |  |  |  |  |  |  |
| Small Micromere |  |  |  |  |  |  |  |  |  |  |
| Skel. Micromere |  |  |  |  |  |  |  |  |  |  |
| Macromere |  |  |  |  |  |  |  |  |  |  |
| V2 |  |  |  |  |  |  |  |  |  |  |
| V2 Meso A |  |  |  |  |  |  |  |  |  |  |
| V2 Meso O |  |  |  |  |  |  |  |  |  |  |
| V2 Endo |  |  |  |  |  |  |  |  |  |  |
| V1 |  |  |  |  |  |  |  |  |  |  |
| V1 Endo A |  |  |  |  |  |  |  |  |  |  |
| V1 Endo O |  |  |  |  |  |  |  |  |  |  |
| V1 Ecto A |  |  |  |  |  |  |  |  |  |  |
| V1 Ecto O |  |  |  |  |  |  |  |  |  |  |
| Mesomere |  |  |  |  |  |  |  |  |  |  |
| Ecto A |  |  |  |  |  |  |  |  |  |  |
| Ecto O |  |  |  |  |  |  |  |  |  |  |
| Stomodeum |  |  |  |  |  |  |  |  |  |  |
| Oral Face |  |  |  |  |  |  |  |  |  |  |
| Ciliated Band |  |  |  |  |  |  |  |  |  |  |
| Apical Plate |  |  |  |  |  |  |  |  |  |  |

Predicted interaction: SPU_026905 (Atf2) → SPU_004148 (Krl)

Absolute confidence value: 0.0000393665260607095

*Gene expression is not available for SPU_026905 (Atf2)

Result: missing complete spatial expression data

|  | **Node1: Atf2*** | **Node2: Krl** | **Both expressed** |
| --- | --- | --- | --- |
| Weak expression |  |  |  |
| Expressed |  |  |  |

|  | **0hr** | **6hr** | **9hr** | **12hr** | **15hr** | **18hr** | **21hr** | **24hr** | **27hr** | **30hr** |
| --- | --- | --- | --- | --- | --- | --- | --- | --- | --- | --- |
| Egg |  |  |  |  |  |  |  |  |  |  |
| Small Micromere |  |  |  |  |  |  |  |  |  |  |
| Skel. Micromere |  |  |  |  |  |  |  |  |  |  |
| Macromere |  |  |  |  |  |  |  |  |  |  |
| V2 |  |  |  |  |  |  |  |  |  |  |
| V2 Meso A |  |  |  |  |  |  |  |  |  |  |
| V2 Meso O |  |  |  |  |  |  |  |  |  |  |
| V2 Endo |  |  |  |  |  |  |  |  |  |  |
| V1 |  |  |  |  |  |  |  |  |  |  |
| V1 Endo A |  |  |  |  |  |  |  |  |  |  |
| V1 Endo O |  |  |  |  |  |  |  |  |  |  |
| V1 Ecto A |  |  |  |  |  |  |  |  |  |  |
| V1 Ecto O |  |  |  |  |  |  |  |  |  |  |
| Mesomere |  |  |  |  |  |  |  |  |  |  |
| Ecto A |  |  |  |  |  |  |  |  |  |  |
| Ecto O |  |  |  |  |  |  |  |  |  |  |
| Stomodeum |  |  |  |  |  |  |  |  |  |  |
| Oral Face |  |  |  |  |  |  |  |  |  |  |
| Ciliated Band |  |  |  |  |  |  |  |  |  |  |
| Apical Plate |  |  |  |  |  |  |  |  |  |  |

Predicted interaction: SPU_005358 (Crem) → SPU_013140 (Ron)

Absolute confidence value: 0.0000422774311567479

*Gene expression is not available for SPU_005358 (Crem) or SPU_013140 (Ron)

Result: missing complete spatial expression data

|  | **Node1: Crem*** | **Node2: Ron*** | **Both expressed** |
| --- | --- | --- | --- |
| Weak expression |  |  |  |
| Expressed |  |  |  |

|  | **0hr** | **6hr** | **9hr** | **12hr** | **15hr** | **18hr** | **21hr** | **24hr** | **27hr** | **30hr** |
| --- | --- | --- | --- | --- | --- | --- | --- | --- | --- | --- |
| Egg |  |  |  |  |  |  |  |  |  |  |
| Small Micromere |  |  |  |  |  |  |  |  |  |  |
| Skel. Micromere |  |  |  |  |  |  |  |  |  |  |
| Macromere |  |  |  |  |  |  |  |  |  |  |
| V2 |  |  |  |  |  |  |  |  |  |  |
| V2 Meso A |  |  |  |  |  |  |  |  |  |  |
| V2 Meso O |  |  |  |  |  |  |  |  |  |  |
| V2 Endo |  |  |  |  |  |  |  |  |  |  |
| V1 |  |  |  |  |  |  |  |  |  |  |
| V1 Endo A |  |  |  |  |  |  |  |  |  |  |
| V1 Endo O |  |  |  |  |  |  |  |  |  |  |
| V1 Ecto A |  |  |  |  |  |  |  |  |  |  |
| V1 Ecto O |  |  |  |  |  |  |  |  |  |  |
| Mesomere |  |  |  |  |  |  |  |  |  |  |
| Ecto A |  |  |  |  |  |  |  |  |  |  |
| Ecto O |  |  |  |  |  |  |  |  |  |  |
| Stomodeum |  |  |  |  |  |  |  |  |  |  |
| Oral Face |  |  |  |  |  |  |  |  |  |  |
| Ciliated Band |  |  |  |  |  |  |  |  |  |  |
| Apical Plate |  |  |  |  |  |  |  |  |  |  |

Predicted interaction: SPU_017379 (Six1/2) → SPU_015829 (Pmar1d)

Absolute confidence value: 0.0000439408054973413

Result: Non-overlapping

|  | **Node1: Six1/2** | **Node2: Pmar1d** | **Both expressed** |
| --- | --- | --- | --- |
| Weak expression |  |  |  |
| Expressed |  |  |  |

|  | **0hr** | **6hr** | **9hr** | **12hr** | **15hr** | **18hr** | **21hr** | **24hr** | **27hr** | **30hr** |
| --- | --- | --- | --- | --- | --- | --- | --- | --- | --- | --- |
| Egg |  |  |  |  |  |  |  |  |  |  |
| Small Micromere |  |  |  |  |  |  |  |  |  |  |
| Skel. Micromere |  |  |  |  |  |  |  |  |  |  |
| Macromere |  |  |  |  |  |  |  |  |  |  |
| V2 |  |  |  |  |  |  |  |  |  |  |
| V2 Meso A |  |  |  |  |  |  |  |  |  |  |
| V2 Meso O |  |  |  |  |  |  |  |  |  |  |
| V2 Endo |  |  |  |  |  |  |  |  |  |  |
| V1 |  |  |  |  |  |  |  |  |  |  |
| V1 Endo A |  |  |  |  |  |  |  |  |  |  |
| V1 Endo O |  |  |  |  |  |  |  |  |  |  |
| V1 Ecto A |  |  |  |  |  |  |  |  |  |  |
| V1 Ecto O |  |  |  |  |  |  |  |  |  |  |
| Mesomere |  |  |  |  |  |  |  |  |  |  |
| Ecto A |  |  |  |  |  |  |  |  |  |  |
| Ecto O |  |  |  |  |  |  |  |  |  |  |
| Stomodeum |  |  |  |  |  |  |  |  |  |  |
| Oral Face |  |  |  |  |  |  |  |  |  |  |
| Ciliated Band |  |  |  |  |  |  |  |  |  |  |
| Apical Plate |  |  |  |  |  |  |  |  |  |  |

Predicted interaction: SPU_017407 (Clock) → SPU_020646 (Ror)

Absolute confidence value: 0.0000443566490824896

*Gene expression is not available for SPU_017407 (Clock) or SPU_020646 (Ror)

Result: missing complete spatial expression data

|  | **Node1: Clock*** | **Node2: Ror*** | **Both expressed** |
| --- | --- | --- | --- |
| Weak expression |  |  |  |
| Expressed |  |  |  |

|  | **0hr** | **6hr** | **9hr** | **12hr** | **15hr** | **18hr** | **21hr** | **24hr** | **27hr** | **30hr** |
| --- | --- | --- | --- | --- | --- | --- | --- | --- | --- | --- |
| Egg |  |  |  |  |  |  |  |  |  |  |
| Small Micromere |  |  |  |  |  |  |  |  |  |  |
| Skel. Micromere |  |  |  |  |  |  |  |  |  |  |
| Macromere |  |  |  |  |  |  |  |  |  |  |
| V2 |  |  |  |  |  |  |  |  |  |  |
| V2 Meso A |  |  |  |  |  |  |  |  |  |  |
| V2 Meso O |  |  |  |  |  |  |  |  |  |  |
| V2 Endo |  |  |  |  |  |  |  |  |  |  |
| V1 |  |  |  |  |  |  |  |  |  |  |
| V1 Endo A |  |  |  |  |  |  |  |  |  |  |
| V1 Endo O |  |  |  |  |  |  |  |  |  |  |
| V1 Ecto A |  |  |  |  |  |  |  |  |  |  |
| V1 Ecto O |  |  |  |  |  |  |  |  |  |  |
| Mesomere |  |  |  |  |  |  |  |  |  |  |
| Ecto A |  |  |  |  |  |  |  |  |  |  |
| Ecto O |  |  |  |  |  |  |  |  |  |  |
| Stomodeum |  |  |  |  |  |  |  |  |  |  |
| Oral Face |  |  |  |  |  |  |  |  |  |  |
| Ciliated Band |  |  |  |  |  |  |  |  |  |  |
| Apical Plate |  |  |  |  |  |  |  |  |  |  |

Predicted interaction: SPU_012772 (Klf7) → SPU_022678 (RoraL)

Absolute confidence value: 0.000046020023423083

*Gene expressions is not available for Predicted interaction: SPU_012772 (Klf7) or SPU_022678 (RoraL)

Result: missing complete spatial expression data

|  | **Node1: Klf7*** | **Node2: RoraL*** | **Both expressed** |
| --- | --- | --- | --- |
| Weak expression |  |  |  |
| Expressed |  |  |  |

|  | **0hr** | **6hr** | **9hr** | **12hr** | **15hr** | **18hr** | **21hr** | **24hr** | **27hr** | **30hr** |
| --- | --- | --- | --- | --- | --- | --- | --- | --- | --- | --- |
| Egg |  |  |  |  |  |  |  |  |  |  |
| Small Micromere |  |  |  |  |  |  |  |  |  |  |
| Skel. Micromere |  |  |  |  |  |  |  |  |  |  |
| Macromere |  |  |  |  |  |  |  |  |  |  |
| V2 |  |  |  |  |  |  |  |  |  |  |
| V2 Meso A |  |  |  |  |  |  |  |  |  |  |
| V2 Meso O |  |  |  |  |  |  |  |  |  |  |
| V2 Endo |  |  |  |  |  |  |  |  |  |  |
| V1 |  |  |  |  |  |  |  |  |  |  |
| V1 Endo A |  |  |  |  |  |  |  |  |  |  |
| V1 Endo O |  |  |  |  |  |  |  |  |  |  |
| V1 Ecto A |  |  |  |  |  |  |  |  |  |  |
| V1 Ecto O |  |  |  |  |  |  |  |  |  |  |
| Mesomere |  |  |  |  |  |  |  |  |  |  |
| Ecto A |  |  |  |  |  |  |  |  |  |  |
| Ecto O |  |  |  |  |  |  |  |  |  |  |
| Stomodeum |  |  |  |  |  |  |  |  |  |  |
| Oral Face |  |  |  |  |  |  |  |  |  |  |
| Ciliated Band |  |  |  |  |  |  |  |  |  |  |
| Apical Plate |  |  |  |  |  |  |  |  |  |  |

Predicted interaction: SPU_018126 (Tgif) → SPU_003378 (Plag1L)

Absolute confidence value: 0.0000461586379514657

*Gene expression is not available for SPU_003378 (Plag1L)

Result: missing complete spatial expression data

|  | **Node1: Tgif** | **Node2: Plag1L*** | **Both expressed** |
| --- | --- | --- | --- |
| Weak expression |  |  |  |
| Expressed |  |  |  |

|  | **0hr** | **6hr** | **9hr** | **12hr** | **15hr** | **18hr** | **21hr** | **24hr** | **27hr** | **30hr** |
| --- | --- | --- | --- | --- | --- | --- | --- | --- | --- | --- |
| Egg |  |  |  |  |  |  |  |  |  |  |
| Small Micromere |  |  |  |  |  |  |  |  |  |  |
| Skel. Micromere |  |  |  |  |  |  |  |  |  |  |
| Macromere |  |  |  |  |  |  |  |  |  |  |
| V2 |  |  |  |  |  |  |  |  |  |  |
| V2 Meso A |  |  |  |  |  |  |  |  |  |  |
| V2 Meso O |  |  |  |  |  |  |  |  |  |  |
| V2 Endo |  |  |  |  |  |  |  |  |  |  |
| V1 |  |  |  |  |  |  |  |  |  |  |
| V1 Endo A |  |  |  |  |  |  |  |  |  |  |
| V1 Endo O |  |  |  |  |  |  |  |  |  |  |
| V1 Ecto A |  |  |  |  |  |  |  |  |  |  |
| V1 Ecto O |  |  |  |  |  |  |  |  |  |  |
| Mesomere |  |  |  |  |  |  |  |  |  |  |
| Ecto A |  |  |  |  |  |  |  |  |  |  |
| Ecto O |  |  |  |  |  |  |  |  |  |  |
| Stomodeum |  |  |  |  |  |  |  |  |  |  |
| Oral Face |  |  |  |  |  |  |  |  |  |  |
| Ciliated Band |  |  |  |  |  |  |  |  |  |  |
| Apical Plate |  |  |  |  |  |  |  |  |  |  |

Predicted interaction: SPU_022846 (FoxABL) → SPU_022892 (Z61)

Absolute confidence value: 0.000049762615689418

*Gene expression is not available for SPU_022846 (FoxABL) or SPU_022892 (Z61)

Result: missing complete spatial expression data

|  | **Node1: FoxABL*** | **Node2: Z61*** | **Both expressed** |
| --- | --- | --- | --- |
| Weak expression |  |  |  |
| Expressed |  |  |  |

|  | **0hr** | **6hr** | **9hr** | **12hr** | **15hr** | **18hr** | **21hr** | **24hr** | **27hr** | **30hr** |
| --- | --- | --- | --- | --- | --- | --- | --- | --- | --- | --- |
| Egg |  |  |  |  |  |  |  |  |  |  |
| Small Micromere |  |  |  |  |  |  |  |  |  |  |
| Skel. Micromere |  |  |  |  |  |  |  |  |  |  |
| Macromere |  |  |  |  |  |  |  |  |  |  |
| V2 |  |  |  |  |  |  |  |  |  |  |
| V2 Meso A |  |  |  |  |  |  |  |  |  |  |
| V2 Meso O |  |  |  |  |  |  |  |  |  |  |
| V2 Endo |  |  |  |  |  |  |  |  |  |  |
| V1 |  |  |  |  |  |  |  |  |  |  |
| V1 Endo A |  |  |  |  |  |  |  |  |  |  |
| V1 Endo O |  |  |  |  |  |  |  |  |  |  |
| V1 Ecto A |  |  |  |  |  |  |  |  |  |  |
| V1 Ecto O |  |  |  |  |  |  |  |  |  |  |
| Mesomere |  |  |  |  |  |  |  |  |  |  |
| Ecto A |  |  |  |  |  |  |  |  |  |  |
| Ecto O |  |  |  |  |  |  |  |  |  |  |
| Stomodeum |  |  |  |  |  |  |  |  |  |  |
| Oral Face |  |  |  |  |  |  |  |  |  |  |
| Ciliated Band |  |  |  |  |  |  |  |  |  |  |
| Apical Plate |  |  |  |  |  |  |  |  |  |  |

Predicted interaction: SPU_008528 (Ets4) → SPU_022841 (Z142)

Absolute confidence value: 0.0000499012302178008

*Gene expression is not available for SPU_022841 (Z142)

Result: missing complete spatial expression data

|  | **Node1: Ets4** | **Node2: Z142*** | **Both expressed** |
| --- | --- | --- | --- |
| Weak expression |  |  |  |
| Expressed |  |  |  |

|  | **0hr** | **6hr** | **9hr** | **12hr** | **15hr** | **18hr** | **21hr** | **24hr** | **27hr** | **30hr** |
| --- | --- | --- | --- | --- | --- | --- | --- | --- | --- | --- |
| Egg |  |  |  |  |  |  |  |  |  |  |
| Small Micromere |  |  |  |  |  |  |  |  |  |  |
| Skel. Micromere |  |  |  |  |  |  |  |  |  |  |
| Macromere |  |  |  |  |  |  |  |  |  |  |
| V2 |  |  |  |  |  |  |  |  |  |  |
| V2 Meso A |  |  |  |  |  |  |  |  |  |  |
| V2 Meso O |  |  |  |  |  |  |  |  |  |  |
| V2 Endo |  |  |  |  |  |  |  |  |  |  |
| V1 |  |  |  |  |  |  |  |  |  |  |
| V1 Endo A |  |  |  |  |  |  |  |  |  |  |
| V1 Endo O |  |  |  |  |  |  |  |  |  |  |
| V1 Ecto A |  |  |  |  |  |  |  |  |  |  |
| V1 Ecto O |  |  |  |  |  |  |  |  |  |  |
| Mesomere |  |  |  |  |  |  |  |  |  |  |
| Ecto A |  |  |  |  |  |  |  |  |  |  |
| Ecto O |  |  |  |  |  |  |  |  |  |  |
| Stomodeum |  |  |  |  |  |  |  |  |  |  |
| Oral Face |  |  |  |  |  |  |  |  |  |  |
| Ciliated Band |  |  |  |  |  |  |  |  |  |  |
| Apical Plate |  |  |  |  |  |  |  |  |  |  |

Predicted interaction: SPU_006991 (Lim1) → SPU_012877 (Kif27)

Absolute confidence value: 0.0000514259900300114

*Gene expression is not available for SPU_012877 (Kif27)

Result: missing complete spatial expression data

|  | **Node1: Lim1** | **Node2: Kif27*** | **Both expressed** |
| --- | --- | --- | --- |
| Weak expression |  |  |  |
| Expressed |  |  |  |

|  | **0hr** | **6hr** | **9hr** | **12hr** | **15hr** | **18hr** | **21hr** | **24hr** | **27hr** | **30hr** |
| --- | --- | --- | --- | --- | --- | --- | --- | --- | --- | --- |
| Egg |  |  |  |  |  |  |  |  |  |  |
| Small Micromere |  |  |  |  |  |  |  |  |  |  |
| Skel. Micromere |  |  |  |  |  |  |  |  |  |  |
| Macromere |  |  |  |  |  |  |  |  |  |  |
| V2 |  |  |  |  |  |  |  |  |  |  |
| V2 Meso A |  |  |  |  |  |  |  |  |  |  |
| V2 Meso O |  |  |  |  |  |  |  |  |  |  |
| V2 Endo |  |  |  |  |  |  |  |  |  |  |
| V1 |  |  |  |  |  |  |  |  |  |  |
| V1 Endo A |  |  |  |  |  |  |  |  |  |  |
| V1 Endo O |  |  |  |  |  |  |  |  |  |  |
| V1 Ecto A |  |  |  |  |  |  |  |  |  |  |
| V1 Ecto O |  |  |  |  |  |  |  |  |  |  |
| Mesomere |  |  |  |  |  |  |  |  |  |  |
| Ecto A |  |  |  |  |  |  |  |  |  |  |
| Ecto O |  |  |  |  |  |  |  |  |  |  |
| Stomodeum |  |  |  |  |  |  |  |  |  |  |
| Oral Face |  |  |  |  |  |  |  |  |  |  |
| Ciliated Band |  |  |  |  |  |  |  |  |  |  |
| Apical Plate |  |  |  |  |  |  |  |  |  |  |

Predicted interaction: SPU_011635 (FoxJ2_1) → SPU_016490 (Mtf1)

Absolute confidence value: 0.0000553071968247292

*Gene expression is not available for SPU_016490 (Mtf1)

Result: missing complete spatial expression data

|  | **Node1: FoxJ2_1** | **Node2: Mtf1*** | **Both expressed** |
| --- | --- | --- | --- |
| Weak expression |  |  |  |
| Expressed |  |  |  |

|  | **0hr** | **6hr** | **9hr** | **12hr** | **15hr** | **18hr** | **21hr** | **24hr** | **27hr** | **30hr** |
| --- | --- | --- | --- | --- | --- | --- | --- | --- | --- | --- |
| Egg |  |  |  |  |  |  |  |  |  |  |
| Small Micromere |  |  |  |  |  |  |  |  |  |  |
| Skel. Micromere |  |  |  |  |  |  |  |  |  |  |
| Macromere |  |  |  |  |  |  |  |  |  |  |
| V2 |  |  |  |  |  |  |  |  |  |  |
| V2 Meso A |  |  |  |  |  |  |  |  |  |  |
| V2 Meso O |  |  |  |  |  |  |  |  |  |  |
| V2 Endo |  |  |  |  |  |  |  |  |  |  |
| V1 |  |  |  |  |  |  |  |  |  |  |
| V1 Endo A |  |  |  |  |  |  |  |  |  |  |
| V1 Endo O |  |  |  |  |  |  |  |  |  |  |
| V1 Ecto A |  |  |  |  |  |  |  |  |  |  |
| V1 Ecto O |  |  |  |  |  |  |  |  |  |  |
| Mesomere |  |  |  |  |  |  |  |  |  |  |
| Ecto A |  |  |  |  |  |  |  |  |  |  |
| Ecto O |  |  |  |  |  |  |  |  |  |  |
| Stomodeum |  |  |  |  |  |  |  |  |  |  |
| Oral Face |  |  |  |  |  |  |  |  |  |  |
| Ciliated Band |  |  |  |  |  |  |  |  |  |  |
| Apical Plate |  |  |  |  |  |  |  |  |  |  |

Predicted interaction: SPU_000975 (FoxF) → SPU_011202 (Meis)

Absolute confidence value: 0.0000560002694666431

*Gene expression is not available for SPU_011202 (Meis)

Result: missing complete spatial expression data

|  | **Node1: FoxF** | **Node2: Meis*** | **Both expressed** |
| --- | --- | --- | --- |
| Weak expression |  |  |  |
| Expressed |  |  |  |

|  | **0hr** | **6hr** | **9hr** | **12hr** | **15hr** | **18hr** | **21hr** | **24hr** | **27hr** | **30hr** |
| --- | --- | --- | --- | --- | --- | --- | --- | --- | --- | --- |
| Egg |  |  |  |  |  |  |  |  |  |  |
| Small Micromere |  |  |  |  |  |  |  |  |  |  |
| Skel. Micromere |  |  |  |  |  |  |  |  |  |  |
| Macromere |  |  |  |  |  |  |  |  |  |  |
| V2 |  |  |  |  |  |  |  |  |  |  |
| V2 Meso A |  |  |  |  |  |  |  |  |  |  |
| V2 Meso O |  |  |  |  |  |  |  |  |  |  |
| V2 Endo |  |  |  |  |  |  |  |  |  |  |
| V1 |  |  |  |  |  |  |  |  |  |  |
| V1 Endo A |  |  |  |  |  |  |  |  |  |  |
| V1 Endo O |  |  |  |  |  |  |  |  |  |  |
| V1 Ecto A |  |  |  |  |  |  |  |  |  |  |
| V1 Ecto O |  |  |  |  |  |  |  |  |  |  |
| Mesomere |  |  |  |  |  |  |  |  |  |  |
| Ecto A |  |  |  |  |  |  |  |  |  |  |
| Ecto O |  |  |  |  |  |  |  |  |  |  |
| Stomodeum |  |  |  |  |  |  |  |  |  |  |
| Oral Face |  |  |  |  |  |  |  |  |  |  |
| Ciliated Band |  |  |  |  |  |  |  |  |  |  |
| Apical Plate |  |  |  |  |  |  |  |  |  |  |

Predicted interaction: SPU_012203 (Rel) → SPU_004551 (FoxB)

Absolute confidence value: 0.0000560002694666431

*Gene expression is not available for SPU_012203 (Rel)

Result: missing complete spatial expression data

|  | **Node1: Rel*** | **Node2: FoxB** | **Both expressed** |
| --- | --- | --- | --- |
| Weak expression |  |  |  |
| Expressed |  |  |  |

|  | **0hr** | **6hr** | **9hr** | **12hr** | **15hr** | **18hr** | **21hr** | **24hr** | **27hr** | **30hr** |
| --- | --- | --- | --- | --- | --- | --- | --- | --- | --- | --- |
| Egg |  |  |  |  |  |  |  |  |  |  |
| Small Micromere |  |  |  |  |  |  |  |  |  |  |
| Skel. Micromere |  |  |  |  |  |  |  |  |  |  |
| Macromere |  |  |  |  |  |  |  |  |  |  |
| V2 |  |  |  |  |  |  |  |  |  |  |
| V2 Meso A |  |  |  |  |  |  |  |  |  |  |
| V2 Meso O |  |  |  |  |  |  |  |  |  |  |
| V2 Endo |  |  |  |  |  |  |  |  |  |  |
| V1 |  |  |  |  |  |  |  |  |  |  |
| V1 Endo A |  |  |  |  |  |  |  |  |  |  |
| V1 Endo O |  |  |  |  |  |  |  |  |  |  |
| V1 Ecto A |  |  |  |  |  |  |  |  |  |  |
| V1 Ecto O |  |  |  |  |  |  |  |  |  |  |
| Mesomere |  |  |  |  |  |  |  |  |  |  |
| Ecto A |  |  |  |  |  |  |  |  |  |  |
| Ecto O |  |  |  |  |  |  |  |  |  |  |
| Stomodeum |  |  |  |  |  |  |  |  |  |  |
| Oral Face |  |  |  |  |  |  |  |  |  |  |
| Ciliated Band |  |  |  |  |  |  |  |  |  |  |
| Apical Plate |  |  |  |  |  |  |  |  |  |  |

Predicted interaction: SPU_019332 (Ppar1) → SPU_008953 (Talpid3)

Absolute confidence value: 0.0000619606941871027

*Gene expression is not available for SPU_019332 (Ppar1) or SPU_008953 (Talpid3)

Result: missing complete spatial expression data

|  | **Node1: Ppar1*** | **Node2: Talpid3*** | **Both expressed** |
| --- | --- | --- | --- |
| Weak expression |  |  |  |
| Expressed |  |  |  |

|  | **0hr** | **6hr** | **9hr** | **12hr** | **15hr** | **18hr** | **21hr** | **24hr** | **27hr** | **30hr** |
| --- | --- | --- | --- | --- | --- | --- | --- | --- | --- | --- |
| Egg |  |  |  |  |  |  |  |  |  |  |
| Small Micromere |  |  |  |  |  |  |  |  |  |  |
| Skel. Micromere |  |  |  |  |  |  |  |  |  |  |
| Macromere |  |  |  |  |  |  |  |  |  |  |
| V2 |  |  |  |  |  |  |  |  |  |  |
| V2 Meso A |  |  |  |  |  |  |  |  |  |  |
| V2 Meso O |  |  |  |  |  |  |  |  |  |  |
| V2 Endo |  |  |  |  |  |  |  |  |  |  |
| V1 |  |  |  |  |  |  |  |  |  |  |
| V1 Endo A |  |  |  |  |  |  |  |  |  |  |
| V1 Endo O |  |  |  |  |  |  |  |  |  |  |
| V1 Ecto A |  |  |  |  |  |  |  |  |  |  |
| V1 Ecto O |  |  |  |  |  |  |  |  |  |  |
| Mesomere |  |  |  |  |  |  |  |  |  |  |
| Ecto A |  |  |  |  |  |  |  |  |  |  |
| Ecto O |  |  |  |  |  |  |  |  |  |  |
| Stomodeum |  |  |  |  |  |  |  |  |  |  |
| Oral Face |  |  |  |  |  |  |  |  |  |  |
| Ciliated Band |  |  |  |  |  |  |  |  |  |  |
| Apical Plate |  |  |  |  |  |  |  |  |  |  |

Predicted interaction: SPU_015374 (Id) → SPU_030139 (Igf2)

Absolute confidence value: 0.0000619606941871027

*Gene expression is not available for SPU_015374 (Id) or SPU_030139 (Igf2)

Result: missing complete spatial expression data

|  | **Node1: Id*** | **Node2: Igf2*** | **Both expressed** |
| --- | --- | --- | --- |
| Weak expression |  |  |  |
| Expressed |  |  |  |

|  | **0hr** | **6hr** | **9hr** | **12hr** | **15hr** | **18hr** | **21hr** | **24hr** | **27hr** | **30hr** |
| --- | --- | --- | --- | --- | --- | --- | --- | --- | --- | --- |
| Egg |  |  |  |  |  |  |  |  |  |  |
| Small Micromere |  |  |  |  |  |  |  |  |  |  |
| Skel. Micromere |  |  |  |  |  |  |  |  |  |  |
| Macromere |  |  |  |  |  |  |  |  |  |  |
| V2 |  |  |  |  |  |  |  |  |  |  |
| V2 Meso A |  |  |  |  |  |  |  |  |  |  |
| V2 Meso O |  |  |  |  |  |  |  |  |  |  |
| V2 Endo |  |  |  |  |  |  |  |  |  |  |
| V1 |  |  |  |  |  |  |  |  |  |  |
| V1 Endo A |  |  |  |  |  |  |  |  |  |  |
| V1 Endo O |  |  |  |  |  |  |  |  |  |  |
| V1 Ecto A |  |  |  |  |  |  |  |  |  |  |
| V1 Ecto O |  |  |  |  |  |  |  |  |  |  |
| Mesomere |  |  |  |  |  |  |  |  |  |  |
| Ecto A |  |  |  |  |  |  |  |  |  |  |
| Ecto O |  |  |  |  |  |  |  |  |  |  |
| Stomodeum |  |  |  |  |  |  |  |  |  |  |
| Oral Face |  |  |  |  |  |  |  |  |  |  |
| Ciliated Band |  |  |  |  |  |  |  |  |  |  |
| Apical Plate |  |  |  |  |  |  |  |  |  |  |

Predicted interaction: SPU_003595 (Cutl) → SPU_024953 (Z34)

Absolute confidence value: 0.0000620993087154854

*Gene expression is not available for SPU_003595 (Cutl) and SPU_024953 (Z34)

Result: missing complete spatial expression data

|  | **Node1: Cutl*** | **Node2: Z34*** | **Both expressed** |
| --- | --- | --- | --- |
| Weak expression |  |  |  |
| Expressed |  |  |  |

|  | **0hr** | **6hr** | **9hr** | **12hr** | **15hr** | **18hr** | **21hr** | **24hr** | **27hr** | **30hr** |
| --- | --- | --- | --- | --- | --- | --- | --- | --- | --- | --- |
| Egg |  |  |  |  |  |  |  |  |  |  |
| Small Micromere |  |  |  |  |  |  |  |  |  |  |
| Skel. Micromere |  |  |  |  |  |  |  |  |  |  |
| Macromere |  |  |  |  |  |  |  |  |  |  |
| V2 |  |  |  |  |  |  |  |  |  |  |
| V2 Meso A |  |  |  |  |  |  |  |  |  |  |
| V2 Meso O |  |  |  |  |  |  |  |  |  |  |
| V2 Endo |  |  |  |  |  |  |  |  |  |  |
| V1 |  |  |  |  |  |  |  |  |  |  |
| V1 Endo A |  |  |  |  |  |  |  |  |  |  |
| V1 Endo O |  |  |  |  |  |  |  |  |  |  |
| V1 Ecto A |  |  |  |  |  |  |  |  |  |  |
| V1 Ecto O |  |  |  |  |  |  |  |  |  |  |
| Mesomere |  |  |  |  |  |  |  |  |  |  |
| Ecto A |  |  |  |  |  |  |  |  |  |  |
| Ecto O |  |  |  |  |  |  |  |  |  |  |
| Stomodeum |  |  |  |  |  |  |  |  |  |  |
| Oral Face |  |  |  |  |  |  |  |  |  |  |
| Ciliated Band |  |  |  |  |  |  |  |  |  |  |
| Apical Plate |  |  |  |  |  |  |  |  |  |  |

Predicted interaction: SPU_007981 (Lmpt) → SPU_008747 (Fused)

Absolute confidence value: 0.0000632082249425477

*Gene expression is not available for SPU_007981 (Lmpt) and SPU_008747 (Fused)

Result: missing complete spatial expression data

|  | **Node1: Lmpt*** | **Node2: Fused*** | **Both expressed** |
| --- | --- | --- | --- |
| Weak expression |  |  |  |
| Expressed |  |  |  |

|  | **0hr** | **6hr** | **9hr** | **12hr** | **15hr** | **18hr** | **21hr** | **24hr** | **27hr** | **30hr** |
| --- | --- | --- | --- | --- | --- | --- | --- | --- | --- | --- |
| Egg |  |  |  |  |  |  |  |  |  |  |
| Small Micromere |  |  |  |  |  |  |  |  |  |  |
| Skel. Micromere |  |  |  |  |  |  |  |  |  |  |
| Macromere |  |  |  |  |  |  |  |  |  |  |
| V2 |  |  |  |  |  |  |  |  |  |  |
| V2 Meso A |  |  |  |  |  |  |  |  |  |  |
| V2 Meso O |  |  |  |  |  |  |  |  |  |  |
| V2 Endo |  |  |  |  |  |  |  |  |  |  |
| V1 |  |  |  |  |  |  |  |  |  |  |
| V1 Endo A |  |  |  |  |  |  |  |  |  |  |
| V1 Endo O |  |  |  |  |  |  |  |  |  |  |
| V1 Ecto A |  |  |  |  |  |  |  |  |  |  |
| V1 Ecto O |  |  |  |  |  |  |  |  |  |  |
| Mesomere |  |  |  |  |  |  |  |  |  |  |
| Ecto A |  |  |  |  |  |  |  |  |  |  |
| Ecto O |  |  |  |  |  |  |  |  |  |  |
| Stomodeum |  |  |  |  |  |  |  |  |  |  |
| Oral Face |  |  |  |  |  |  |  |  |  |  |
| Ciliated Band |  |  |  |  |  |  |  |  |  |  |
| Apical Plate |  |  |  |  |  |  |  |  |  |  |

Predicted interaction: SPU_014793 (Spalt) → SPU_005522 (Ift172_3)

Absolute confidence value: 0.000063624068527696

*Gene expression is not available for SPU_014793 (Spalt) or SPU_005522 (Ift172_3)

Result: missing complete spatial expression data

|  | **Node1: Spalt*** | **Node2: Ift172_3*** | **Both expressed** |
| --- | --- | --- | --- |
| Weak expression |  |  |  |
| Expressed |  |  |  |

|  | **0hr** | **6hr** | **9hr** | **12hr** | **15hr** | **18hr** | **21hr** | **24hr** | **27hr** | **30hr** |
| --- | --- | --- | --- | --- | --- | --- | --- | --- | --- | --- |
| Egg |  |  |  |  |  |  |  |  |  |  |
| Small Micromere |  |  |  |  |  |  |  |  |  |  |
| Skel. Micromere |  |  |  |  |  |  |  |  |  |  |
| Macromere |  |  |  |  |  |  |  |  |  |  |
| V2 |  |  |  |  |  |  |  |  |  |  |
| V2 Meso A |  |  |  |  |  |  |  |  |  |  |
| V2 Meso O |  |  |  |  |  |  |  |  |  |  |
| V2 Endo |  |  |  |  |  |  |  |  |  |  |
| V1 |  |  |  |  |  |  |  |  |  |  |
| V1 Endo A |  |  |  |  |  |  |  |  |  |  |
| V1 Endo O |  |  |  |  |  |  |  |  |  |  |
| V1 Ecto A |  |  |  |  |  |  |  |  |  |  |
| V1 Ecto O |  |  |  |  |  |  |  |  |  |  |
| Mesomere |  |  |  |  |  |  |  |  |  |  |
| Ecto A |  |  |  |  |  |  |  |  |  |  |
| Ecto O |  |  |  |  |  |  |  |  |  |  |
| Stomodeum |  |  |  |  |  |  |  |  |  |  |
| Oral Face |  |  |  |  |  |  |  |  |  |  |
| Ciliated Band |  |  |  |  |  |  |  |  |  |  |
| Apical Plate |  |  |  |  |  |  |  |  |  |  |

Predicted interaction: SPU_011635 (FoxJ2_1) or SPU_014687 (Z310_1)

Absolute confidence value: 0.0000640399121128444

*Gene expressions is not available for SPU_014687 (Z310_1)

Result: missing complete spatial expression data

|  | **Node1: FoxJ2_1** | **Node2: Z310_1*** | **Both expressed** |
| --- | --- | --- | --- |
| Weak expression |  |  |  |
| Expressed |  |  |  |

|  | **0hr** | **6hr** | **9hr** | **12hr** | **15hr** | **18hr** | **21hr** | **24hr** | **27hr** | **30hr** |
| --- | --- | --- | --- | --- | --- | --- | --- | --- | --- | --- |
| Egg |  |  |  |  |  |  |  |  |  |  |
| Small Micromere |  |  |  |  |  |  |  |  |  |  |
| Skel. Micromere |  |  |  |  |  |  |  |  |  |  |
| Macromere |  |  |  |  |  |  |  |  |  |  |
| V2 |  |  |  |  |  |  |  |  |  |  |
| V2 Meso A |  |  |  |  |  |  |  |  |  |  |
| V2 Meso O |  |  |  |  |  |  |  |  |  |  |
| V2 Endo |  |  |  |  |  |  |  |  |  |  |
| V1 |  |  |  |  |  |  |  |  |  |  |
| V1 Endo A |  |  |  |  |  |  |  |  |  |  |
| V1 Endo O |  |  |  |  |  |  |  |  |  |  |
| V1 Ecto A |  |  |  |  |  |  |  |  |  |  |
| V1 Ecto O |  |  |  |  |  |  |  |  |  |  |
| Mesomere |  |  |  |  |  |  |  |  |  |  |
| Ecto A |  |  |  |  |  |  |  |  |  |  |
| Ecto O |  |  |  |  |  |  |  |  |  |  |
| Stomodeum |  |  |  |  |  |  |  |  |  |  |
| Oral Face |  |  |  |  |  |  |  |  |  |  |
| Ciliated Band |  |  |  |  |  |  |  |  |  |  |
| Apical Plate |  |  |  |  |  |  |  |  |  |  |

Predicted interaction: SPU_015828 (Pmar1c) → SPU_022955 (Z76)

Absolute confidence value: 0.000065564671925055

*Gene expression is not available for SPU_022955 (Z76)

Result: missing complete spatial expression data

|  | **Node1: Pmar1c** | **Node2: Z76*** | **Both expressed** |
| --- | --- | --- | --- |
| Weak expression |  |  |  |
| Expressed |  |  |  |

|  | **0hr** | **6hr** | **9hr** | **12hr** | **15hr** | **18hr** | **21hr** | **24hr** | **27hr** | **30hr** |
| --- | --- | --- | --- | --- | --- | --- | --- | --- | --- | --- |
| Egg |  |  |  |  |  |  |  |  |  |  |
| Small Micromere |  |  |  |  |  |  |  |  |  |  |
| Skel. Micromere |  |  |  |  |  |  |  |  |  |  |
| Macromere |  |  |  |  |  |  |  |  |  |  |
| V2 |  |  |  |  |  |  |  |  |  |  |
| V2 Meso A |  |  |  |  |  |  |  |  |  |  |
| V2 Meso O |  |  |  |  |  |  |  |  |  |  |
| V2 Endo |  |  |  |  |  |  |  |  |  |  |
| V1 |  |  |  |  |  |  |  |  |  |  |
| V1 Endo A |  |  |  |  |  |  |  |  |  |  |
| V1 Endo O |  |  |  |  |  |  |  |  |  |  |
| V1 Ecto A |  |  |  |  |  |  |  |  |  |  |
| V1 Ecto O |  |  |  |  |  |  |  |  |  |  |
| Mesomere |  |  |  |  |  |  |  |  |  |  |
| Ecto A |  |  |  |  |  |  |  |  |  |  |
| Ecto O |  |  |  |  |  |  |  |  |  |  |
| Stomodeum |  |  |  |  |  |  |  |  |  |  |
| Oral Face |  |  |  |  |  |  |  |  |  |  |
| Ciliated Band |  |  |  |  |  |  |  |  |  |  |
| Apical Plate |  |  |  |  |  |  |  |  |  |  |

Predicted interaction: SPU_011246 (IrxB) → SPU_002428 (Mib2)

Absolute confidence value: 0.0000672280462656483

*Gene expression is not available for SPU_011246 (IrxB) or SPU_002428 (Mib2)

Result: missing complete spatial expression data

|  | **Node1: IrxB*** | **Node2: Mib2*** | **Both expressed** |
| --- | --- | --- | --- |
| Weak expression |  |  |  |
| Expressed |  |  |  |

|  | **0hr** | **6hr** | **9hr** | **12hr** | **15hr** | **18hr** | **21hr** | **24hr** | **27hr** | **30hr** |
| --- | --- | --- | --- | --- | --- | --- | --- | --- | --- | --- |
| Egg |  |  |  |  |  |  |  |  |  |  |
| Small Micromere |  |  |  |  |  |  |  |  |  |  |
| Skel. Micromere |  |  |  |  |  |  |  |  |  |  |
| Macromere |  |  |  |  |  |  |  |  |  |  |
| V2 |  |  |  |  |  |  |  |  |  |  |
| V2 Meso A |  |  |  |  |  |  |  |  |  |  |
| V2 Meso O |  |  |  |  |  |  |  |  |  |  |
| V2 Endo |  |  |  |  |  |  |  |  |  |  |
| V1 |  |  |  |  |  |  |  |  |  |  |
| V1 Endo A |  |  |  |  |  |  |  |  |  |  |
| V1 Endo O |  |  |  |  |  |  |  |  |  |  |
| V1 Ecto A |  |  |  |  |  |  |  |  |  |  |
| V1 Ecto O |  |  |  |  |  |  |  |  |  |  |
| Mesomere |  |  |  |  |  |  |  |  |  |  |
| Ecto A |  |  |  |  |  |  |  |  |  |  |
| Ecto O |  |  |  |  |  |  |  |  |  |  |
| Stomodeum |  |  |  |  |  |  |  |  |  |  |
| Oral Face |  |  |  |  |  |  |  |  |  |  |
| Ciliated Band |  |  |  |  |  |  |  |  |  |  |
| Apical Plate |  |  |  |  |  |  |  |  |  |  |

Predicted interaction: SPU_026763 (Scmh1) → SPU_019825 (Z352)

Absolute confidence value: 0.0000679211189075622

*Gene expression is not available for SPU_026763 (Scmh1) or SPU_019825 (Z352)

Result: missing complete spatial expression data

|  | **Node1: Scmh1*** | **Node2: Z352*** | **Both expressed** |
| --- | --- | --- | --- |
| Weak expression |  |  |  |
| Expressed |  |  |  |

|  | **0hr** | **6hr** | **9hr** | **12hr** | **15hr** | **18hr** | **21hr** | **24hr** | **27hr** | **30hr** |
| --- | --- | --- | --- | --- | --- | --- | --- | --- | --- | --- |
| Egg |  |  |  |  |  |  |  |  |  |  |
| Small Micromere |  |  |  |  |  |  |  |  |  |  |
| Skel. Micromere |  |  |  |  |  |  |  |  |  |  |
| Macromere |  |  |  |  |  |  |  |  |  |  |
| V2 |  |  |  |  |  |  |  |  |  |  |
| V2 Meso A |  |  |  |  |  |  |  |  |  |  |
| V2 Meso O |  |  |  |  |  |  |  |  |  |  |
| V2 Endo |  |  |  |  |  |  |  |  |  |  |
| V1 |  |  |  |  |  |  |  |  |  |  |
| V1 Endo A |  |  |  |  |  |  |  |  |  |  |
| V1 Endo O |  |  |  |  |  |  |  |  |  |  |
| V1 Ecto A |  |  |  |  |  |  |  |  |  |  |
| V1 Ecto O |  |  |  |  |  |  |  |  |  |  |
| Mesomere |  |  |  |  |  |  |  |  |  |  |
| Ecto A |  |  |  |  |  |  |  |  |  |  |
| Ecto O |  |  |  |  |  |  |  |  |  |  |
| Stomodeum |  |  |  |  |  |  |  |  |  |  |
| Oral Face |  |  |  |  |  |  |  |  |  |  |
| Ciliated Band |  |  |  |  |  |  |  |  |  |  |
| Apical Plate |  |  |  |  |  |  |  |  |  |  |

Predicted interaction: SPU_006676 (FoxA) → SPU_027144 (Pik3ca)

Absolute confidence value: 0.0000686141915494761

*Gene expression is not available for SPU_027144 (Pik3ca)

Result: missing complete spatial expression data

|  | **Node1: FoxA** | **Node2: Pik3ca*** | **Both expressed** |
| --- | --- | --- | --- |
| Weak expression |  |  |  |
| Expressed |  |  |  |

|  | **0hr** | **6hr** | **9hr** | **12hr** | **15hr** | **18hr** | **21hr** | **24hr** | **27hr** | **30hr** |
| --- | --- | --- | --- | --- | --- | --- | --- | --- | --- | --- |
| Egg |  |  |  |  |  |  |  |  |  |  |
| Small Micromere |  |  |  |  |  |  |  |  |  |  |
| Skel. Micromere |  |  |  |  |  |  |  |  |  |  |
| Macromere |  |  |  |  |  |  |  |  |  |  |
| V2 |  |  |  |  |  |  |  |  |  |  |
| V2 Meso A |  |  |  |  |  |  |  |  |  |  |
| V2 Meso O |  |  |  |  |  |  |  |  |  |  |
| V2 Endo |  |  |  |  |  |  |  |  |  |  |
| V1 |  |  |  |  |  |  |  |  |  |  |
| V1 Endo A |  |  |  |  |  |  |  |  |  |  |
| V1 Endo O |  |  |  |  |  |  |  |  |  |  |
| V1 Ecto A |  |  |  |  |  |  |  |  |  |  |
| V1 Ecto O |  |  |  |  |  |  |  |  |  |  |
| Mesomere |  |  |  |  |  |  |  |  |  |  |
| Ecto A |  |  |  |  |  |  |  |  |  |  |
| Ecto O |  |  |  |  |  |  |  |  |  |  |
| Stomodeum |  |  |  |  |  |  |  |  |  |  |
| Oral Face |  |  |  |  |  |  |  |  |  |  |
| Ciliated Band |  |  |  |  |  |  |  |  |  |  |
| Apical Plate |  |  |  |  |  |  |  |  |  |  |
